# Supplementary material for: Vacuolar Protein Sorting 35 Controls Hepatocellular Proliferation Through SRC Signaling and Promotes Diethyl Nitrosamine–Induced Tumor Initiation
Source: Cell Mol Gastroenterol Hepatol. 2026 May 5;20(8):101788. doi: 10.1016/j.jcmgh.2026.101788 (PMC13310651; doi:10.1016/j.jcmgh.2026.101788)
Supplement: Supplementary Tables [file mmc1.pdf]

Supplementary Tables

Supplementary Table 1. Differentially expressed genes related to mechanosensitive transcriptional co-factor YES-associated protein (YAP)-target genes and SRC kinase family genes in *Vps35*^{HepKO} livers compared to controls, as determined with RNAseq analysis.

Gene	Gene name	Log2(FC)	Adj. *P* value
Areg	Amphiregulin	3.415	0.003
Birc5	Baculoviral IAP repeat-containing 5	1.417	8.017E-04
Itgβ2	Integrin beta 2	1.429	2.633E-04
Sox9	SRY (sex determining region Y)-box 9	0.968	0.013
Alb	Albumin	-0.444	0.016
Src	Sarcoma oncogene	1.687	5.15E-08
Fgr	FGR proto-oncogene	1.661	2.52E-09
Hck	Hemopoeietic cell kinase	1.453	8.87E-06

Supplementary Table 2. Sequences of primers used for qRT-PCR analysis.

Gene	Forward primer (5'-3')	Reverse primer (5'-3')
Areg	TTGCTGCTGGTCTTAGGCTC	TGGTCCCCAGAAAGCGATTC
Birc5	ATCCACTGCCCTACCGAGAA	CTTGGCTCTCTGTCTGTCCAGTT
Cyr61	AGAGGCTTCCTGTCTTTGGC	CCAAGACGTGGTCTGAACGA
Itgβ2	GCTTTGGGTCGTTTGTGGAC	TGCCGACCTCTGTCTGAAAC
Ppia	TTCCTCCTTTCACAGAATTATTCCA	CCGCCAGTGCCATTATGG
Alb	TGCTTTTTCCAGGGGTGTGTT	TTACTTCCTGCACTAATTTGGCA

Supplementary Table 3. Antibodies used for immunoblotting analysis.

Target	Company	Catalog #	Dilution
AKT	Cell Signaling	4691	1:1000
p-AKT (Ser473)	Cell Signaling	9271	1:1000
Integrin alpha 5 (ITGA5)	Cell Signaling	4705	1:1000
β-actin	Sigma	A5441	1:1000
β-catenin	Abcam	ab6302	1:1000
EGFR	Santa Cruz	sc-373746	1:1000
p-EGFR	Cell Signaling	3777	1:1000
ERK	Cell Signaling	4370	1:1000
p-ERK	Cell Signaling	4695	1:1000
GAPDH	Abcam	ab8245	1:1000
HSP90	Cell Signaling	4874	1:1000
IGFR	Cell Signaling	3027	1:1000
p-IGFR	Cell Signaling	28897	1:1000
Lamin A/C	Cell Signaling	2032	1:1000
p-STAT3 (Tyr705)	Cell Signaling	9145	1:1000
STAT3	Cell Signalling	9139	1:1000
p-SRC (Tyr416)	Cell Signaling	2101	1:1000
SRC	Cell Signaling	2108	1:1000
Vinculin	Abcam	ab129002	1:1000
VPS35	Abcam	ab10099	1:1000
goat anti-mouse IgG (H+L)-HRP conjugate	Bio-Rad	170-6516	1:10000
goat anti-rabbit IgG (H+L)-HRP conjugate	Bio-Rad	170-6515	1:10000

Supplementary Table 4. Antibodies used for histological analysis.

Target	Company	Catalog #	Dilution
BrdU	Dako	M0744	1:800
Ki67	Thermo Fisher	RM-9106	1:50
p21	Abcam	ab107099	1:400
H2AX (ser139)	Cell Signalling	9718S	1:250
PCNA	Dako	M0879	1:600
Active Caspase-3	R&D Systems	AF835	1:600
Goat anti-rabbit (biotinylated)	Vector	BA-1000	1:250
Goat anti-mouse (biotinylated)	Vector	BA-9200	1:125
Goat anti-rat (biotinylated)	Vector	BA-9400	1:125
