# Supplementary material for: Vacuolar Protein Sorting 35 Controls Hepatocellular Proliferation Through SRC Signaling and Promotes Diethyl Nitrosamine–Induced Tumor Initiation
Source: Cell Mol Gastroenterol Hepatol. 2026 May 5;20(8):101788. doi: 10.1016/j.jcmgh.2026.101788 (PMC13310651; doi:10.1016/j.jcmgh.2026.101788)
Supplement: Supplementary Material [file mmc2.pdf]

Figure 2D

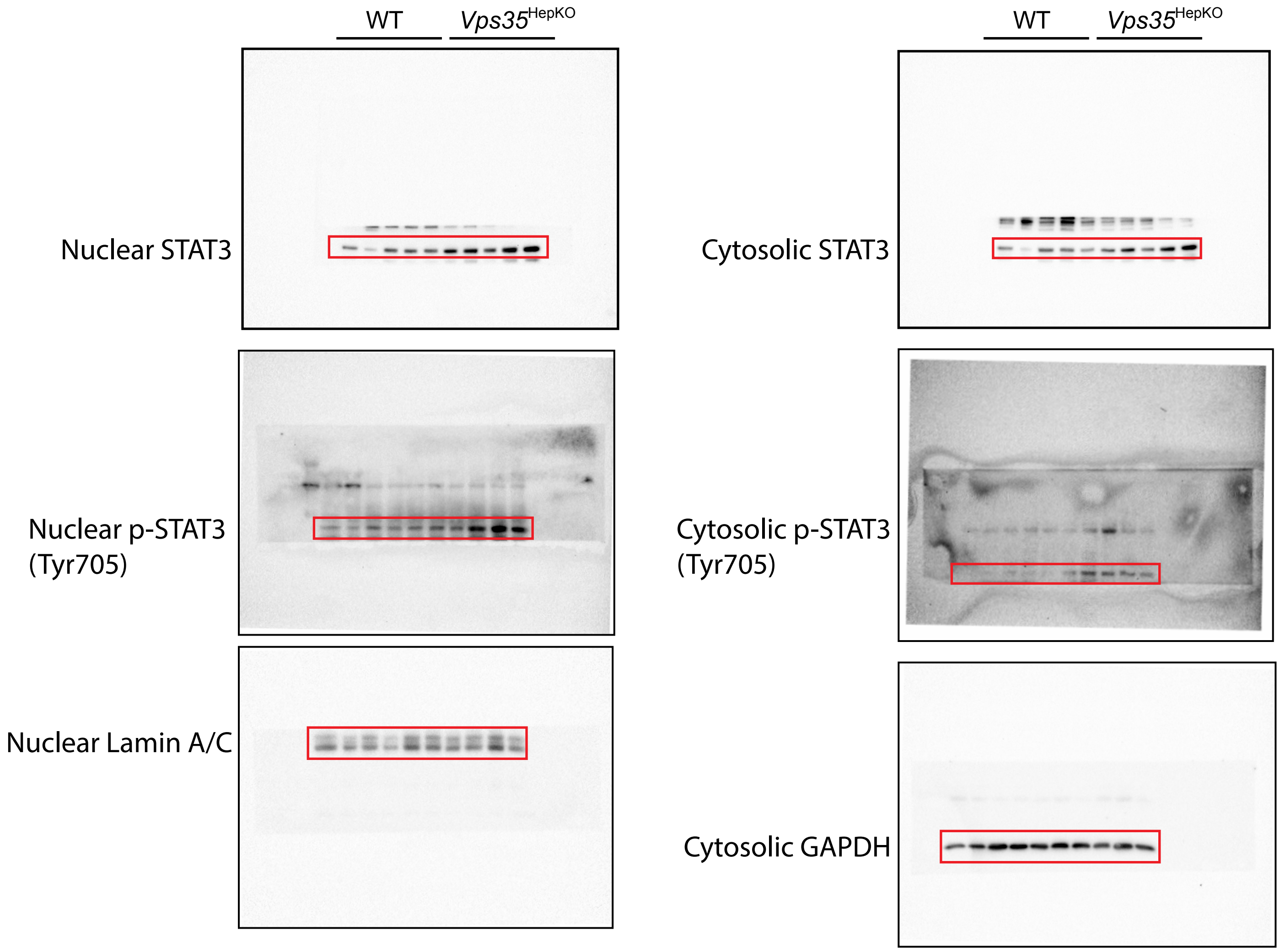
WT
Vps35HepKO
Nuclear STAT3
Nuclear p-STAT3 (Tyr705)
Nuclear Lamin A/C
WT
Vps35HepKO
Cytosolic STAT3
Cytosolic p-STAT3 (Tyr705)
Cytosolic GAPDH

Figure 3C + 3E

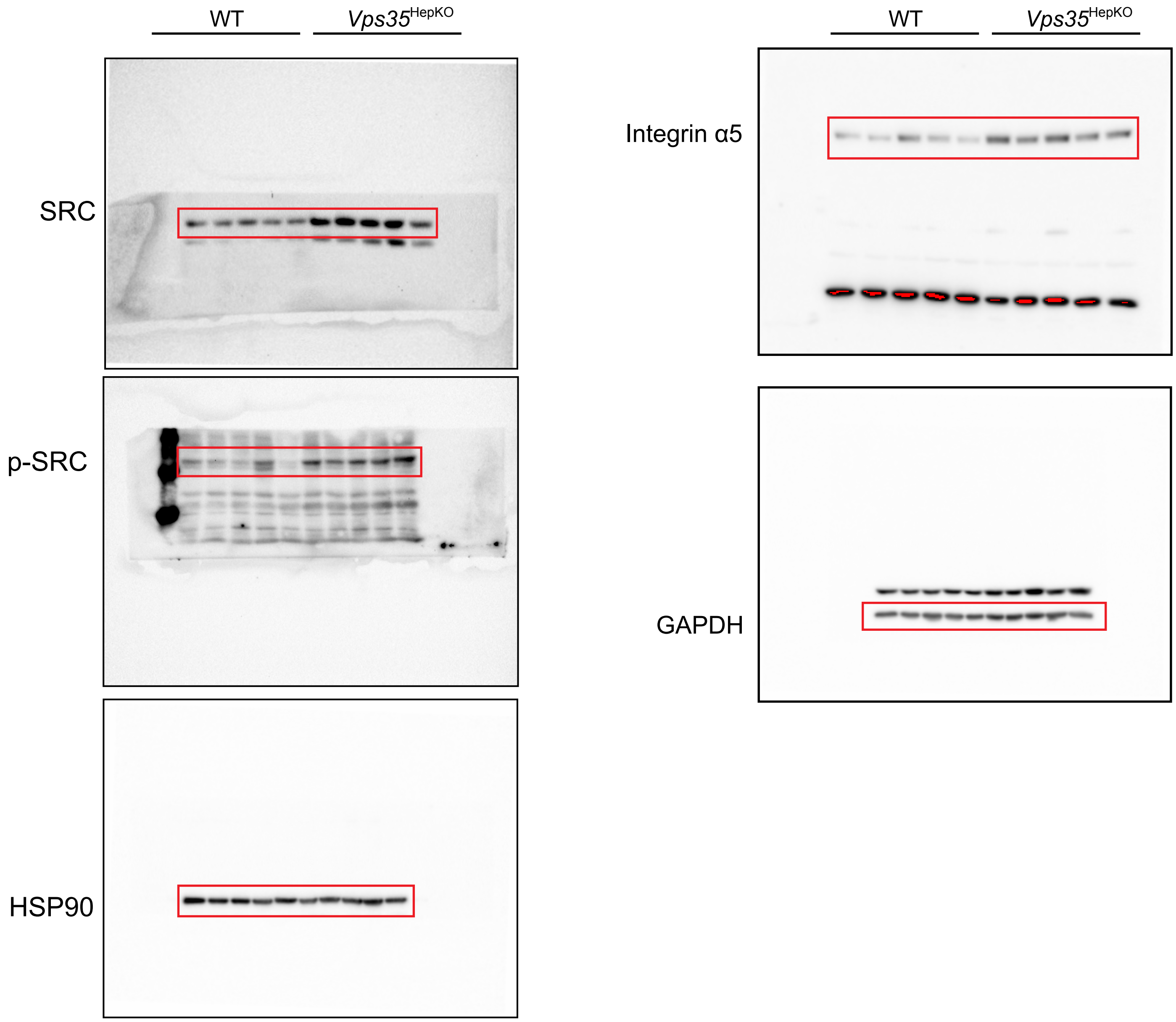
WT
Vps35HepKO
SRC
p-SRC
HSP90
WT
Vps35HepKO
Integrin α5
GAPDH

Figure 4B

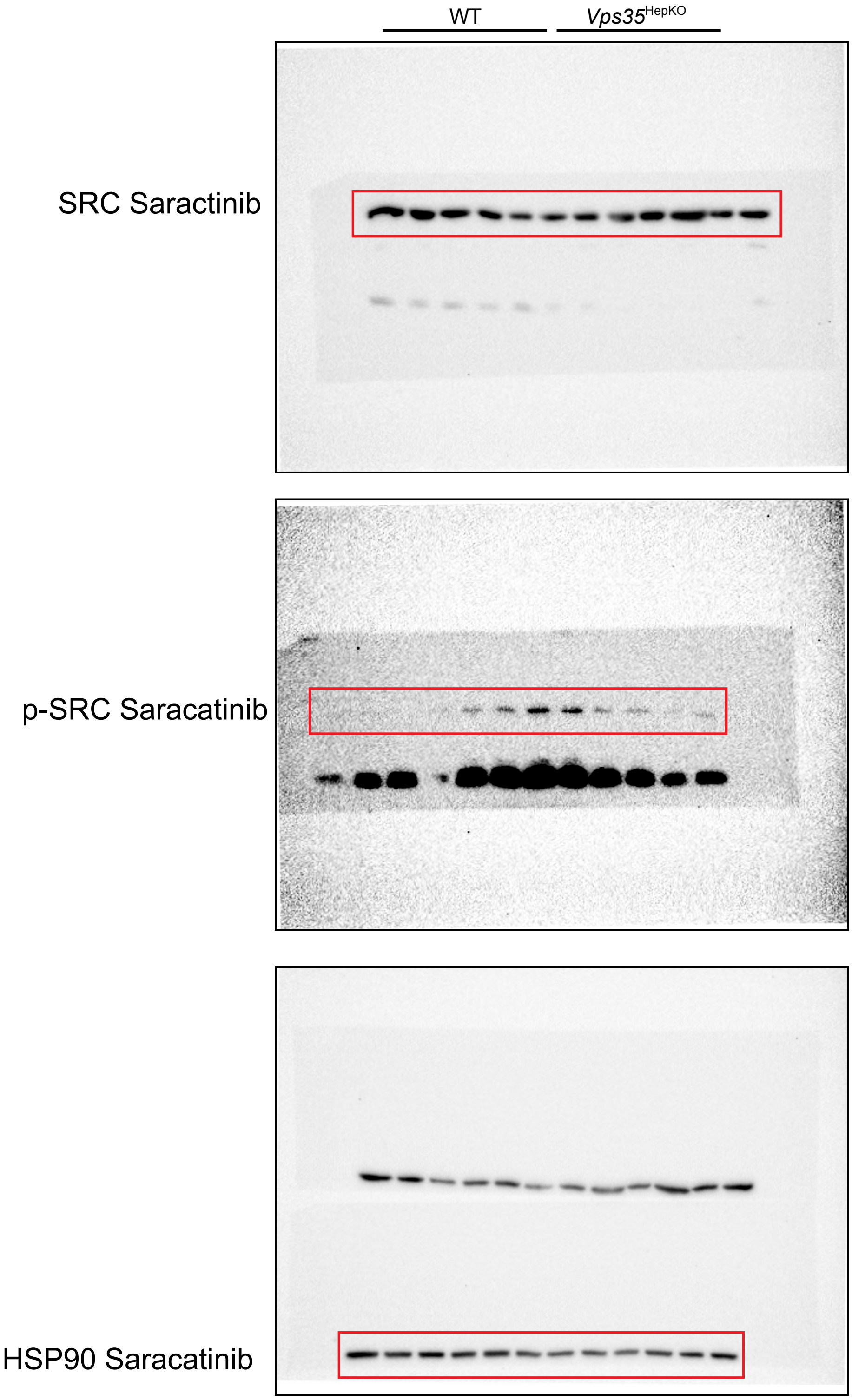
WT
Vps35HepKO
SRC Saractinib
p-SRC Saracatinib
HSP90 Saracatinib

Figure 6A

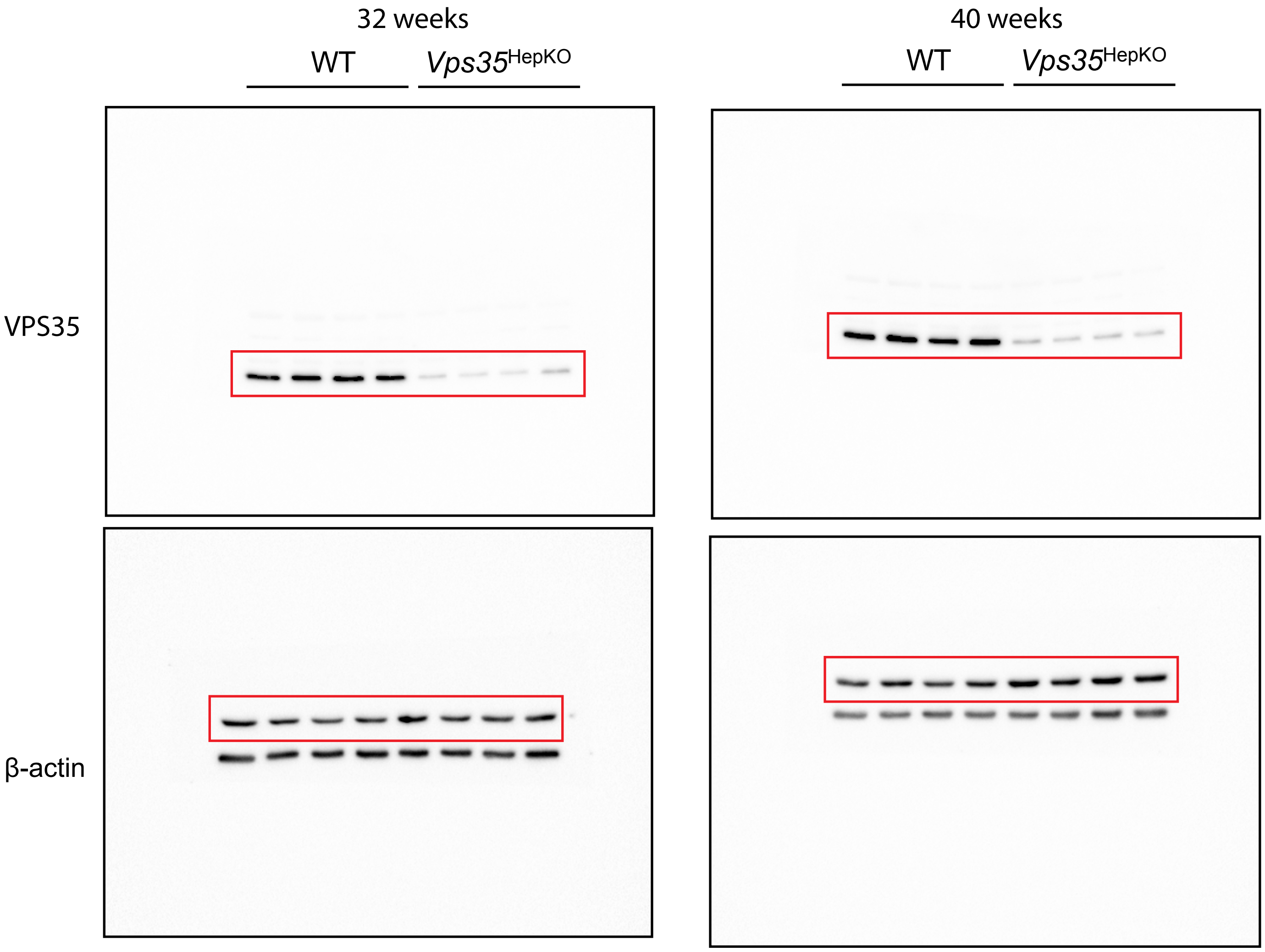

Figure 6H

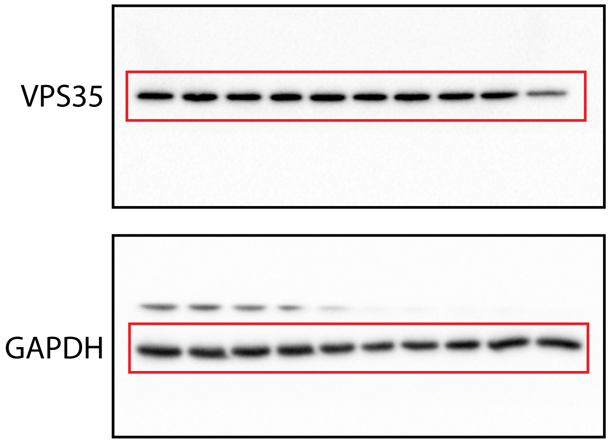

Supplemental Figure 2

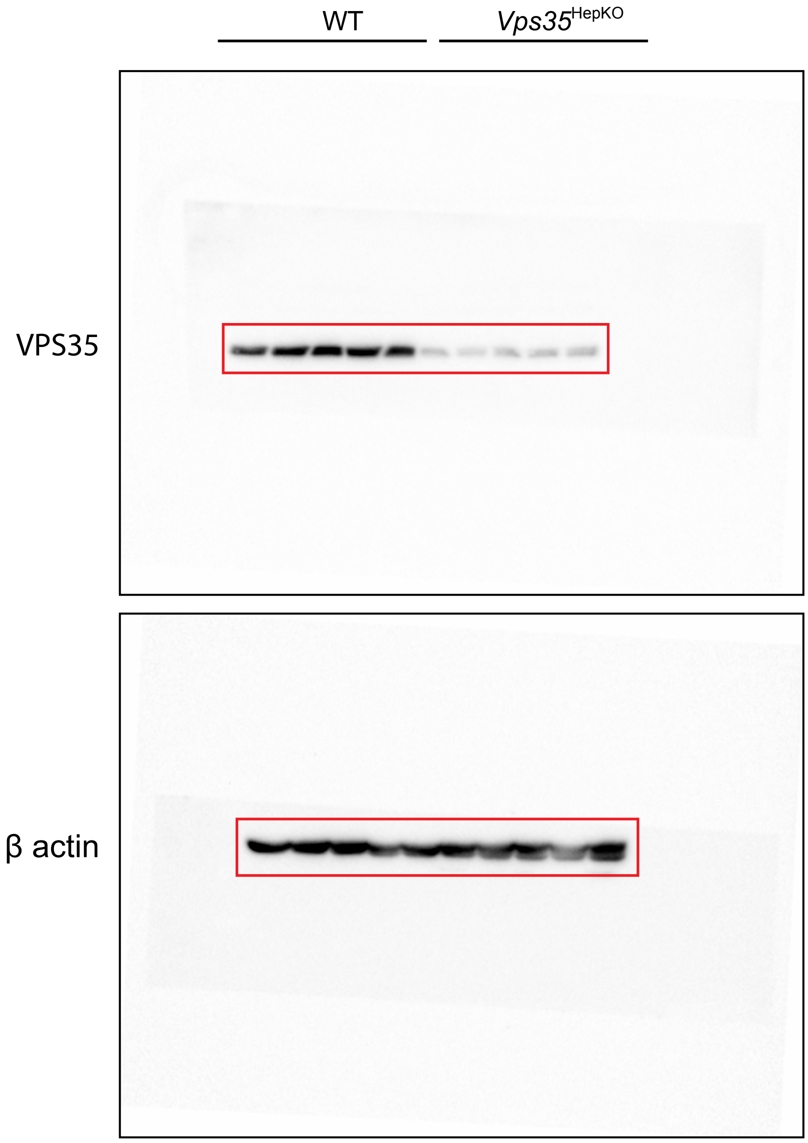

Supplemental Figure 3

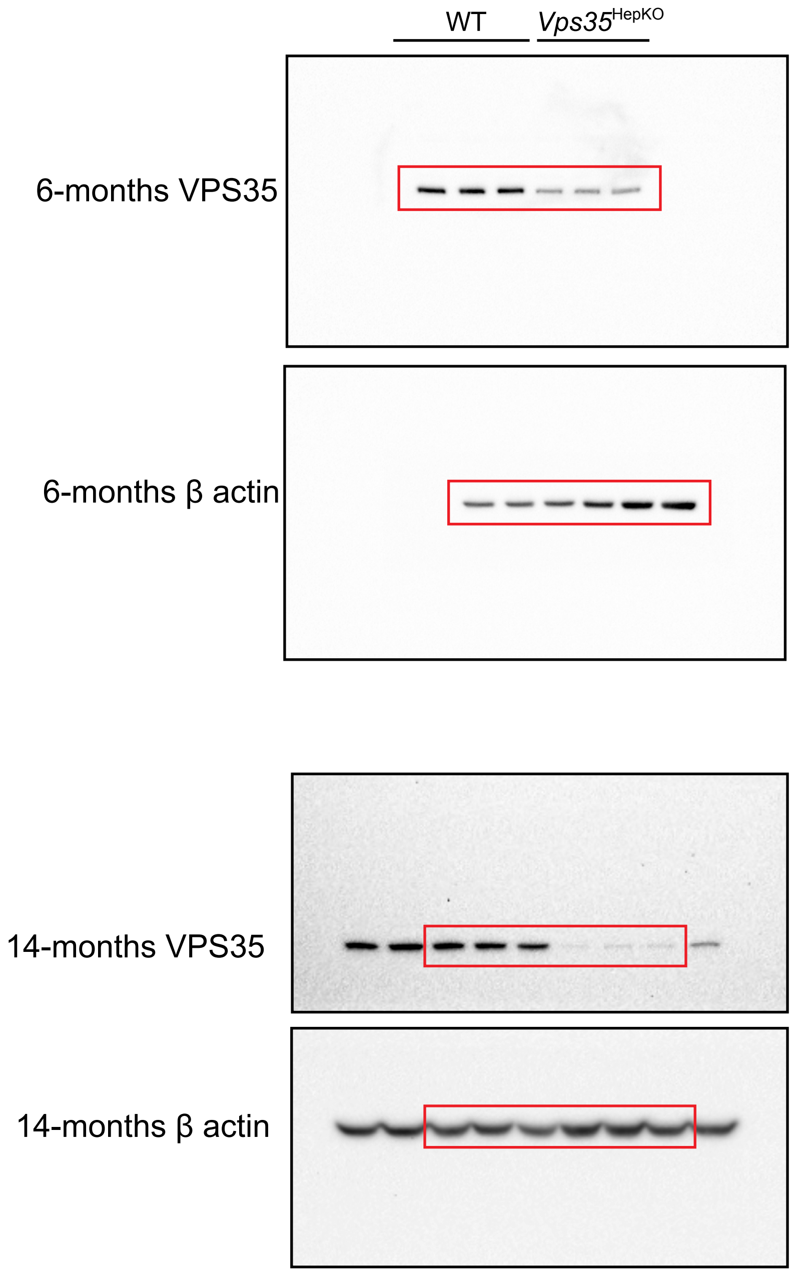

Supplemental Figure 5

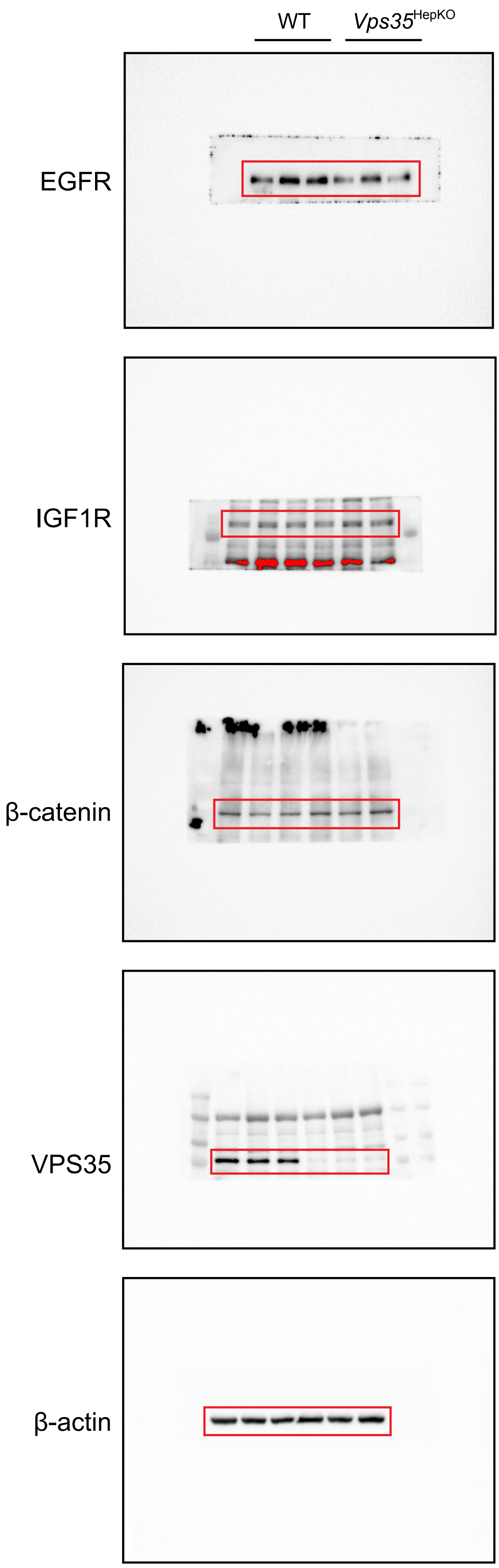

Supplemental Figure 7

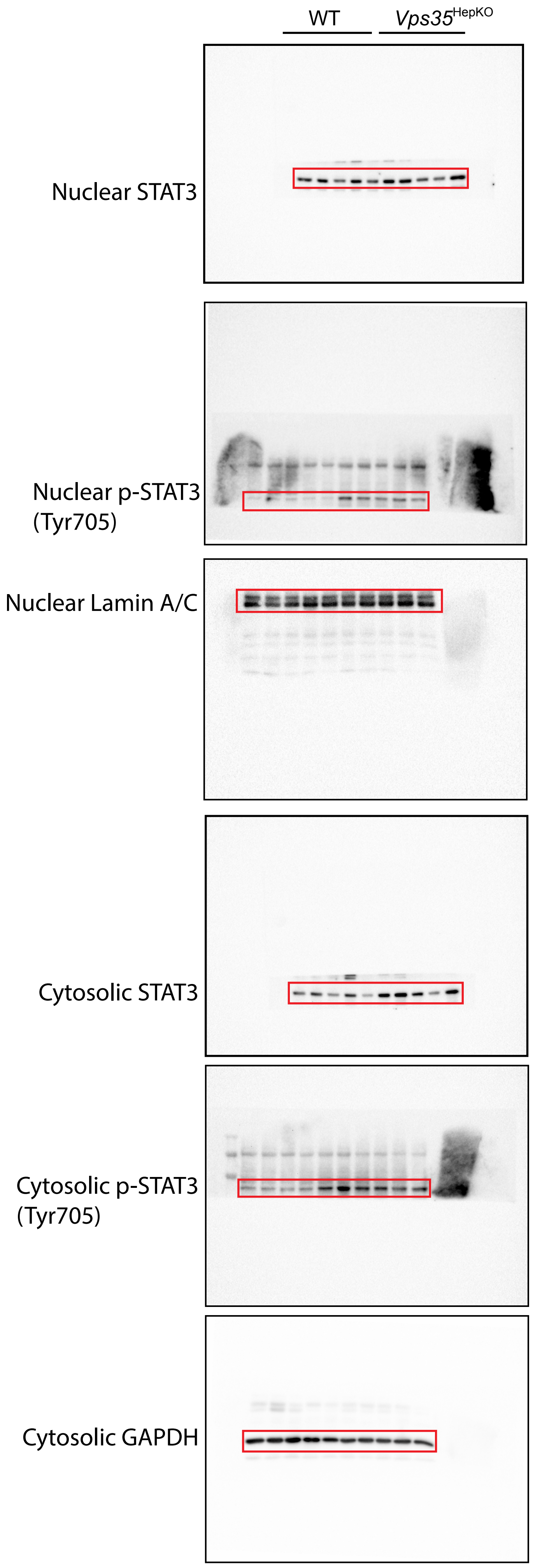

Supplemental Figure 8

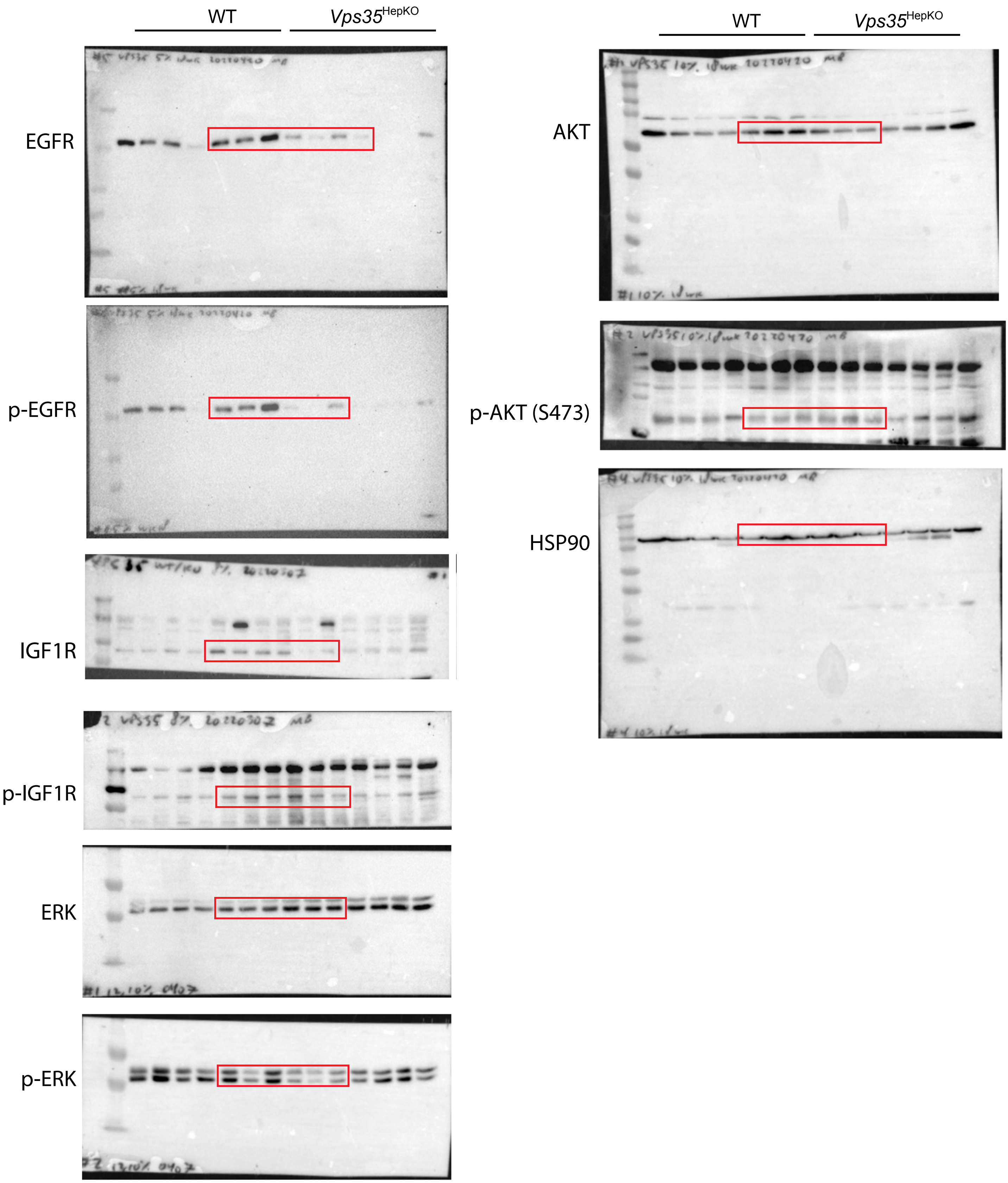

Supplemental Figure 9

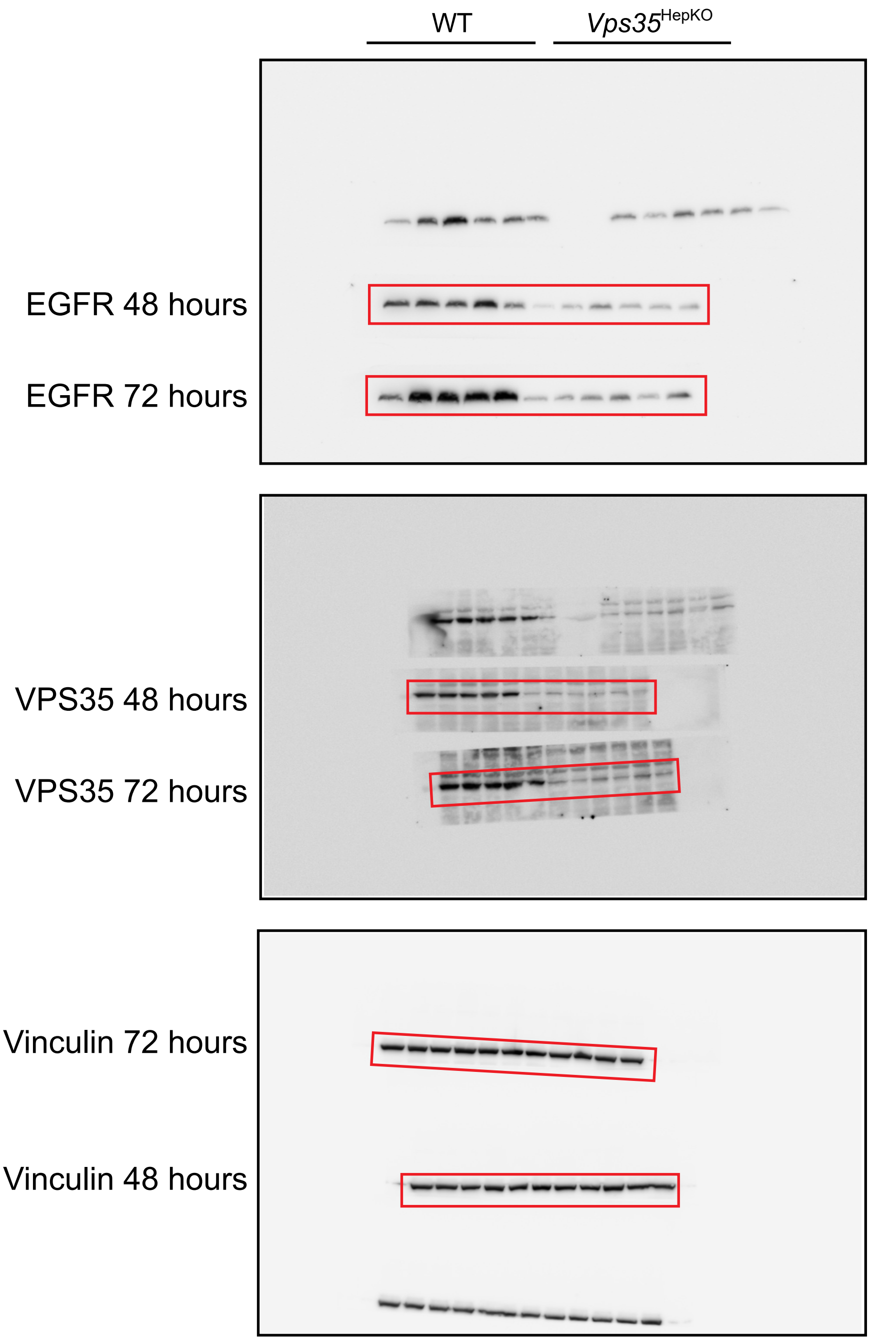

Supplemental Figure 12

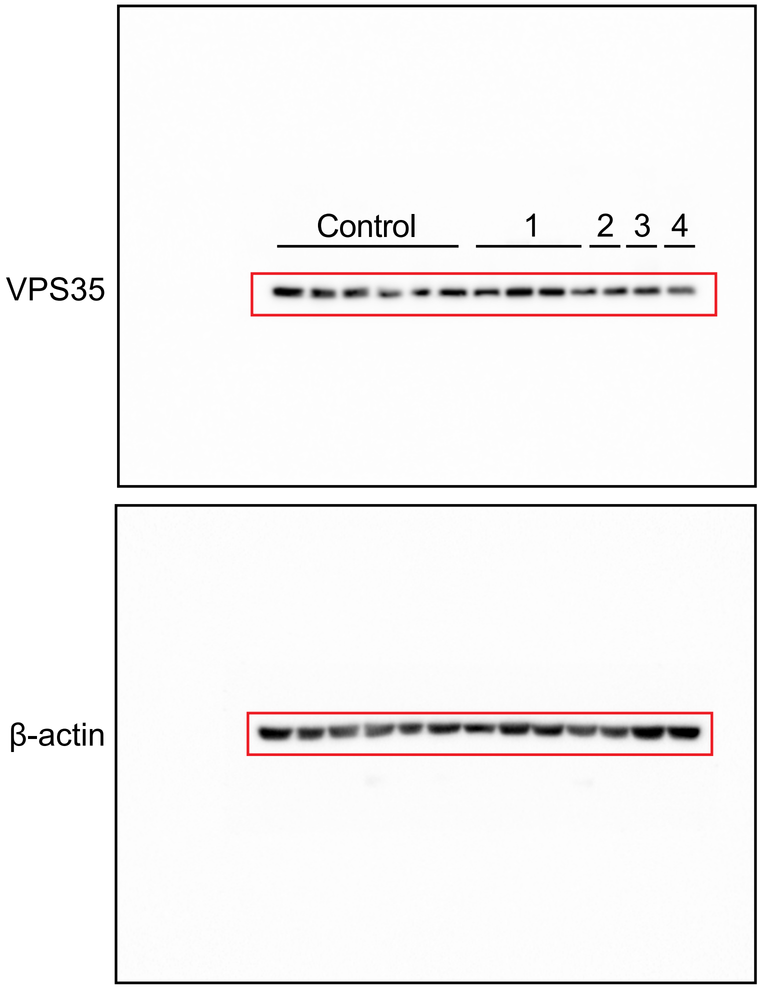

1: MASLD
2: MASH
3: MASH/Cirrhosis
4: Cirrhosis
