# Supplementary material for: Vacuolar Protein Sorting 35 Controls Hepatocellular Proliferation Through SRC Signaling and Promotes Diethyl Nitrosamine–Induced Tumor Initiation
Source: Cell Mol Gastroenterol Hepatol. 2026 May 5;20(8):101788. doi: 10.1016/j.jcmgh.2026.101788 (PMC13310651; doi:10.1016/j.jcmgh.2026.101788)

cmgh
CELLULAR AND MOLECULAR GASTROENTEROLOGY AND HEPATOLOGY

ORIGINAL RESEARCH

Vacuolar Protein Sorting 35 Controls Hepatocellular Proliferation Through SRC Signaling and Promotes Diethyl Nitrosamine–Induced Tumor Initiation

Markus G. Barbosa,[1] Dyonne Y. Vos,[1] Cristy R. C. Verzijl,[1] Andries H. Heida,[1] Marco Bakker,[1] Niels J. Kloosterhuis,[1] Mirjam H. Koster,[1] Marieke Smit,[1] Alba Fajardo,[3,4] Dicky Struik,[1] Peter Olinga,[2] Johan W. Jonker,[1] Rachel Thomas,[5] Albert Pol,[3,4,6] Vincent E. de Meijer,[7] Anna Moles,[8,9,10] Umesh Tharehalli,[1] Jan Albert Kuivenhoven,[1] and Bart van de Sluis[1]

[1]Department of Pediatrics, University of Groningen, University Medical Center Groningen, Groningen, the Netherlands; [2]Department of Pharmaceutical Technology and Biopharmacy, Groningen Research Institute of Pharmacy, University of Groningen, Groningen, the Netherlands; [3]Lipid Trafficking and Disease Group, Institut d'Investigacions Biomèdiques August Pi i Sunyer (IDIBAPS), Barcelona, Spain; [4]Department of Biomedical Sciences, Faculty of Medicine and Health Sciences, University of Barcelona, Barcelona, Spain; [5]Department of Biomolecular Health Sciences, Faculty of Veterinary Medicine, Utrecht University, Utrecht, the Netherlands; [6]Institució Catalana de Recerca i Estudis Avançats (ICREA), Barcelona, Spain; [7]Department of of Surgery, Section of Hepatobiliary Surgery and Liver Transplantation, University of Groningen, University Medical Center Groningen, Groningen, the Netherlands; [8]Institut d'Investigacions Biomèdiques August Pi I Sunyer (IDIBAPS), Barcelona, Spain; [9]Department of Experimental Pathology, Institute of Biomedical Research of Barcelona, Spanish National Research Council (CSIC), Barcelona, Spain; and [10]Centro de Investigación Biomédica en Red de Enfermedades Hepáticas y Digestivas (CIBERehd), Barcelona, Spain

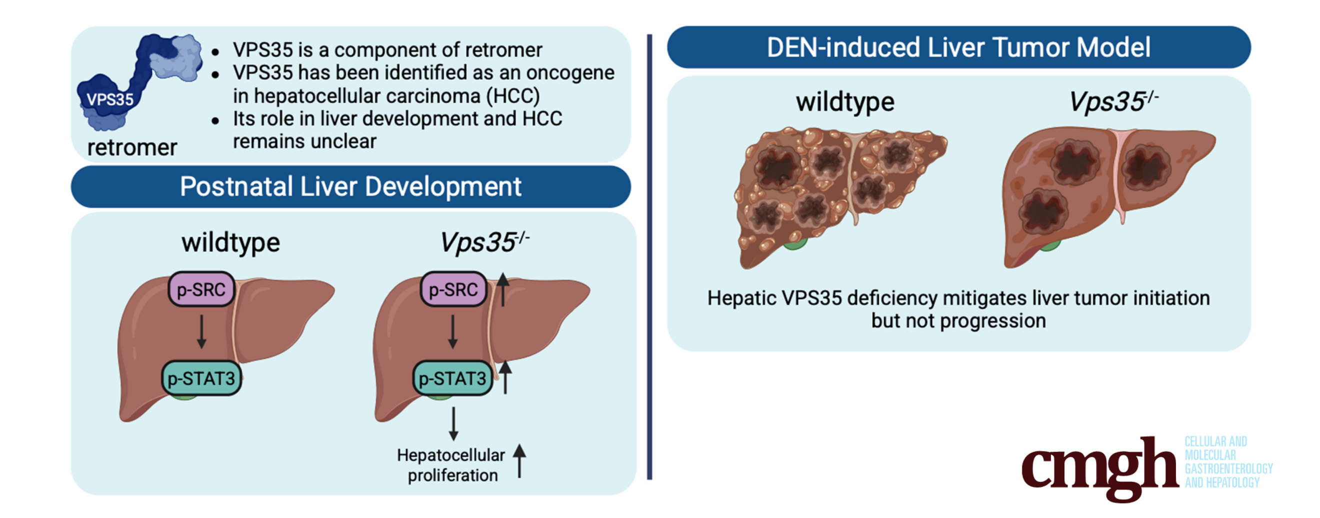

SUMMARY

Vacuolar protein sorting 35 has been dubbed an oncogene. Here, we found that vacuolar protein sorting 35 plays a crucial role in regulating hepatocellular proliferation in postnatal livers, and that hepatic vacuolar protein sorting 35 deficiency mitigates the formation of liver lesions, but not their growth, suggesting that vacuolar protein sorting 35 may not be a typical oncogene.

WHAT YOU NEED TO KNOW

Background: Vacuolar protein sorting 35 has been identified as a novel oncogene in hepatocellular carcinoma, but its true role in liver health remains unclear

Impact: Increasing our understanding of the mechanisms underlying hepatocellular carcinoma will open new avenues for better treatment options.

Future Directions: Although hepatic vacuolar protein sorting 35 deficiency attenuates the initiation of liver lesions, it remains unclear whether vacuolar protein sorting 35 is a target for hepatocellular carcinoma treatment, particularly given that vacuolar protein sorting 35 is essential for postnatal liver development.

BACKGROUND & AIMS: Hepatocellular carcinoma is the third leading cause of cancer-related mortality worldwide. Therapeutic options for hepatocellular carcinoma remain limited, and the mechanisms underlying hepatocellular carcinoma are not fully understood. Therefore, gaining a comprehensive understanding of the pathways that drive hepatocellular carcinoma is essential for improving treatments. Recent studies have identified vacuolar protein sorting 35, a component of the endosomal cargo sorting machinery called retromer, as a novel oncogene in various types of cancer, including hepatocellular carcinoma. However, its role in the initiation and progression of hepatocellular carcinoma is still unclear.

METHODS: To study the role of vacuolar protein sorting 35 in hepatocellular proliferation and the development of hepatocellular carcinoma, we generated a liver-specific *Vps35* knockout mouse model using the Cre-LoxP system ($Vps35^{HepKO}$). Hepatocellular proliferation was studied in young and middle-aged mice, as well as during liver regeneration after two-thirds partial hepatectomy. Diethyl nitrosamine was used to induce hepatocellular carcinoma. Livers were analyzed at histological, transcriptional, and proteomic levels.

RESULTS: Hepatic loss of vacuolar protein sorting 35 enhanced hepatocellular proliferation in post-natal livers via SRC and its downstream target signal transducer and activator of transcription 3. Pharmacologic inhibition of SRC with saracatinib normalized hepatocellular proliferation in $Vps35^{HepKO}$ mice. In contrast, hepatic vacuolar protein sorting 35 deficiency did not alter hepatocellular proliferation after partial hepatectomy in adult mice. Although vacuolar protein sorting 35-deficient postnatal livers exhibited an increased proliferative phenotype, hepatic loss of vacuolar protein sorting 35 reduced the number of diethyl nitrosamine–induced liver lesions without affecting tumor size.

CONCLUSIONS: Our in vivo data identify murine vacuolar protein sorting 35 as a critical regulator of hepatocellular proliferation in postnatal livers, but not after partial hepatectomy. Although vacuolar protein sorting 35 deficiency mitigates diethyl nitrosamine–induced liver lesion formation, it does not affect tumor progression, arguing against a role for vacuolar protein sorting 35 as a canonical oncogene. *(Cell Mol Gastroenterol Hepatol 2026;20:101788; https://doi.org/10.1016/j.jcmgh.2026.101788)*

Keywords: Endosomal Transport; Liver Cancer; Liver Development; Oncogene.

Hepatocellular carcinoma (HCC) is the most common type of liver cancer and is the third leading cause of cancer deaths worldwide.[1] Although alcohol abuse, hepatitis B and C are the main risk factors, the increasing prevalence of metabolic dysfunction-associated steatotic liver disease (MASLD) is changing the etiology and the prevalence of HCC in Western countries.[1,2] MASLD represents a spectrum of liver diseases characterized by hepatic lipid accumulation, known as simple steatosis.[3] Approximately 25% of these patients progress to a more severe condition known as metabolic dysfunction-associated steatohepatitis (MASH), characterized by liver damage and inflammation.[3,4] MASH can eventually result in liver fibrosis and ultimately progress toward cirrhosis and HCC.[3,4] It has been estimated that MASLD increases the risk of HCC by 7-fold,[5] with the highest incidence in patients with MASLD with cirrhosis.[5,6] However, HCC can also develop in patients with noncirrhotic MASLD.[5,7]

The development of HCC is associated with various genetic alterations, including activation of proto-oncogenes and loss-of-function mutations in tumor suppressor genes.[8] The most frequent genetic alterations in HCC are mutations in *TP53*, *CTNNB1*, and *ARID1A*.[1,8] However, the identification of these mutations has not yet resulted in successful therapies. HCC typically has a poor prognosis[9]; most patients with HCC are diagnosed in advanced stages of the disease, with a 5-year survival rate of less than 20%.[10] Only 25% of patients are eligible for potentially curative therapies, such as surgical resection and liver transplantation.[11] Systemic treatments such as the broad-spectrum tyrosine kinase inhibitor sorafenib, as well as immune checkpoint inhibitors and antiangiogenic drugs such as atezolizumab and bevacizumab, are recommended for patients with more advanced HCC.[12–14] Still, the long-term benefit is limited, mainly due to drug resistance.[8,13] To improve the diagnosis and treatment of patients with HCC, there is an urgent need to increase our understanding of the pathways involved in the initiation and progression of HCC.

The vacuolar protein sorting 35 (*VPS35*) gene has recently been identified as an oncogene in HCC and gastric cancer through in vitro and human association studies.[15,16] VPS35 forms, together with VPS26 and VPS29, the endosomal protein sorting complex retromer.[17] Retromer orchestrates the endosomal transport of a wide range of cell surface receptors, also known as 'cargos,' thereby regulating numerous cellular processes, including signaling pathways involved in cell proliferation.[18–21] Zhang and colleagues found that expression of *VPS35* was increased in

Abbreviations used in this paper: **BrdU, bromodeoxyuridine; BSA, bovine serum albumin; CYP, cytochrome P450; DAPI, 4′,6-diamidino-2-phenylindole; DEN, diethyl nitrosamine; DTT, dithiothreitol; E2F, E2 factor; ECM, extracellular matrix; EdU, 5-ethynyl-2′-deoxyuridine; EGF, epidermal growth factor; EGFR, epidermal growth factor receptor; ERK, extracellular signal-regulated kinase; FGFR3, fibroblast growth factor receptor 3; GSEA, gene set enrichment analysis; H&E, hematoxylin and eosin; HCA, hepatocellular adenoma; HCC, hepatocellular carcinoma; IGF1R, insulin-like growth factor-1 receptor; IGFR, insulin-like growth factor receptor; i.p., intraperitoneal; ITGA5, integrin alpha 5; MASLD, metabolic dysfunction-associated steatotic liver disease; MASH, metabolic dysfunction-associated steatohepatitis; mRNA, messenger RNA; PBS, phosphate-buffered saline; PH, partial hepatectomy; RT, room temperature; RTK, receptor tyrosine kinase; p-, phosphorylated; RNA-seq, RNA sequencing; RT-PCR, reverse transcription polymerase chain reaction; SFK, SRC kinase family; STAT3, signal transducer and activator of transcription 3; VPS35, vacuolar protein sorting 35; YAP, YES-associated protein.**

Most current article

© 2026 The Author(s). Published by Elsevier Inc. on behalf of American Gastroenterological Association Institute. This is an open access article under the CC BY license (http://creativecommons.org/licenses/by/4.0/).
2352-345X
https://doi.org/10.1016/j.jcmgh.2026.101788

different patient cohorts with HCC, and this increase in *VPS35* expression was significantly associated with poor survival.[15] Using human hepatoblastoma and HCC cell lines, like HepG2, MHCC-97H, SK-Hep1, Hep3B, and Huh7, and a xenograft tumor model, they demonstrated that VPS35 promotes PI3K-AKT signaling and accelerates the proliferation of hepatoma cells. According to the authors, this increase in PI3K-AKT signaling was mainly due to enhanced VPS35-mediated transport of the fibroblast growth factor receptor 3 (FGFR3) from endosomes back to the cell surface.[15] In another study, VPS35 was shown to promote cell proliferation in gastric tumors by enhancing endosomal recycling of the epidermal growth factor receptor (EGFR) and subsequent activation of the extracellular signal–regulated kinase (ERK)1/2 signaling pathway.[16,22] Interestingly, our recent study showed that hepatocyte-specific loss of VPS35 increased liver weight and elevated levels of the liver enzymes alanine aminotransferase and aspartate aminotransferase without hepatic steatosis or overt liver damage,[23] suggesting that VPS35 plays a crucial role in maintaining liver health.

Although the central role of retromer in maintaining cellular homeostasis has been extensively studied in cell lines,[18–21] the role of retromer in hepatocellular proliferation and liver cancer in vivo remains poorly defined. To fill this knowledge gap, we performed a detailed molecular characterization of a liver-specific VPS35-deficient mouse model. Additionally, we investigated the effect of VPS35 deficiency on hepatocellular proliferation after a two-thirds partial hepatectomy (PH). Finally, we used a diethyl nitrosamine (DEN)-induced HCC model to assess the contribution of VPS35 to the initiation and progression of liver cancer.

Results

Hepatocyte Vacuolar Protein Sorting 35 Deficiency Increases Hepatocellular Proliferation in Postnatal Mouse Livers, but Not in Livers of Middle-Aged Mice

Building on our previous finding that hepatic loss of VPS35 increases liver weight in 18-week-old male mice fed a standard chow diet and is accompanied by elevated alanine aminotransferase and aspartate aminotransferase levels,[23] we investigated whether this phenotype is associated with any liver pathology. Histologic characterization revealed that the hepatocytes are smaller in VPS35-deficient livers compared with controls (Figure 1*A*). Furthermore, *Vps35*^{HepKO} livers show an increased prevalence of mitotic figures (Figure 1*A*), indicating active cell proliferation. Indeed, the number of hepatocytes positive for proliferation marker Ki67 and bromodeoxyuridine (BrdU) incorporation was elevated after hepatic ablation of VPS35 (Figure 1*A* and *B*). To determine whether this enhanced proliferation also occurs in younger *Vps35*^{HepKO} mice, control and *Vps35*^{HepKO} male mice were sacrificed at 12 weeks of age. Despite no difference in liver-to-body weight ratio (Figure 2*A*), our findings are consistent with those in 18-week-old mice. Twelve-week-old VPS35-deficient livers show hyperplasia, marked by an increased number of smaller hepatocytes (Figure 2*B*). Additionally, we observed a significant increase in BrdU-positive hepatocytes in 12-week-old *Vps35*^{HepKO} mice compared with controls (Figure 2*B* and *C*). An increase in hepatocellular proliferation was also seen in 12-week-old female *Vps35*^{HepKO} mice, without affecting liver weight (Figure 3*A*–*E*).

To evaluate the longer-term effects of increased hepatocellular proliferation in mice lacking hepatic VPS35, we characterized control and *Vps35*^{HepKO} male mice at 6 and 14 months old, fed a standard chow diet. At these ages, the protein levels of VPS35 remained almost absent in *Vps35*^{HepKO} livers (Figure 4*A*). Body weight and liver weight were similar between control and *Vps35*^{HepKO} mice at 6 and 14 months of age (Figure 4*B* and *C*). The macroscopic appearance of the liver was similar between control and *Vps35*^{HepKO} livers at both ages (Figure 4*D*). In line, hematoxylin and eosin (H&E) staining from liver sections of both 6- and 14-month-old mice did not reveal abnormalities or evidence of the formation of lesions in *Vps35*^{HepKO} mice (Figure 1*C*). In contrast to our findings in young adult mice, the number of hepatic cells positive for the proliferative marker Ki67 in 6-month-old *Vps35*^{HepKO} mice and controls was similar (Figure 1*C* and *D*).

Taken together, these data indicate that hepatic loss of VPS35 increases hepatocellular proliferation in postnatal mouse livers, but this increase normalizes to levels seen in age-matched control mice.

Increased Hepatocellular Proliferation in Vacuolar Protein Sorting 35-Deficient Liver Is Not a Cell-Autonomous Effect nor Due to Increased Cell Death

It has previously been shown that increased programmed cell death in liver cells can result in compensatory hepatocellular proliferation.[24–26] However, we did not observe a marked increase in caspase 3-positive hepatocytes in VPS35-deficient livers, as determined by immunohistochemical characterization (Figure 5). These data support our previous findings, which showed that there was no increase in cell death after hepatic VPS35 ablation, as demonstrated with a terminal deoxynucleotidyl transferase dUTP nick end labeling assay.[23] Furthermore, senescent cells have been shown to promote the proliferation of neighboring cells through senescence-associated secretory phenotype in response to DNA damage and stress.[27] However, we observed no increase in p21-mediated cellular senescence in VPS35-deficient livers as determined by immunohistochemistry (Figure 5).

To determine whether the increased hepatocellular proliferation observed in *Vps35*^{HepKO} mice is a cell-autonomous effect of VPS35 deficiency, we performed an in vitro proliferation assay using primary hepatocytes from control and hepatic VPS35-deficient mice. 5-ethynyl-2′-deoxyuridine (EdU) immunofluorescent staining showed that the proliferation of primary hepatocytes from VPS35-deficient livers was reduced compared with that of

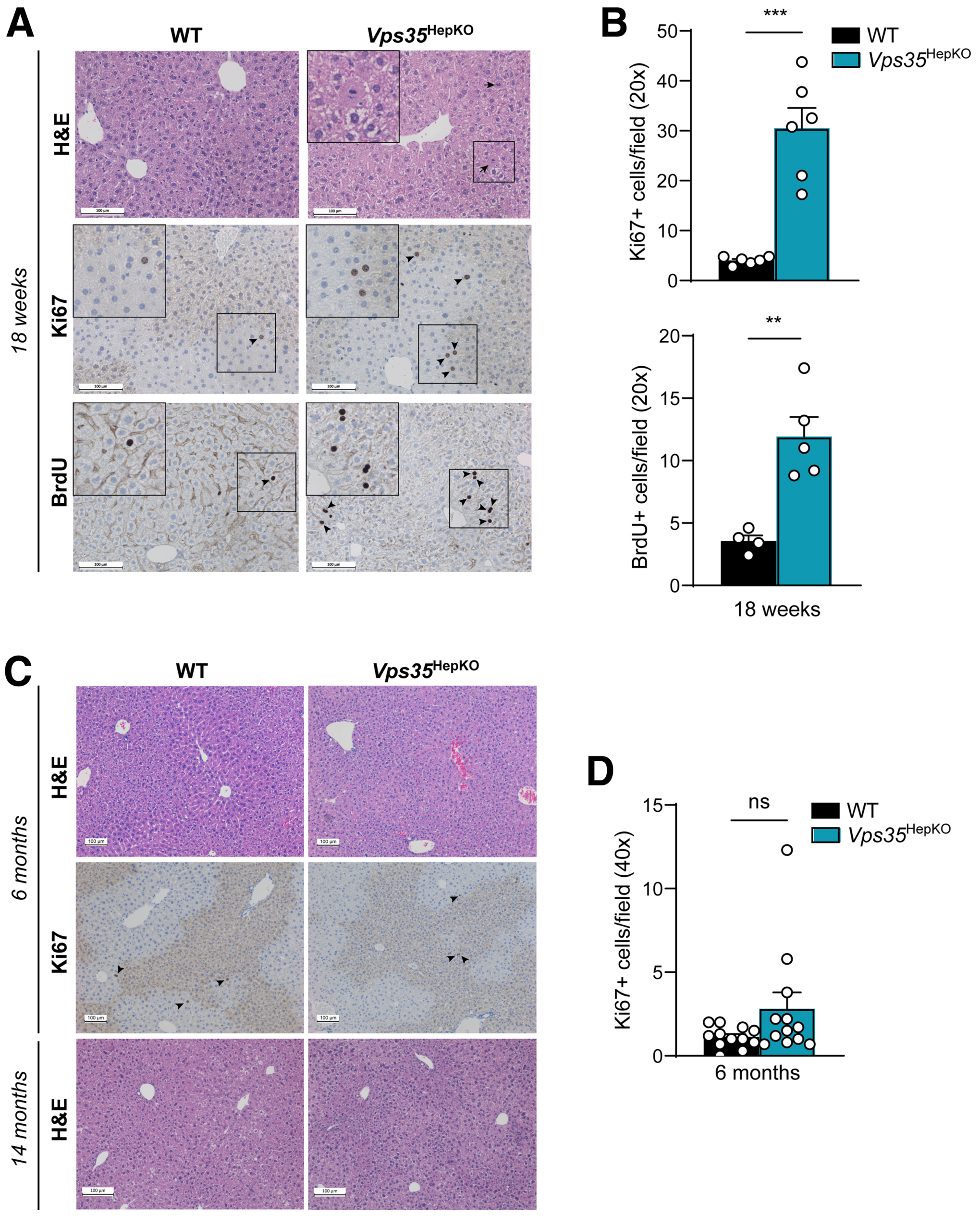

Figure 1. Hepatocyte-specific *Vps35* depletion increased hepatocellular proliferation in postnatal livers, but not in middle-aged mouse livers. (*A*) Representative images of H&E- and Brdu- and Ki67-stained liver sections from 18-week-old WT and *Vps35*^{HepKO} male mice; scale bars represent 100 μm, and *arrows* indicate mitotic figures in H&E, and positively stained cells for BrdU and Ki67 are indicated with *arrowheads*. (*B*) Quantification of BrdU$^+$ and Ki67$^+$ cells in (*A*). (*C*) Representative images of liver sections of WT and *Vps35*^{HepKO} male mice at 6 months of age, stained with H&E and for Ki67, and at 14 months of age, stained with H&E; scale bars represent 100 μm, and *arrowheads* indicate Ki67-positive hepatocytes. (*D*) Quantification of Ki67$^+$ cells in (*E*) (n = 12). Data are presented as mean ± SEM; **P < .01 and ***P < .001. An enlarged view of the area marked by a *square* is displayed in a corner of the image. SEM, standard error of the mean; WT, wild-type.

control mice upon epidermal growth factor (EGF) and hepatocyte growth factor stimulation (Figure 6*A* and *B*). It has been shown that VPS35 modulates cell proliferation through several pathways, including EGFR and the β-catenin signaling pathway.[16,19,28] To assess whether these pathways are affected in VPS35-deficient primary hepatocytes, and whether this could explain their reduced proliferative capacity, we examined the protein levels of EGFR and β-catenin. We also included the insulin-like growth factor-1 receptor (IGF1R), a member of the receptor tyrosine kinase (RTK) family that is critical for hepatocellular proliferation.[29] In line with previous studies, EGFR levels were significantly reduced in VPS35-deficient cells, whereas the levels of β-catenin and IGF1R were not affected (Figure 6*C* and *D*).

Altogether, our findings demonstrate that the increased hepatocellular proliferation in liver-specific VPS35-deficient mice is not due to a significant increase in cell death or cellular senescence, nor due to a cell-autonomous function of VPS35 in hepatocytes.

Increased Hepatocellular Proliferation Is Associated With Elevated Signal Transducer and Activator of Transcription 3 Activity in Vacuolar Protein Sorting 35-Deficient Livers

To gain insight into how hepatic loss of VPS35 increases hepatocellular proliferation, we performed RNA sequencing (RNA-seq) analysis. This revealed that 2387 genes were upregulated and 511 were downregulated in VPS35-

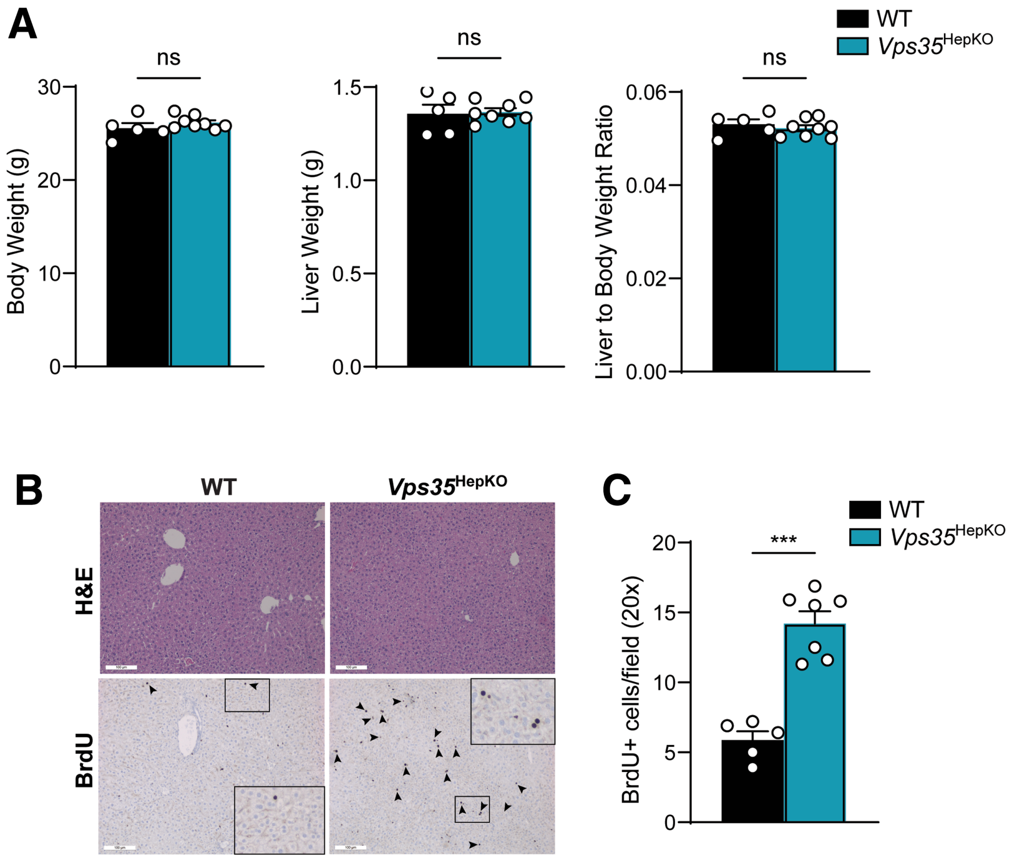

Figure 2. Hepatocyte-specific *Vps35* depletion increased hepatocellular proliferation in 12-week-old mice. (*A*) Body weight, liver weight, and liver-to-body weight ratio of 12-week-old WT and *Vps35*^{HepKO} male mice. (*B*) Representative images of H&E- and BrdU-stained liver sections from 12-week-old WT and *Vps35*^{HepKO} male mice; scale bars represent 100 μm, and *arrowheads* indicate positively stained cells. (*C*) Quantification of BrdU^{+} cells in (*B*) ($n = 5–7$). Data are presented as mean $\pm$ SEM; ***$P < .001$. SEM, standard error of the mean; WT, wild-type.

deficient livers compared with control livers (false discovery rate-adjusted *P* values < .05) (Figure 7*A*). Gene set enrichment analysis (GSEA) revealed pathways and hallmarks of the cell cycle and targets of the E2 factor (E2F) family of transcription factors, respectively (Figure 7*B*). E2F is essential for cell cycle regulation to control proliferation, survival, and development of postnatal and adult livers.[30–32] We also observed enrichment of gene sets related to cell cycle progression, such as the G2M checkpoint and mitotic spindle (Figure 8*A*).

Additionally, RNA-seq analysis found that the mechanosensitive transcriptional cofactor YES-associated protein (YAP)-target genes *Areg*, *Birc5*, *Itgβ2,* and *Sox9* were upregulated (Supplementary Table 1), a finding confirmed by quantitative reverse transcription polymerase chain reaction (RT-PCR) analysis (Figure 8*B*). YAP controls postnatal liver development by regulating the expression of genes involved in proliferation, survival, regeneration, and differentiation.[33] Although these genes were upregulated, the expression of the hepatocyte marker albumin was reduced in *Vps35*^{HepKO} mice compared with controls (Supplementary Table 1 and Figure 8*C*). Together, these findings support our immunohistochemical results showing that *Vps35*^{HepKO} livers exhibit aberrant cell cycle regulation and increased hepatocellular proliferation.

Further GSEA analysis of the transcriptomic data revealed an enrichment of the Janus kinase 2–signal transducer and activator of transcription 3 (STAT3) pathway in *Vps35*^{HepKO} livers (Figure 7*C*). The Janus kinase 2–STAT3 pathway is a major signaling pathway that controls a variety of cellular processes, including cell proliferation and differentiation.[34] Next, we evaluated whether these alterations in messenger RNA (mRNA) expression are associated with changes in STAT3 activation. Phosphorylation of STAT3 is followed by homodimerization and translocation to the nucleus, where it regulates the transcription of its target genes.[35] Therefore, we performed a nuclear extraction from liver samples of control and *Vps35*^{HepKO} mice and measured the protein levels of total and phosphorylated STAT3 (p-STAT3 Y705). We found that nuclear p-STAT3 levels were significantly higher in 12-week-old (Figure 7*D* and *E*) and 18-week-old (Figure 9*A* and *B*) *Vps35*^{HepKO} mice compared with control mice.

Taken together, these findings suggest that hepatic STAT3 activity is increased in liver-specific VPS35-deficient mice.

Kinase Activity of the Proto-Oncogene SRC Is Elevated in Vps35^{HepKO} *Mice*

STAT3 can be activated through various RTKs. Because VPS35 has also been linked to regulating the recycling of numerous RTKs, such as EGFR and FGFR3,[15,16] we aimed to identify its upstream kinases affected by VPS35 deficiency by conducting a kinome analysis using the PamGene

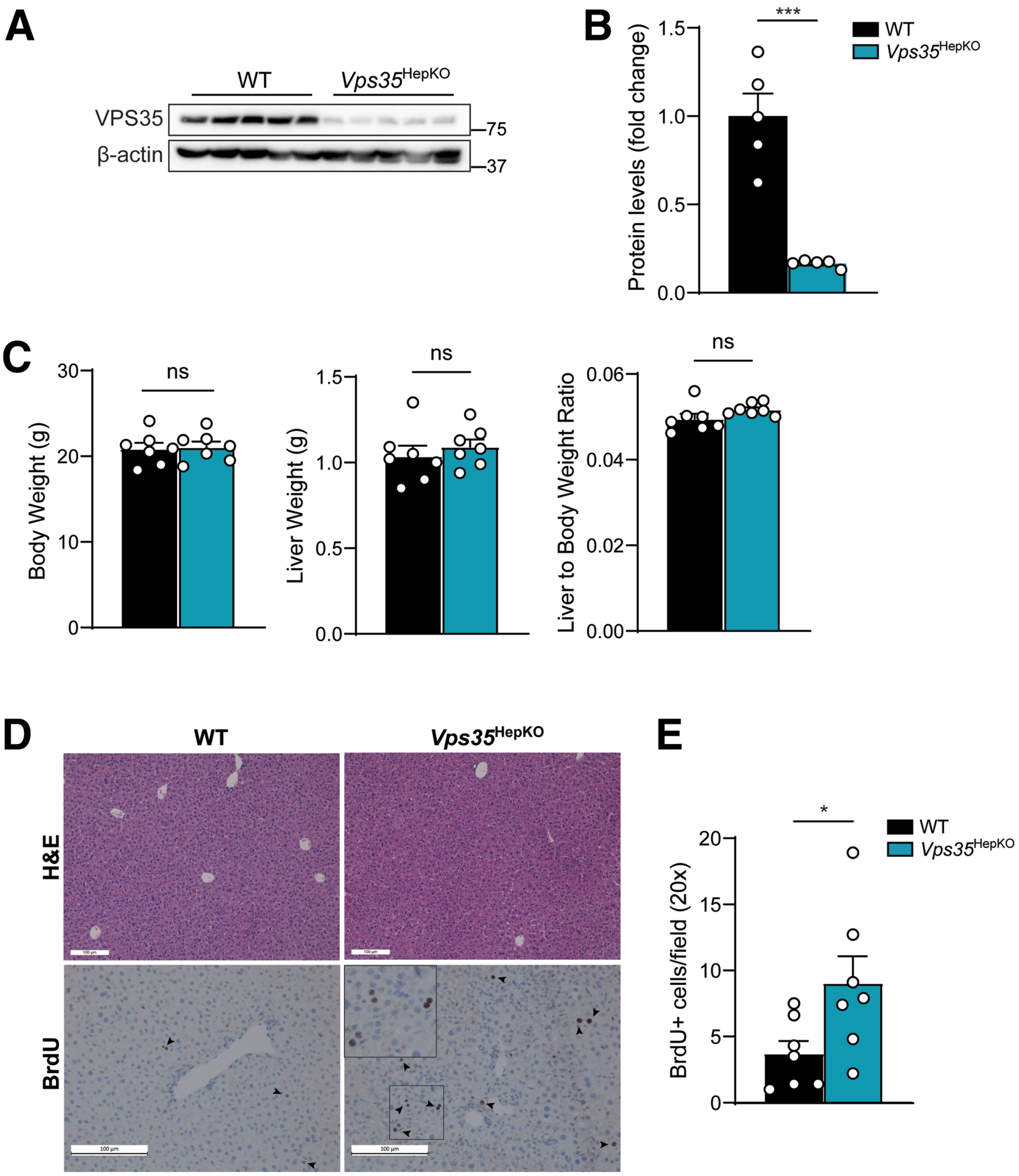

Figure 3. Characterization of 12-week-old WT and *Vps35*^{HepKO} female mice. (*A*) VPS35 protein levels in livers from 12-week-old WT and *Vps35*^{HepKO} female mice. (*B*) Quantification of VPS35 protein levels shown in (*A*) (n = 5). (*C*) Liver weight, body weight, and liver-to-body weight ratio of 12-week-old WT and *Vps35*^{HepKO} female mice. (*D*) Representative images of H&E- (10×) and BrdU- (20×) stained liver sections. BrdU-positive cells are indicated by *arrowheads*. An enlarged view of the area marked by a *square* is displayed in a corner of the image. (*E*) Quantification of BrdU^{+} hepatocytes (n = 7). Data are presented as mean ± SEM; **P* < .05 and ****P* < .001. SEM, standard error of the mean; WT, wild-type.

technology.[36] We found that the total tyrosine kinase activity was 82% higher in VPS35-deficient livers compared with controls (Figure 10*A*). Analysis of predicted upstream kinases revealed potential activation of the EGFR family (EGFR, ErbB2, ErbB3, ErbB4) and the insulin-like growth factor receptor (IGFR) family (IGF1R and INSR1) in VPS35-deficient livers (Figure 10*B*). To assess their involvement, we examined the total and phosphorylated protein levels of EGFR and IGFR1 and their downstream effectors. In VPS35-deficient livers, both total and phosphorylated EGFR levels were significantly decreased (Figure 11*A* and *B*). IGF1R levels showed a downward trend following hepatic VPS35 deletion, whereas p-IGF1R levels stayed the same (Figure 11*A* and *B*). Consequently, the p-IGF1R/ IGF1R ratio tended to increase in VPS35-deficient livers (Figure 11*A* and *B*). However, analysis of downstream kinases showed no increase in the activity of ERK or AKT pathways. In fact, p-ERK relative to total ERK was significantly reduced (Figure 11*A* and *C*). Collectively, these findings argue against the involvement of EGFR or IGF1R, along with their

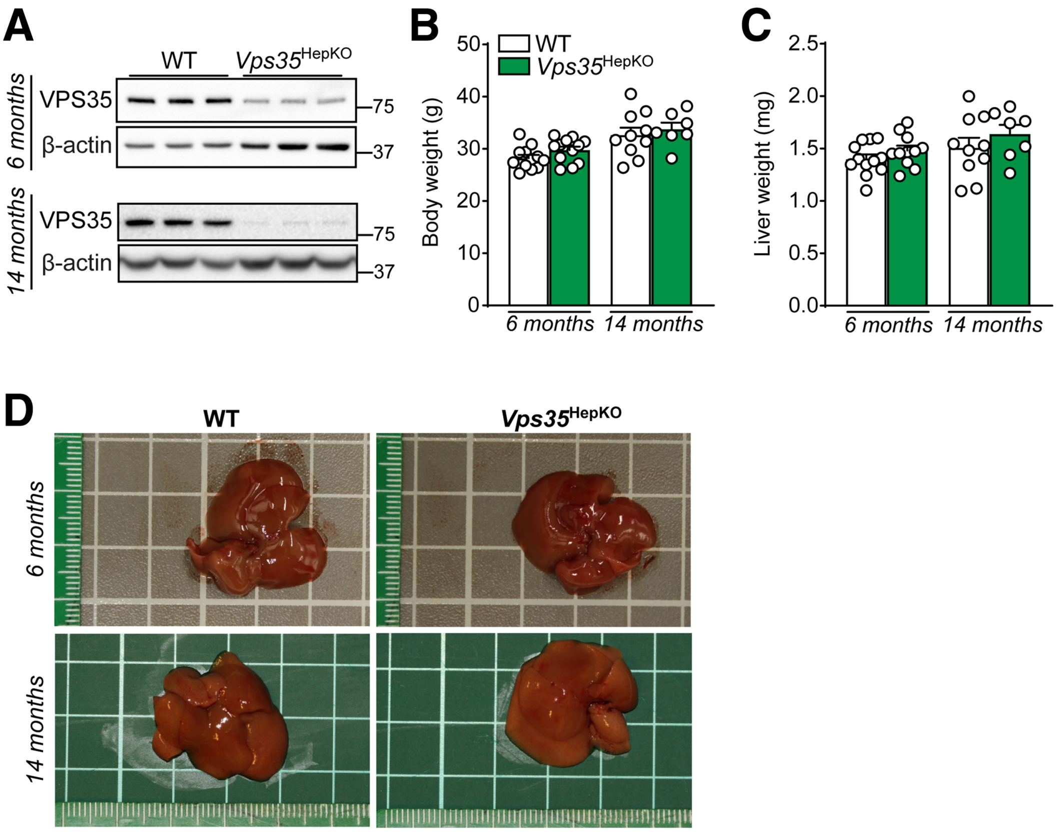

Figure 4. Characterization of *Vps35*^{HepKO} middle-aged mice. (*A*) Hepatic protein levels of VPS35 in 6-month and 14-month-old WT and Vps35^{HepKO} male mice. (*B*) Body weight and (*C*) liver weight of WT and Vps35^{HepKO} mice at 6 months (n = 11–12) and 14 months of age (n = 7–10). (*D*) Representative macroscopic images of control and Vps35^{HepKO} livers at 6 and 14 months of age. Grid squares are 1 × 1 cm. WT, wild-type.

downstream ERK and AKT signaling pathways, in mediating the increased hepatocellular proliferation in *Vps35*^{HepKO} mice.

Another kinase family predicted to be increased in VPS35-deficient livers was the SRC kinase family (SFK) (Figure 10*B*). SFK is a family of nonreceptor tyrosine kinases, including SRC, YES, FYN, FGR, LCK, HCK, and LYN, that can activate STAT3 through phosphorylation at tyrosine 705.[37,38] RNA-seq analysis (Supplementary Table 1) also identified SRC as the most prominently upregulated

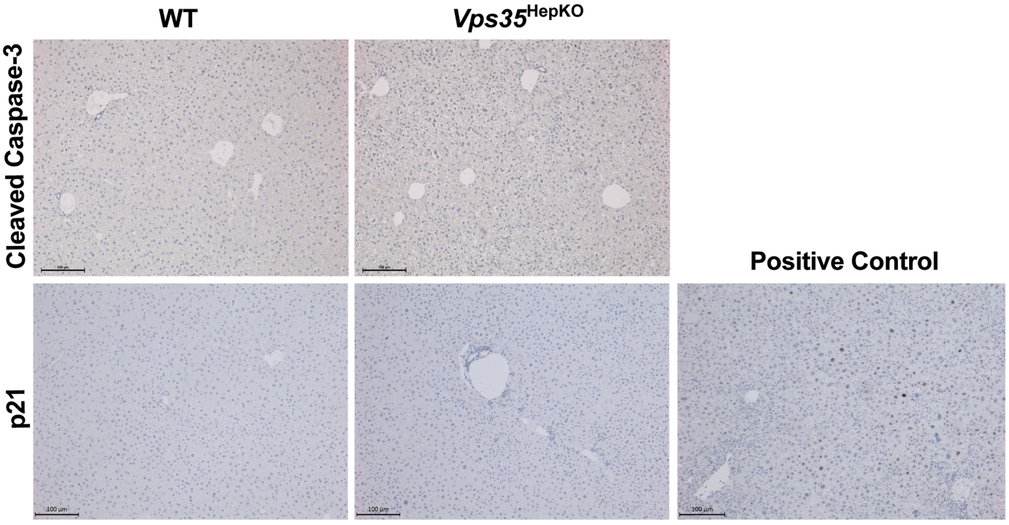

Figure 5. Hepatic loss of VPS35 is not associated with increased cell death or cellular senescence. Representative images of cleaved caspase-3 and p21 immunohistochemical staining of 18-week-old WT and VPS35-deficient male livers; scale bars represent 100 μm. WT, wild-type.

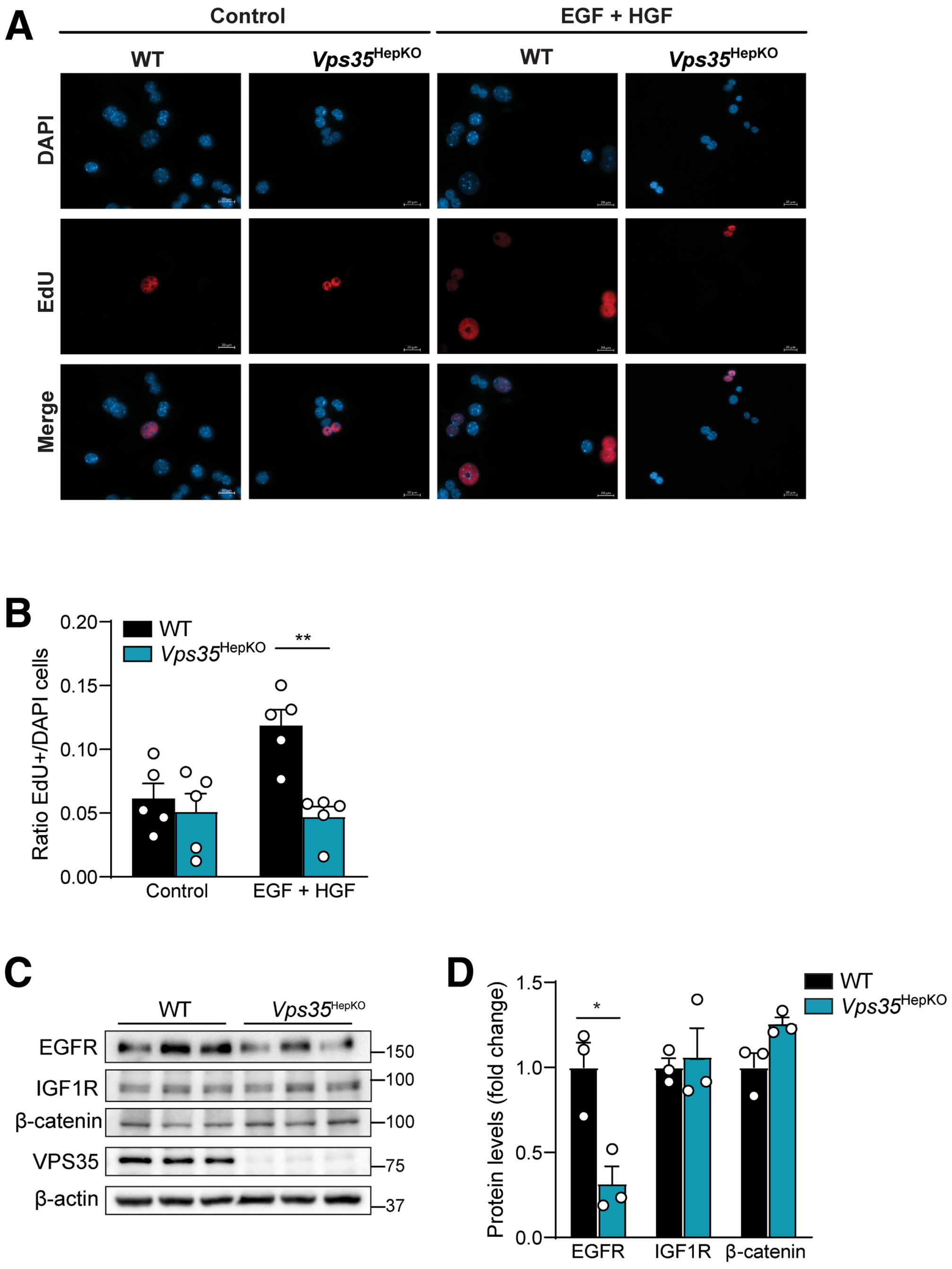

Figure 6. VPS35-deficient primary hepatocytes exhibit reduced proliferation upon EGF and HGF stimulation. (*A*) Representative images of EdU immunofluorescence imaging of WT and *Vps35*^{HepKO} primary hepatocytes cultured for 48 hours in culture medium (Control) and with the addition of EGF and HGF. DAPI (*blue*) and EdU (*red*); scale bars represent 20 μm. (*B*) Quantification of the ratio of EdU-positive cells to DAPI-positive cells (n = 5). (*C*) Protein levels of EGFR, IGF1R, and β-catenin in WT and *Vps35*^{HepKO} primary hepatocytes. (*D*) Quantification of protein levels shown in (C) (n = 3). Data are presented as mean $\pm$ SEM; $^{*}P < .05$ and $^{**}P < .01$. DAPI, 4′,6-diamidino-2-phenylindole; HGF, hepatocyte growth factor; WT, wild-type.

SFK member in VPS35-deficient liver. Therefore, we focused on SRC and examined total SRC protein levels, which were indeed elevated in *Vps35*^{HepKO} mice compared with controls (Figure 10*C* and *D*). In addition, we assessed phosphorylated SRC (p-SRC Y416) levels and found that p-SRC was significantly increased in VPS35-deficient livers, indicating enhanced SRC activation in the livers of *Vps35*^{HepKO} mice.

It has been shown that the extracellular matrix (ECM) plays an essential role in hepatocyte differentiation and proliferation.[39] Because retromer facilitates the intracellular trafficking of integrins, including integrin alpha 5 (ITGA5),[40,41] which can activate SRC signaling,[42,43] we assessed ITGA5 protein levels. We found that total ITGA5 levels were significantly increased in VPS35-deficient livers compared with controls (Figure 10*E* and *F*).

These data together reveal an association between elevated ITGA5 levels, increased SRC activity, and enhanced hepatocellular proliferation in VPS35-deficient livers.

Inhibition of SRC Reverses Increased Hepatocellular Proliferation in Vps35^{HepKO} *Mice*

To validate a possible role of SRC activity in the increased hepatocellular proliferation seen in VPS35-deficient livers, 11-week-old control and *Vps35*^{HepKO} mice

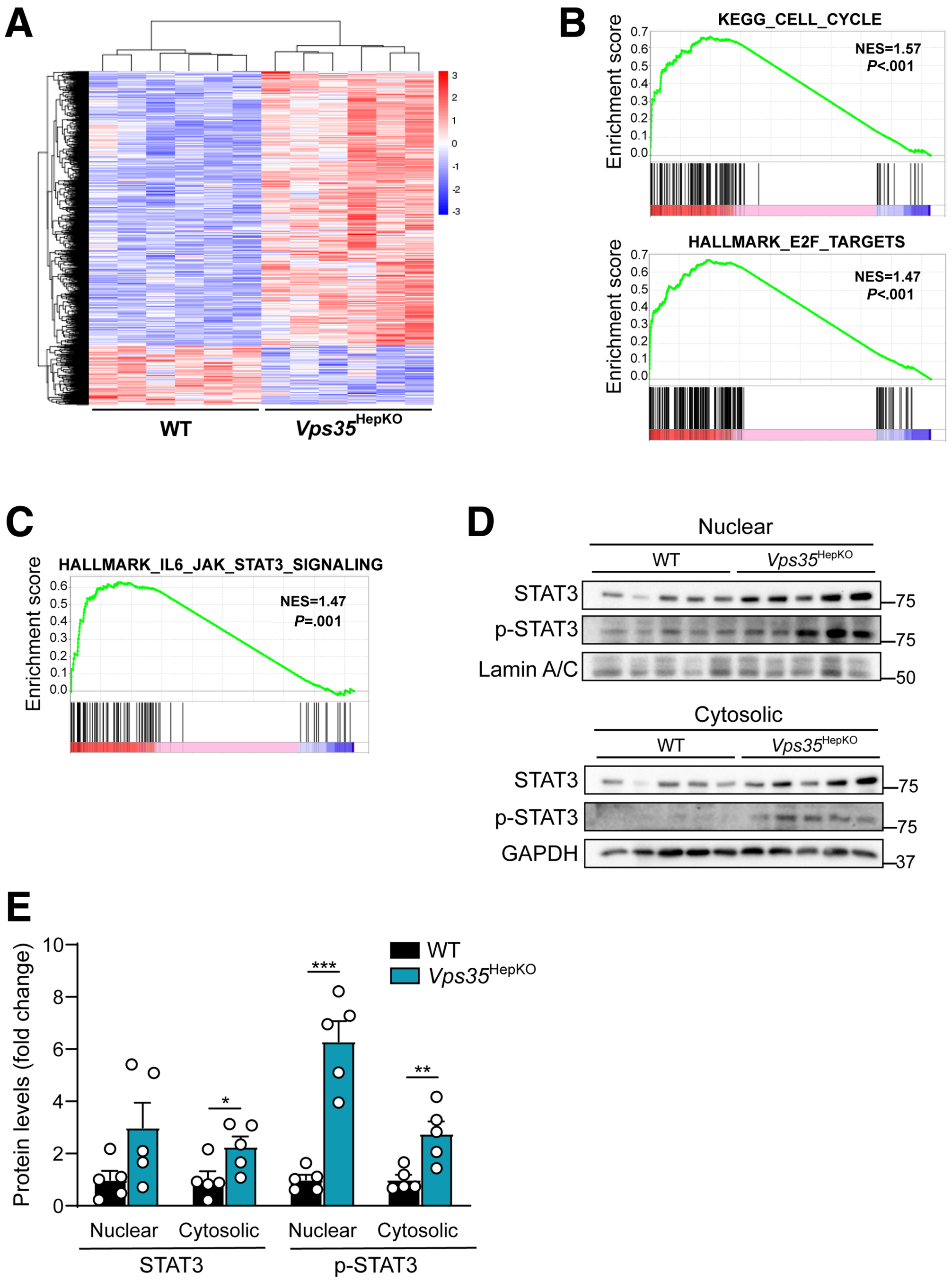

Figure 7. Hepatocytic loss of VPS35 is associated with proliferation pathways and increases STAT3 signaling. (*A*) Heatmap of differentially expressed genes in livers of WT and *Vps35*^{HepKO} 18-week-old male mice, determined by RNA-seq analysis (n = 6; FDR, 5%). (*B*) Enrichment of cell cycle and E2F target genes in *Vps35*^{HepKO} livers compared with WT livers, from GSEA analysis. (*C*) Enrichment of IL6-JAK-STAT3 signaling target genes in *Vps35*^{HepKO} livers compared to WT livers, from GSEA analysis. (*D*) Protein levels of STAT3 and p-STAT3 (Y705) in nuclear and cytosolic fractions of livers of WT and *Vps35*^{HepKO} 12-week-old male mice. (*E*) Quantification of Western blot shown in (*D*). Data are presented as mean ± SEM; **P* < .05, ***P* < .01, and ****P* < .001. FDR, false discovery rate; JAK, Janus kinase; NES, normalized enrichment score; SEM, standard error of the mean; WT, wild-type.

were treated with the selective SRC inhibitor, saracatinib (20 mg/kg),[44,45] for 7 consecutive days (Figure 12*A*). Saracatinib treatment significantly reduced hepatic p-SRC levels in *Vps35*^{HepKO} mice compared with vehicle-treated *Vps35*^{HepKO} mice (Figure 12*B* and *C*). In line, the number of BrdU-positive hepatocytes in *Vps35*^{HepKO} mice treated with saracatinib decreased to levels comparable to those observed in control mice (Figure 12*D* and *E*).

These findings suggest that elevated hepatocellular proliferation in *Vps35*^{HepKO} mice is mediated through SRC-dependent signaling.

Hepatic Loss of Vacuolar Protein Sorting 35 Does Affect Hepatocellular Proliferation After Partial Hepatectomy

Next, we performed two-thirds PH to further assess the role of VPS35 in hepatocellular proliferation in a physiologically relevant setting.[46] *Vps35*^{HepKO} mice and control littermates were sacrificed 24, 48, and 72 hours after PH. Both groups displayed a 100% survival rate (data not shown). As a measure of regenerative growth, we measured the liver-to-body weight ratio and found a significant reduction in *Vps35*^{HepKO} mice, with decreases of

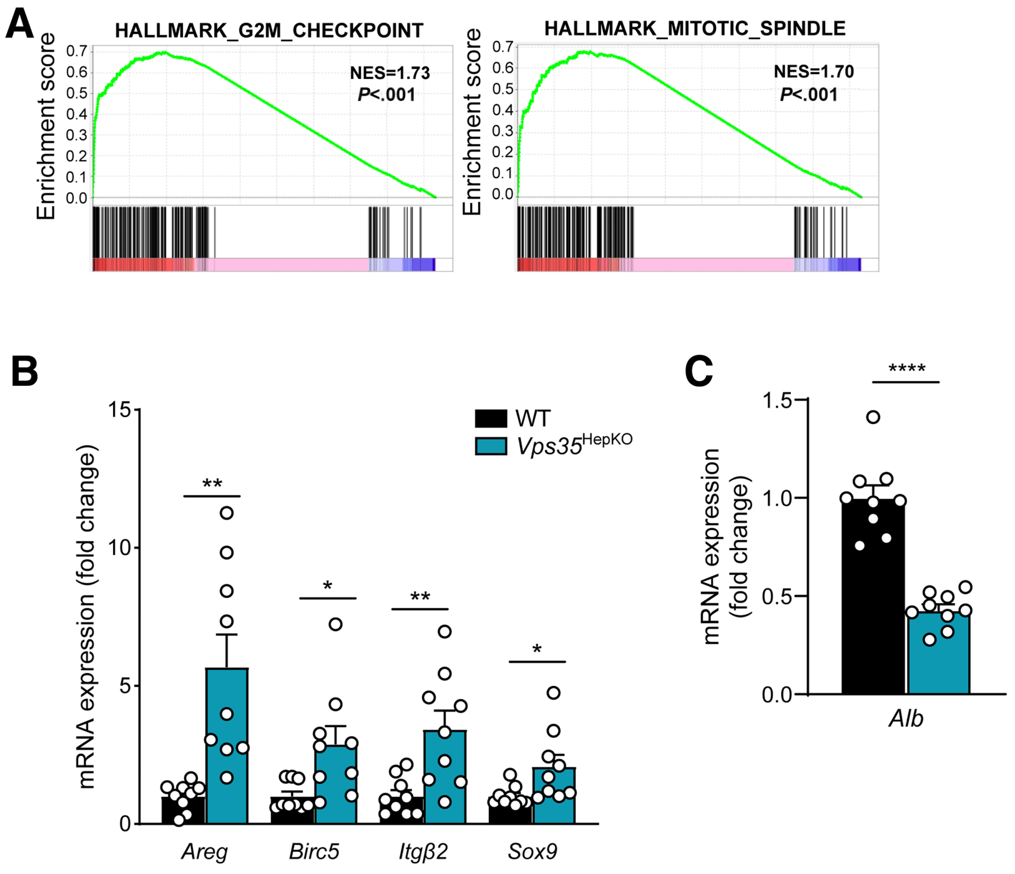

Figure 8. *Vps35*^{HepKO} livers exhibit aberrant cell cycle characterized by enrichment of G2/M phase markers, upregulation of mitotic spindle components, and increased expression of YAP target genes. (*A*) Enrichment of G2M checkpoint and mitotic spindle genes from GSEA analysis. (*B*) Relative hepatic mRNA expression of YAP-target genes, and (*C*) *Albumin* in 18-week-old WT and VPS35-deficient mice. Data are presented as mean ± SEM; *$P < .05$, **$P < .01$, and ****$P < .0001$. NES, normalized enrichment score; WT, wild-type.

7.48% and 7.62% at 48- and 72-hours post-PH, respectively (Figure 13*A*). Despite reductions in liver-to-body weight ratios in *Vps35*^{HepKO} mice, histologic analysis showed similar hepatocellular proliferation, with no differences in the number of mitotic bodies or the percentage of proliferation cell nuclear antigen–positive hepatocytes between VPS35-deficient and control livers (Figure 13*B*–*E*).

EGFR plays a key role in liver regeneration after PH[47] and because EGFR is one of the cargos of retromer,[16] we assessed the levels of EGFR in VPS35-deficient and control livers at 48 and 72 hours after PH. At all time points, we found that EGFR levels were reduced in *Vps35*^{HepKO} mice compared with controls. (Figure 14).

Collectively, in contrast to our results in postnatal VPS35-deficient livers, these data suggest that VPS35 does

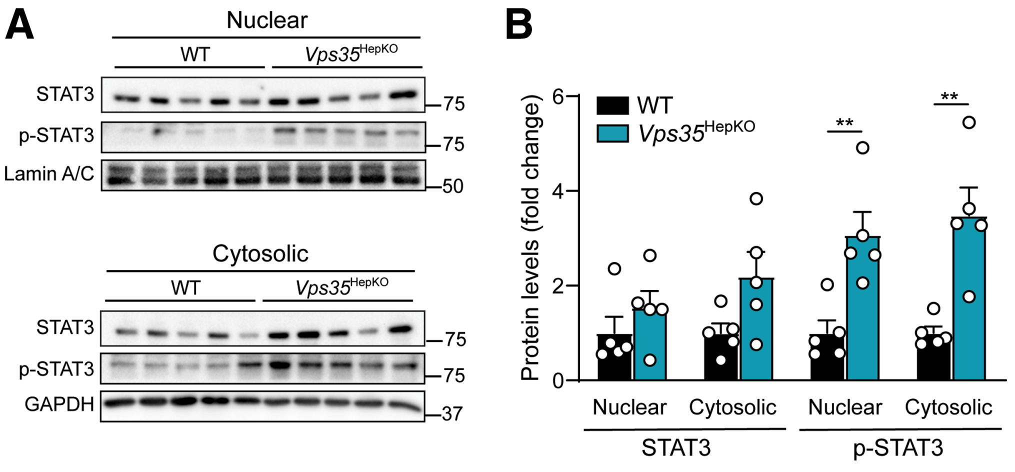

Figure 9. Hepatic loss of VPS35 increases STAT3 activity. (*A*) Protein levels of STAT3 and p-STAT3 (Y705) in nuclear and cytosolic fractions of livers of WT and *Vps35*^{HepKO} 18-week-old male mice. (*B*) Quantification of Western blot shown in (*A*) (n = 5). Data are presented as mean ± SEM; *$P < .05$ and **$P < .01$. SEM, standard error of the mean; WT, wild-type.

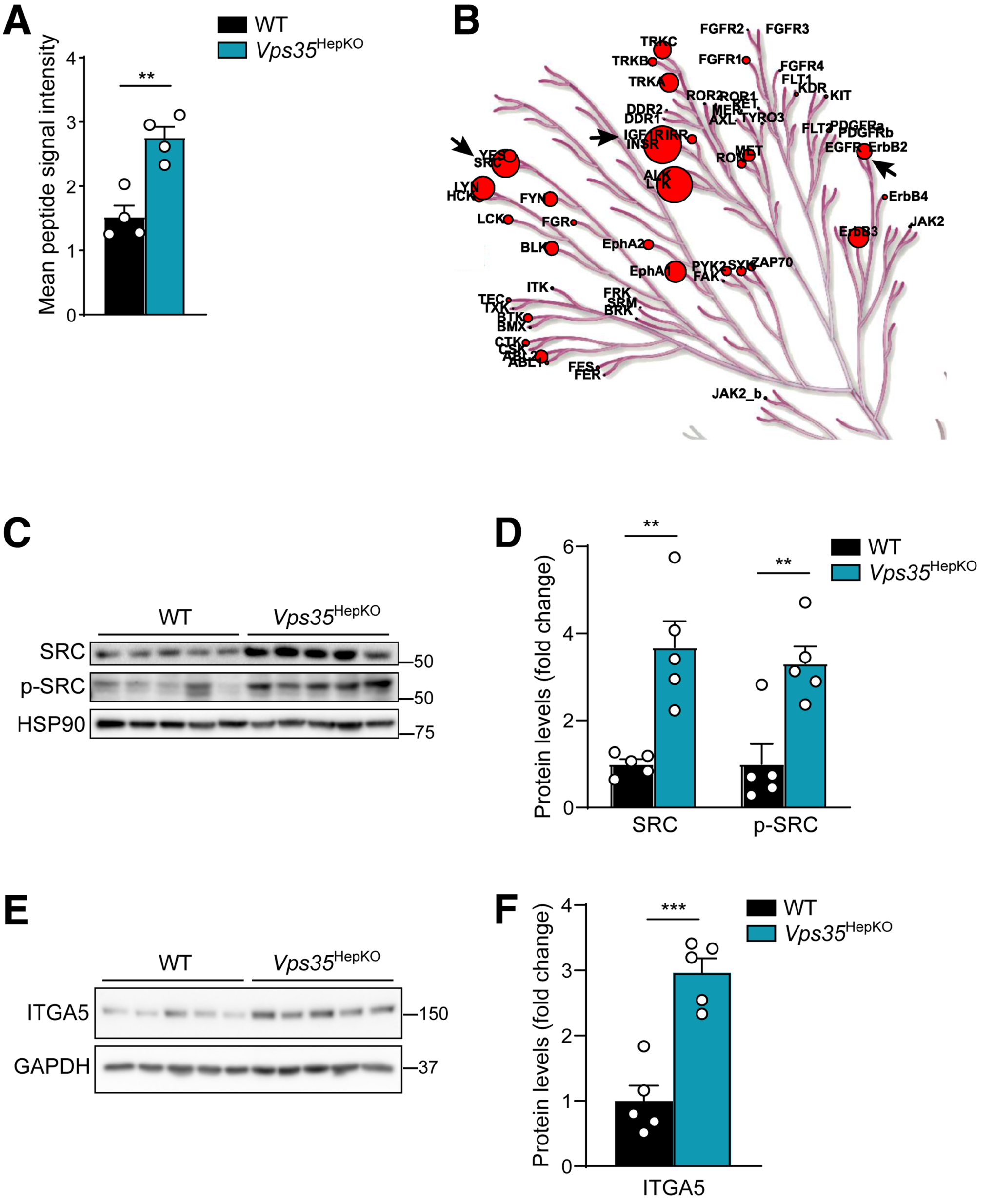

Figure 10. VPS35-deficient livers display enhanced activation of the proto-oncogene SRC. (*A*) Mean peptide signal intensity of *Vps35*^{HepKO} livers relative to WT livers (n = 4) as measured by PamGene's functional kinase assay. (*B*) A kinome tree of TKs with red dots showing upstream TKs that are predicted to be enriched in *Vps35*^{HepKO} livers compared with WT livers, based on the phosphorylation signal of peptide substrates (increasing *dot size* indicates higher significance). *Black arrows* indicate proto-oncogene tyrosine kinase SRC and RTKs, EGFR and IGF1R. (*C*) Total and phosphorylated protein levels of the proto-oncogene tyrosine kinase SRC in livers of 12-week-old WT and *Vps35*^{HepKO} male mice. (*D*) Quantification of protein levels shown in (*C*) (n = 5). (*E*) Protein levels of ITGA5 in livers of 12-week-old WT and *Vps35*^{HepKO} male mice. (*F*) Quantification of protein levels shown in (*E*) (n = 5). Data are presented as mean ± SEM; $^{**}P < .01$ and $^{***}P < .001$. SEM, standard error of the mean; TK, tyrosine kinases.

not play a key role in hepatocellular proliferation during liver regeneration after PH.

Hepatic Vacuolar Protein Sorting 35 Deficiency Mitigates Diethyl Nitrosamine–Induced Liver Cancer Initiation

Previous studies have identified VPS35 as an oncogene in various cancers, including HCC,[15] whereas the increased hepatocellular proliferation observed in postnatal VPS35-deficient livers might suggest that *VPS35* acts as a tumor suppressor gene. To evaluate the role of VPS35 in HCC development, we used a DEN-induced liver cancer model.[48] Two-week-old control and *Vps35*^{HepKO} mice were administered DEN (25 mg/kg, intraperitoneally [i.p.]) and analyzed at 32 and 40 weeks of age (Figure 15*A*). No significant differences were observed in body weight, liver weight, and liver-to-body weight ratio in either cohort (Figure 15*B*). At these timepoints, the protein expression of VPS35 in healthy liver tissue was still markedly reduced in *Vps35*^{HepKO} mice compared with control mice (Figure 16*A*). However, at both timepoints, *Vps35*^{HepKO} mice exhibited a significantly lower incidence of macroscopic liver lesions compared with controls (Figure 16*B* and *C*). Although the total lesion area was reduced in 40-week-old *Vps35*^{HepKO} mice, no difference was seen in 32-week-old mice (Figure 16*D*). When lesion size was normalized to the number of lesions, a modest, but not statistically significant, increase in average lesion size was observed in 40-week-old *Vps35*^{HepKO} mice (Figure 16*E*). However, this increase was not observed in 32-week-old mice (Figure 16*E*).

To determine whether the decreased number of liver lesions in *Vps35*^{HepKO} mice could be attributed to

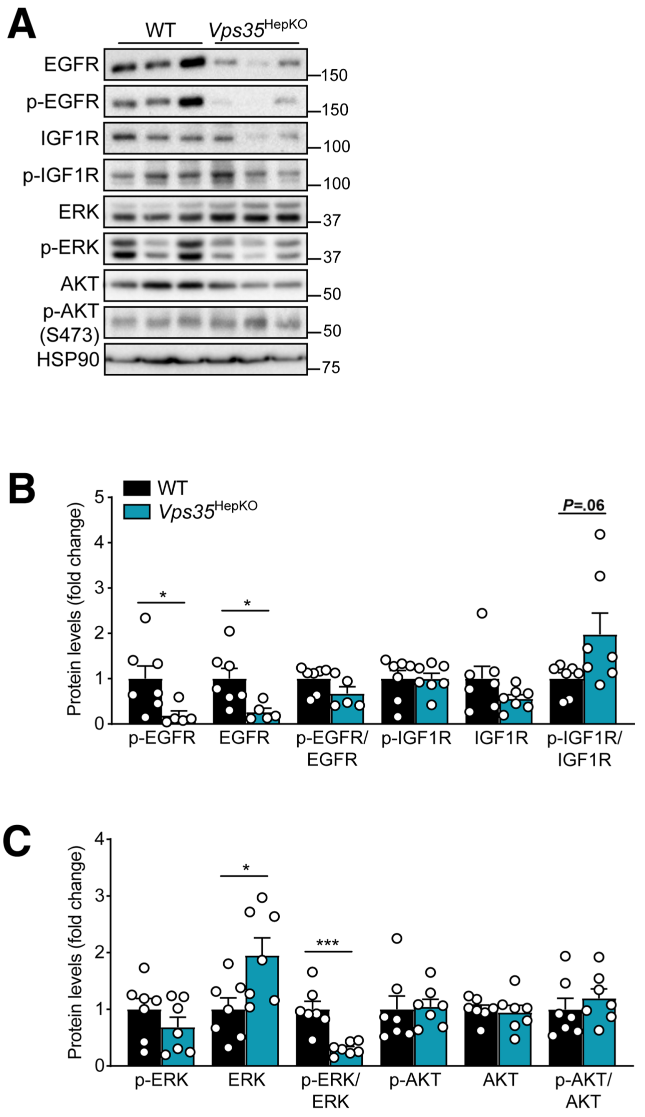

Figure 11. Total and phosphorylated protein levels of several kinases. (*A*) Total and phosphorylated protein levels of the receptor tyrosine kinases EGFR and IGFR, and downstream effectors ERK and AKT, in livers of WT and *Vps35*HepKO male mice. (*B* and *C*) Quantification of Western blot results shown in (*C*) (n = 5–7). Data are presented as mean ± SEM; **P* < .05 and ****P* < .001. SEM, standard error of the mean; WT, wild-type.

differences in DEN-induced DNA damage, we first examined the expression of cytochrome P450 (CYP) enzymes. These enzymes catalyze DEN into reactive intermediates capable of forming DNA adducts and initiating liver carcinogenesis.[49,50] Specifically, we assessed mRNA expression levels of *Cyp1a1, Cyp1a2,* and *Cyp2e1*, the primary CYP enzymes responsible for DEN metabolism.[49,50] At baseline (2 weeks of age) and 24 hours after DEN administration, expression levels of all 3 CYP genes were comparable between control and *Vps35*HepKO mice (Figure 17*A*). Second, we determined the effect of hepatic VPS35 deficiency on DEN-induced genotoxicity by monitoring hepatic DNA damage at 24 and 48 hours after DEN administration using gH2AX immunohistochemistry. The percentage of gH2AX-positive hepatocytes was similar between genotypes at both time points (Figure 17*B* and *C*), indicating comparable levels of DNA damage upon DEN administration. These findings suggest that the reduced number of liver lesions observed in *Vps35*HepKO mice compared with controls cannot be explained by differences in DEN-induced DNA damage at the time of carcinogen exposure.

Histopathologic analysis further supported the finding that hepatic VPS35 deficiency reduces the initiation of liver lesions, with a total of 86 lesions observed in 40-week-old *Vps35*HepKO mice compared with 129 in control mice (Figure 16*F*). The number of preneoplastic lesions and hepatocellular adenomas was reduced to 31 and 47, respectively, in *Vps35*HepKO mice compared with 52 and 71 in control mice (Figure 16*F*). Interestingly, the total number of HCCs was slightly higher in *Vps35*HepKO mice (8 vs 6 in controls) (Figure 16*F*). When expressed as a proportion of total lesions, HCC prevalence in *Vps35*HepKO livers was doubled (9.3% vs 4.6% in controls) (Figure 16*G*). Although a previous study[15] showed that VPS35 protein expression can be increased in HCC, we found no differences in VPS35 levels between healthy liver tissue and DEN-included liver lesions in control mice (Figure 16*H*).

Collectively, our findings indicate that VPS35 promotes lesion initiation in the DEN-induced HCC model. However, loss of VPS35 does not reduce lesion size or the number of malignant tumors; instead, the average size per lesion and the relative proportion of HCCs were slightly increased.

VPS35 Messenger RNA Levels Are Elevated in the Liver of Patients With Metabolic Dysfunction-Associated Steatohepatitis and Liver Cirrhosis

Because VPS35 modulates HCC initiation, we next assessed *VPS35 mRNA expression* in liver pathologies predisposed to liver cancer, including MASLD, MASH, and cirrhosis. The recently published MegaMASLD database,[51] which integrates multiple publicly available human liver transcriptomic datasets from healthy individuals and patients across the MASLD spectrum, was obtained from the Gene Expression Omnibus. Analysis revealed that *VPS35* mRNA expression increases with MASLD severity. *VPS35* is significantly upregulated in MASH and cirrhosis compared with both control and MASLD, whereas the difference between MASLD and control was not significant (Figure 18*A*). Consistently, negative matrix factor–based transcriptomic risk stratification within MegaMASLD showed significantly higher *VPS35* expression in the high-risk progressive MASLD group compared with the low-risk group (Figure 18*B*). Despite significant differences in *VPS35* mRNA levels between MASH and cirrhosis, we did not observe changes in VPS35 protein levels in livers from patients with MASLD, MASH, or liver cirrhosis (Figure 18*C*).

These findings indicate that *VPS35* mRNA levels are significantly increased in MASH and liver cirrhosis, without a clear effect on VPS35 protein levels.

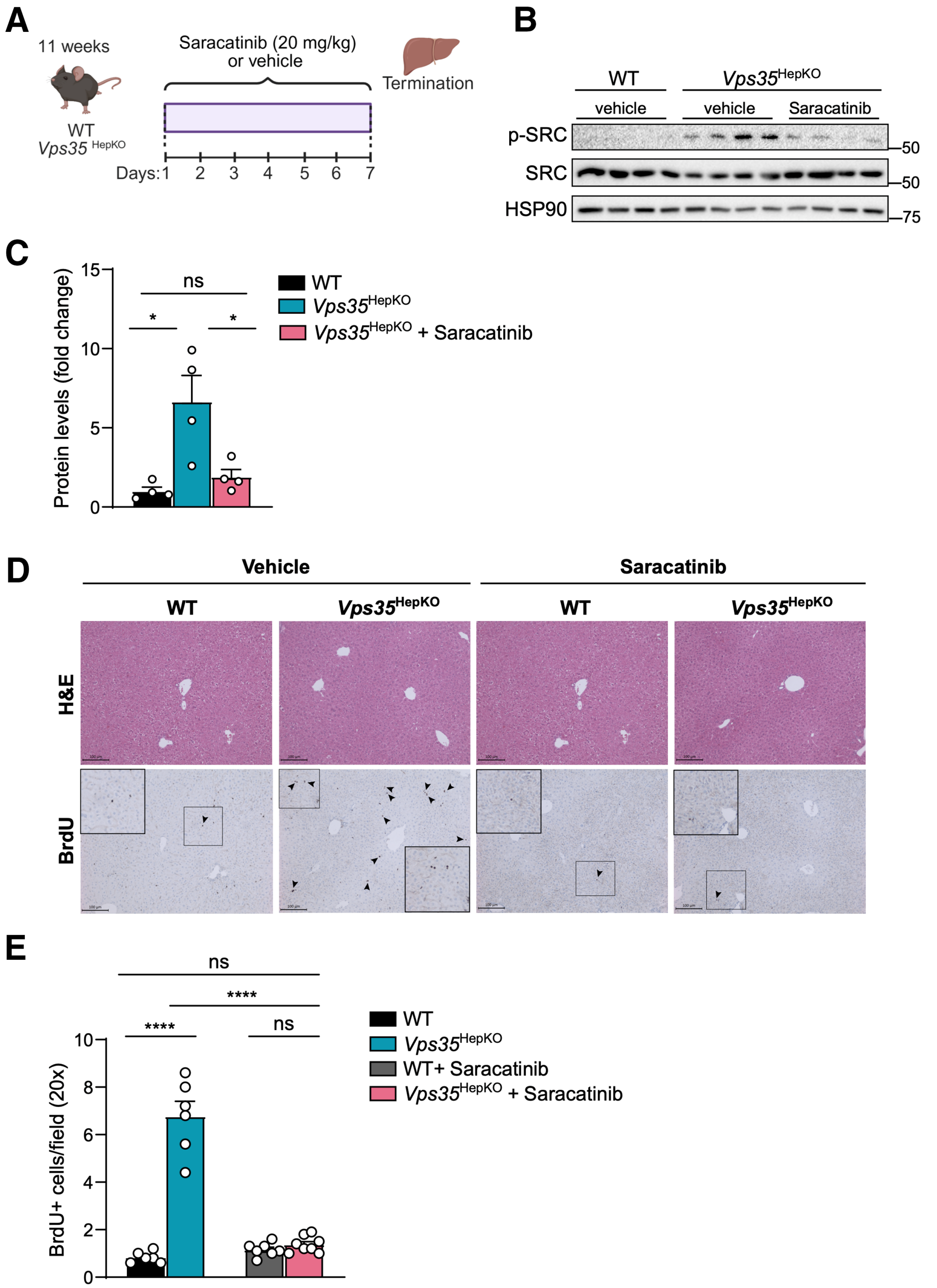

Figure 12. Pharmacologic inhibition of the SRC signaling pathways normalizes hepatocellular proliferation in VPS35-deficient liver. (*A*) Experimental design: 11-week-old WT and *Vps35*^{HepKO} male mice were injected with saracatinib (20 mg/kg) for 7 consecutive days. (*B*) Protein levels of total and phosphorylated proto-oncogene tyrosine kinase SRC in the liver of vehicle or saracatinib (20 mg/kg)-treated WT and *Vps35*^{HepKO} male mice. (*C*) Quantification of p-SRC (Y416) Western blot shown in (*B*) (n = 4). (*D*) Representative images of H&E- and BrdU-stained liver sections from vehicle or saracatinib (20 mg/kg) treated WT and *Vps35*^{HepKO} male mice; scale bars represent 100μm, and *arrowheads* indicate BrdU-positive hepatocytes. An enlarged view of the area marked by a *square* is displayed in a corner of the image. (*E*) Quantification of BrdU^{+} cells in D (n = 6–8). Data are presented as mean ± SEM; $^{*}P < .05$ and $^{****}P < .0001$. SEM, standard error of the mean; WT, wild-type.

Discussion

Recent studies have identified *VPS35* as an oncogene in HCC and demonstrated that VPS35 promotes proliferation of hepatoma cells via the FGFR3-PI3K/AKT axis.[15] These findings were derived from experiments using continuously cultured hepatoblastoma and HCC cell lines, but the role of VPS35 in hepatocellular proliferation and liver tumorigenesis in vivo has remained unclear. To this end, we generated a liver-specific *Vps35* knockout mouse model to examine the effect of hepatic VPS35 deficiency on hepatocellular proliferation and the development of liver lesions, both in the presence and absence of DEN treatment. In line with prior in vitro work,[15] VPS35 deficiency reduced the proliferation of cultured primary hepatocytes. In contrast, hepatocytic loss of VPS35 increased hepatocellular proliferation in relatively young mice, likely via activation of the SRC signaling pathway. However, during liver regeneration after PH, hepatic VPS35 deficiency does not affect hepatocellular proliferation, whereas its loss reduces the incidence of DEN-induced liver lesions. Strikingly,

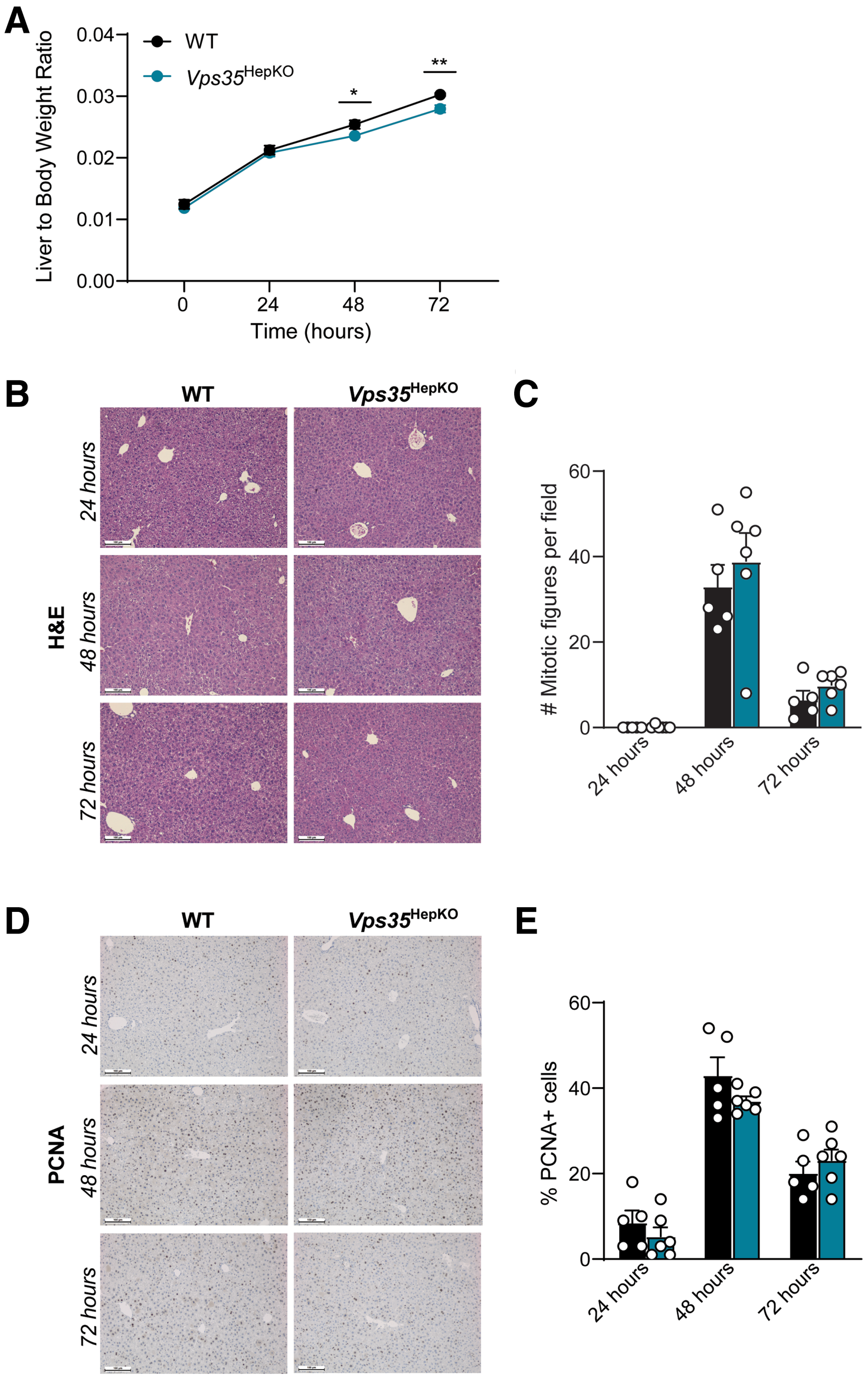

Figure 13. Hepatocellular proliferation after two-thirds PH is not affected in *Vps35*HepKO mice. (*A*) Liver-to-body weight ratio at 24-, 48-, and 72-hours post two-thirds PH. (*B*) Representative images of H&E-stained liver sections from WT and *Vps35*HepKO male mice; scale bars represent 100μm. (*C*) Quantification of mitotic figures (n = 5–6) in 20× screens on digital slides. (*D*) Representative images of PCNA-stained liver sections from WT and *Vps35*HepKO male mice; scale bars represent 100μm. (*E*) Quantification of the percentage of PCNA^{+} cells in (*D*) (n = 5–6). Data are presented as mean ± SEM; **P* < .05 and ***P* < .01. PCNA, proliferation cell nuclear antigen; WT, wild-type.

VPS35 deficiency tends to promote tumor growth and malignancy in this preclinical mouse model. Taken together, these findings suggest that VPS35 does not function as a typical oncogene in murine liver cancer.

The contrasting effects of VPS35 deficiency on hepatocellular proliferation in postnatal mouse liver tissue vs isolated hepatocytes and hepatoma/HCC cell lines suggest that the cellular microenvironment and intercellular

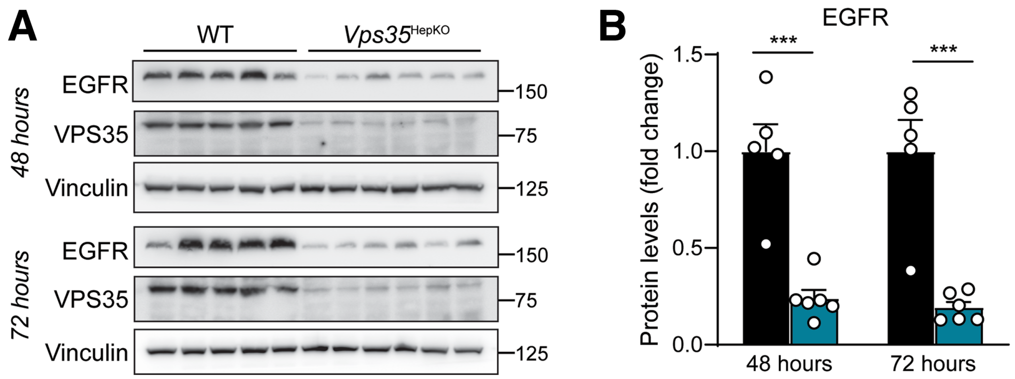

Figure 14. Hepatic EGFR and VPS35 levels in mice following two-thirds PH. (*A*) Protein levels of EGFR and VPS35 in the livers of WT and *Vps35*^HepKO^ male mice 48 and 72 hours post two-thirds PH. (*B*) Quantification of Western blots results shown in (*A*) (n = 5–6). Data are presented as mean ± SEM; ****P* < .001. SEM, standard error of the mean; WT, wild-type.

signaling pathways strongly influence the role of VPS35 in hepatocytes. Likely, in vitro experiments illustrate the cell-autonomous function of VPS35, whereas at an organismal level, the effect of VPS35 deficiency on hepatocyte function relies also on the hepatic microenvironment, in which hepatocytes are polarized and interact with other cells within the liver, such as nonparenchymal cells (eg, stellate and epithelial cells) and immune cells like Kupffer cells.[52,53] These interactions are essential for normal liver physiology, such as during postnatal liver development. After

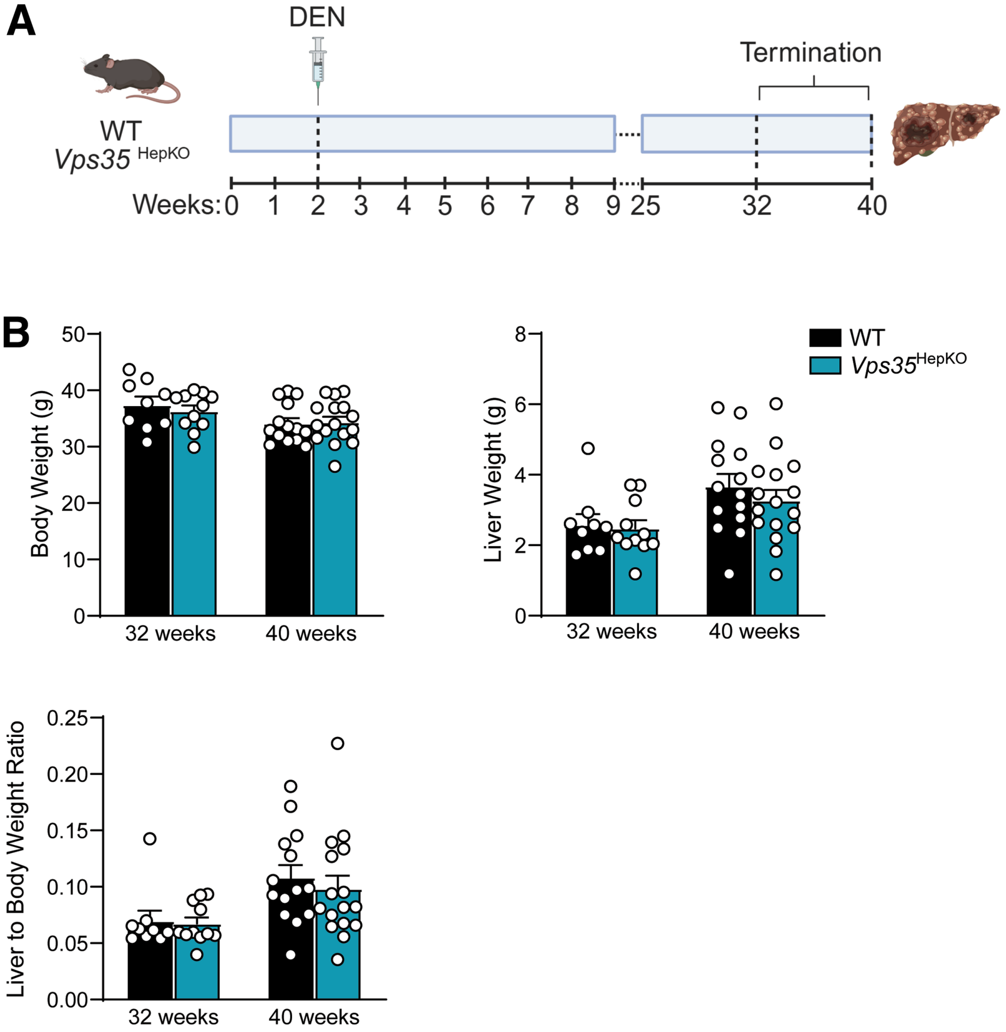

Figure 15. (*A*) **Experimental design of DEN-induced liver cancer.** (*B*) Liver weight, body weight, and liver-to-body weight ratio in 32- and 40-week-old WT and *Vps35*^HepKO^ DEN-administered mice (n = 9–16). WT, wild-type.

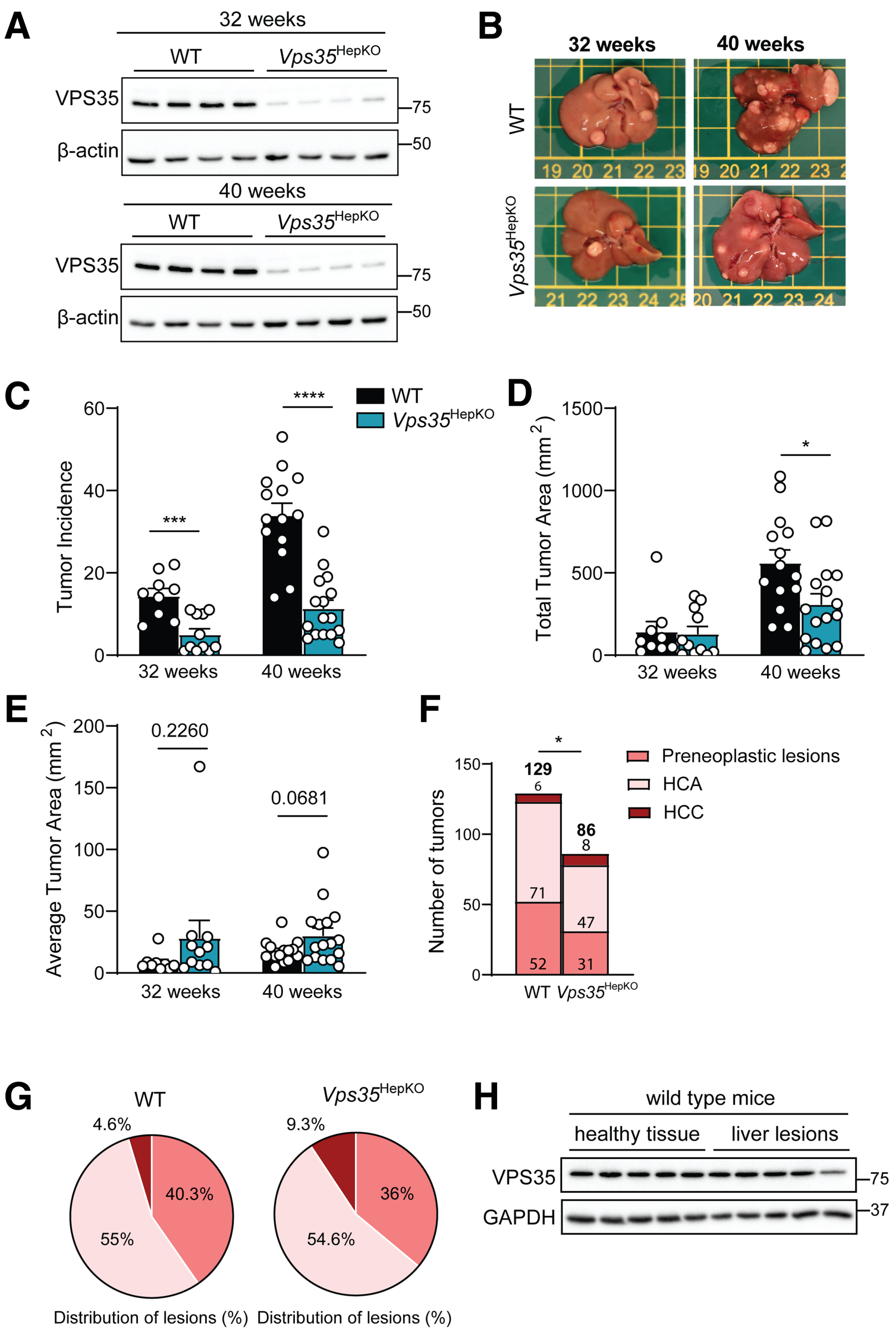

Figure 16. Hepatic VPS35 deficiency mitigates tumor initiation in mice treated with DEN. (*A*) Protein levels of VPS35 in livers from 32- and 40-week-old WT and *Vps35*HepKO DEN-administered male mice. (*B*) Macroscopic representation of tumor burden; grid squares are 1 × 1 cm. (*C–E*) Tumor burden in 32- and 40-week-old WT and *Vps35*HepKO DEN-administered male mice (n = 9–16). (*C*) Tumor incidence, (*D*) total tumor area, and (*E*) average tumor area per lesion (n = 9–16). Data are presented as mean ± SEM; *$P < .05$ and ****$P < .0001$. (*F*) Quantification of the different lesions in 2 liver lobes (right and left lobes) of 40-week-old WT and *Vps35*HepKO DEN-administered male mice (n = 14): preneoplastic lesions, HCAs, and HCC. Statistical significance was determined using negative binomial regression. Data are measured as a count; *$P < .05$. (*G*) Distribution of lesion types in 40-week-old WT and *Vps35*HepKO DEN-administered mice. (*H*) VPS35 levels in healthy liver tissue and liver lesions from 40-week-old DEN-treated WT mice. Protein levels were determined by immunoblotting. HCA, hepatocellular adenoma; SEM, standard error of the mean; WT, wild-type.

birth, the liver undergoes extensive hepatocellular proliferation to reach the liver size needed for body homeostasis.[54] At 4 weeks of age, the liver reaches its adult size in mice, which is associated with a significant drop in proliferating hepatocytes.[55,56] The increased hepatocellular proliferation in *Vps35*HepKO mice might point to an imbalance in postnatal liver growth and maturation. This is supported by the findings that VPS35-deficient livers have reduced albumin expression and increased expression of the YAP pathway. The YAP pathway plays a crucial role in regulating proliferation and differentiation during postnatal liver development,[33] accompanied by the upregulation of cell cycle–related genes, including the E2F transcriptional network, which plays a significant role in coordinating cell cycle progression.[32]

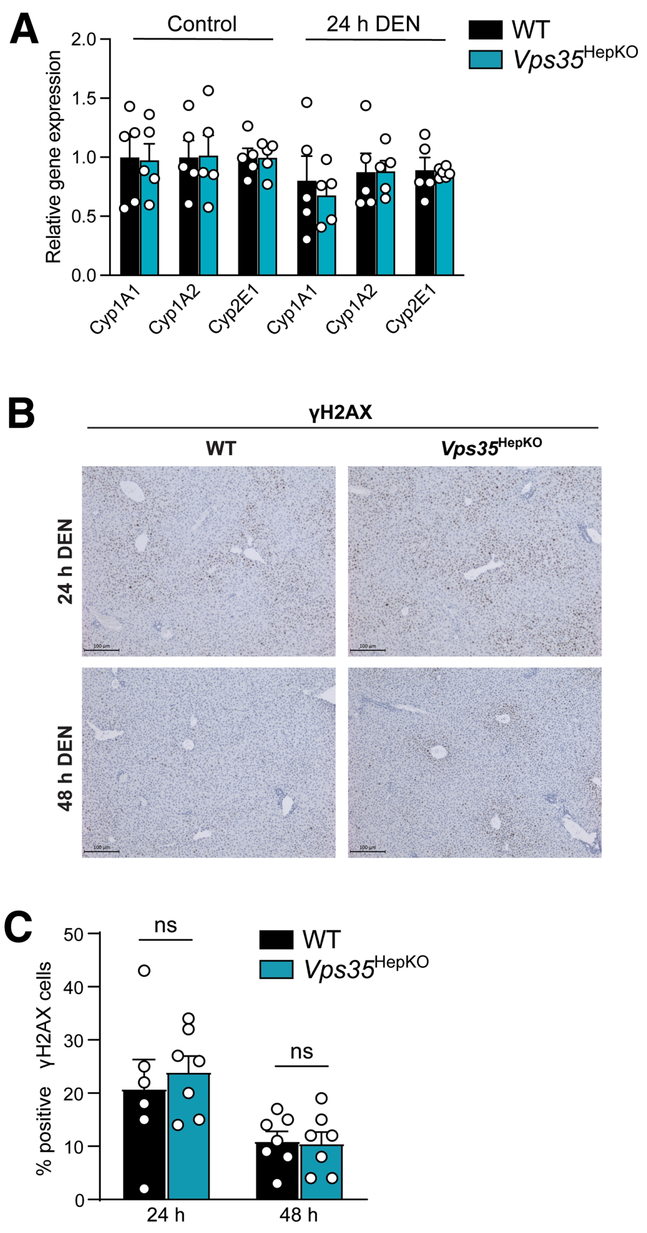

Figure 17. DEN exposure results in comparable hepatic DNA damage and *CYP* expression in 2-week-old WT and *Vps35*HepKO mice. (*A*) Relative hepatic mRNA expression of *Cyp1A1*, *Cyp1A2*, and *Cyp2E1* at the basal state, and at 24 hours post-DEN administration. (*B*) Representative images of WT and *Vps35*HepKO liver sections stained for γH2AX 24 and 48 hours after DEN administration. (*C*) Percentage of gH2AX-positive hepatocytes per section (n = 6–7). Data are presented as mean ± SEM. SEM, standard error of the mean; WT, wild-type.

Remarkably, the increase in hepatocellular proliferation in postnatal VPS35-deficient livers was not observed in middle-aged *Vps35*HepKO mice, suggesting that the imbalance in hepatocellular proliferation in postnatal liver normalizes over time. A similar phenotype has also been reported after hepatic ablation of integrin-linked kinase, where enhanced proliferation was observed in 2-week-old mice but resolved by 30 weeks, coinciding with an 'overcompensated' differentiated state.[39] Integrin-linked kinase is essential for regulating ECM interactions through integrins, emphasizing the role of cell–ECM signaling in postnatal liver development and in sensing the liver's acquired size for body homeostasis.[39] In vitro studies have shown that VPS35 is essential for the endosomal recycling of several integrins, including ITGA5.[20,57,58] Although integrins, including ITGA5, can modulate SRC signaling,[43,59,60] a signaling pathway we found to be elevated in VPS35-deficient livers, and pharmacologic inhibition of this pathway restored hepatocellular proliferation to levels seen in control mice. It is plausible that the loss of VPS35 disrupts integrin-mediated signal transduction during postnatal development. This is supported by the observation that ITGA5 was elevated in *Vps35*HepKO livers compared with control livers, but we cannot rule out a role for other upstream regulators of SRC signaling.

Although the upstream activator(s) of SRC in VPS35-deficient livers remain inconclusive, our results position the SRC signaling as a key pathway promoting the proliferative response after hepatic ablation of VPS35 and highlight the role of the hepatic microenvironment in influencing VPS35 function in mouse livers. Nevertheless, at this moment, we cannot exclude the involvement of additional pathways in this phenotype. Our data, however, suggest that EGFR, IGFR1, ERK, or AKT signaling, all involved in promoting cell proliferation,[61,62] do not play a role in this phenotype, as these pathways were either decreased or unaffected in *Vps35*HepKO mice.

Interestingly, many pathways involved in postnatal liver development also play a role in hepatocellular proliferation during liver regeneration.[63–65] However, our data suggest that the pathway(s) affected by VPS35 deficiency during postnatal liver development are not involved in hepatocellular proliferation during liver regeneration after PH. Alternatively, these pathways are compensated by others, as discussed by Korchilava et al,[66] in which they describe liver regeneration as a redundant and compensatory process governed by numerous signaling pathways. This redundancy may explain why impairment of individual pathways rarely compromises hepatocyte survival or regenerative capacity.[66] As EGFR, an essential player in liver regeneration after PH,[47] is markedly reduced in VPS35-deficient livers and during PH, its reduction is likely compensated through alternative pathways, which are not affected by the loss of VPS35. Collectively, these findings suggest that the effects of VPS35 ablation on hepatocellular proliferation are context-dependent and indicate a role for VPS35 in SRC-mediated proliferation in postnatal livers, whereas during liver regeneration, VPS35-deficient livers can rely on alternative, compensatory pathways that are unaffected by the loss of VPS35. However, further research is required to understand which pathways can compensate for EGFR dysfunction in VPS35-deficient livers.

Previous studies have identified VPS35 as a novel oncogene that promotes the proliferation of hepatoma cells and is associated with poor prognosis in HCC.[15] However, our data suggest that VPS35 does not act as a typical oncogene. Although its loss in hepatocytes reduces tumor initiation, it

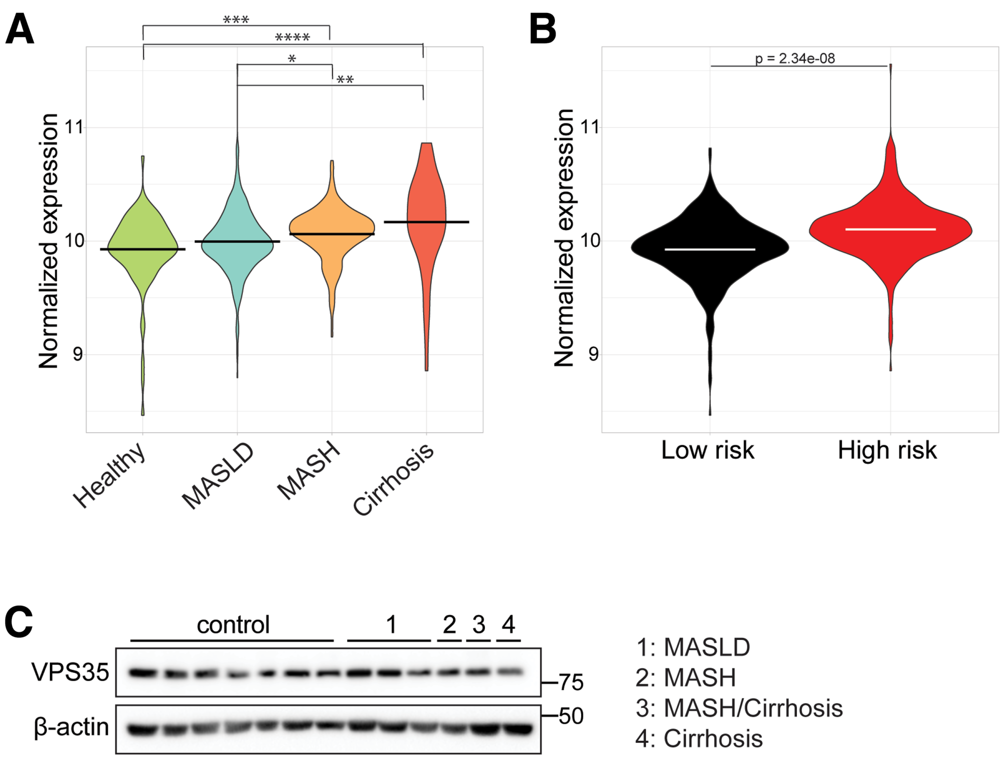

Figure 18. *VPS35* mRNA levels are increased in patients with MASH and cirrhosis. (*A*) *VPS35* mRNA expression across MASLD disease stages (healthy, MASLD, MASH, and cirrhosis) based on analysis of the MegaMASLD database. Adjusted *P* values are shown, with **P* < .05, ***P* < .01, ****P* < .001, and *****P* < .0001. (*B*) *VPS35* mRNA expression according to NMF-based transcriptomic risk stratification from MegaMASLD. Adjusted *P* value is shown. (*C*) VPS35 protein levels in livers from patients with MASLD, MASH, MASH/cirrhosis, or cirrhosis as indicated. NMF, negative matrix factor.

does not suppress tumor progression, unlike classical oncogenes such as the transcription factor c-MYC.[67]

It has been demonstrated that DEN-induced liver cancer in mice is characterized by activation of the Ras/Raf/MEK/ERK pathway and aberrant Wnt signaling due to mutations in genes such as *Hras*, *Braf*, *Egfr*, and *Apc*.[68] In our study, loss of VPS35 reduced the levels of EGFR and ERK signaling, likely due to impaired endosomal recycling of EGFR, resulting in increased lysosomal degradation of EGFR, as shown in previous in vitro studies.[19] Moreover, depletion of VPS35 has been reported to decrease Wnt signaling.[69] Together, these results suggest that VPS35 deficiency restrains these specific signaling pathways and thereby alleviates the initiation of DEN-induced liver lesions.

Interestingly, however, the same pathways activated by DEN are also critical for tumor progression.[69] In our model, VPS35 deficiency did not prevent tumor growth once lesions were established. On the contrary, average lesion size and malignancy tended to be even higher in *Vps35*^{HepKO} mice compared with controls. One explanation is that the lesions in VPS35-deficient livers arise from hepatocytes carrying DEN-induced mutations that activate pathways driving both initiation and progression, which VPS35 does not regulate. For example, to the best of our knowledge, it is unclear whether VPS35 regulates RAS signaling when activated by DEN-induced mutations in *Hras* or *Braf*. Such oncogenic mutations render upstream RTKs inhibition ineffective.[70–72] Because VPS35 acts directly on RTKs, such as EGFR and FGFR3,[15,16] and thus upstream of HRAS and BRAF, it is very unlikely that loss of VPS35 would affect RAS-driven signaling, and therefore, tumor progression would not be alleviated. Nevertheless, if VPS35 expression is elevated in liver tumors, as recently shown by Zhang and colleagues,[15] it may promote numerous oncogenic pathways during tumor progression. This is especially of interest because we found that *VPS35* mRNA levels are increased in liver pathologies such as MASH and cirrhosis, which are predisposed to HCC. Further studies are therefore needed to determine which pathways involved in HCC initiation and progression are regulated when VPS35 expression is increased.

In conclusion, we show that hepatic loss of VPS35 in mice increases hepatocellular proliferation in postnatal livers, likely through the SRC signaling pathway, but does not affect hepatic proliferation after PH. At the same time, it mitigates the initiation of DEN-induced liver lesions but does not substantially affect tumor growth or malignancy, indicating that VPS35 is not a typical oncogene in mice.

Materials and Methods

Animals

Liver-specific *Vps35*-knockout (*Vps35*^{HepKO}) mice were generated as described in our previous study.[23] Mice were individually housed under a 12-hour light-dark cycle with ad libitum access to a standard laboratory diet and water. All animal procedures were approved by the Institutional Animal Care and Use Committee, University of Groningen (Groningen, the Netherlands) and by the Animal Care Committee of the University of Barcelon (Barcelona, Spain).

Mice were fasted for 4 hours before sacrifice. At sacrifice, blood was collected in EDTA-coated tubes by cardiac puncture, and plasma was collected after centrifugation at $1000 \times g$ for 10 minutes at 4 °C. Liver tissue was collected,

snap-frozen in liquid nitrogen, and stored at −80 °C until further analysis. Otherwise, livers were fixed in 4% paraformaldehyde, embedded in paraffin, and cut into 4-μm sections for histologic analysis.

Human Material

Healthy human liver tissue was obtained from 5 anonymous organ donors, TransplantLines METc number 2014-077. The use of human material was approved by the Medical Ethical Committee of the University Medical Center Groningen (UMCG) according to Dutch legislation and the Code of Conduct for responsible handling of human tissue in the context of health research (www.coreon.org) (https://www.coreon.org/wp-content/uploads/2023/06/Code-of-Conduct-for-Health-Research-2022.pdf). Once the tissue was anonymously coded and used for its intended purpose, written consent was not required for additional uses.

Histologic Analysis

Formalin-fixed, paraffin-embedded liver samples were sectioned at 4 μm prior to staining with H&E for histopathologic assessment. To assess for proliferation, immunohistochemical staining for Ki67 (RM-9106, Thermo Fisher Scientific) and BrdU (M0744, Dako) was performed according to standard protocols. Mice received BrdU (50 mg/kg body weight) via an i.p. injection 48 and 24 hours before sacrifice. To quantify the Ki67 and BrdU-positive cells, the number of positive cells was counted in 10 high-power fields with 20× magnification for each liver sample of each experimental group. For each sample, the average number of positive cells per field was calculated and, subsequently, the average per field per experimental group. To evaluate proliferation in livers following PH, immunohistochemical staining for proliferation cell nuclear antigen (M0879, DAKO) was performed and quantitatively analyzed for positive cells via QuPath (version 0.5.1). Mitotic figures were identified based on morphologic features in H&E staining and counted in 10 randomly selected 20× screens on digital slides.

To evaluate cellular senescence, immunohistochemical staining for p21 (ab107099, Abcam) was performed and quantitatively assessed for positive cells. Immunohistochemical staining for cleaved caspase-3 (AF835, R&D Systems) was performed to examine apoptotic cell death and for gH2AX (9718S, Cell Signaling) to visualize DNA damage. Image analysis was performed using QuPath (version 0.5.1). Cells were detected using the positive cell detection tool, with quantification based on the optical density sum of 3, 3'-diaminobenzidine staining. A complete list of antibodies used can be found in Supplementary Table 4. A Leica DM3000 microscope with a mounted Leica DFC420 camera was used for image collection.

Gene Expression Analysis

Homogenization, RNA extraction from liver tissue, and sample preparation for gene expression analysis using qRT-PCR as described in our previous study[23] (under revision in *ATVB*). Gene expression was calculated using the ΔΔCT method using QuantStudio Real-Time PCR Software (Applied Biosystems), with *Ppia* expression as an internal control. Primer sets used for gene expression analysis are listed in Supplementary Table 2. For RNA-seq analysis, 6 representative liver samples were selected, and total RNA was isolated with the RNAeasy plus kit (74,134, Qiagen) according to the manufacturer's instructions. Quantity and quality were determined using nanodrop and gel electrophoresis, respectively. Library preparation, sequencing, and analysis were done by Novogene Co Ltd, Europe. Differential expression analysis was performed using the DESeq2 R package, and the resulting *P* values were adjusted by using the Benjamini-Hochberg method to control the false discovery rate. Genes with adjusted P values $< .05$ were considered as differentially expressed. These data were used for Kyoto Encyclopedia of Genes and Genomes pathway enrichment analysis. RNA-seq data were also used to perform GSEA with GSEA software (Broad Institute Inc and UC San Diego),[73] using Hallmark gene sets[74] and Kyoto Encyclopedia of Genes and Genomes–derived gene sets.[75]

Protein Tyrosine Kinase Assay

Tyrosine activity was determined in mouse liver samples using the protein tyrosine kinase PamChip4 microarray system on the PamStation12 (PamGene International, 's Hertogenbosch, The Netherlands). Each PamChip4 has 4 arrays that contain 144 distinct peptides, serving as protein tyrosine kinase substrates, immobilized on a porous membrane. Frozen liver samples (n = 4) were lysed in T-PER Tissue Protein Extraction reagent (Thermo Fisher Scientific) supplemented with phosphatase inhibitors, followed by freeze-thaw lysis in liquid nitrogen. Next, samples were sonicated with the Sonics Vibra Cell VC375 (8 × 8, 8W pulses for a total of 10 seconds), and centrifuged at 16,000 × *g* for 15 minutes at 4 °C, after which the supernatant was collected. Sample protein concentrations were determined using the BCA protein assay kit (Thermo Fisher Scientific). The assay mixture was prepared according to the manufacturer's instructions, containing 10 μg of protein lysate per array. PamChips were blocked with 2% bovine serum albumin (BSA) for 30 minutes, followed by loading the assay mixtures onto the arrays and kinase activity profiling by PamStation12. BioNavigator software (PamGene) was used to process and analyze the data, and to perform upstream kinase analysis to identify the kinases that are likely responsible for the differences in kinase activity between $Vps35^{HepKO}$ and control livers.

Nuclear Extraction

Nuclear extraction was performed on liver samples using the NE-PER Nuclear and Cytoplasmic Extraction Kit (Thermo Fisher Scientific) according to the manufacturer's instructions. In brief, frozen liver samples were crushed, washed in 1 × Dulbecco's phosphate-buffered saline (PBS), and centrifuged at 500 × *g* for 5 minutes at 4 °C. The pellet was homogenized with the Precellys 24 Bead Beater (6000

rpm, 2 × 15-second cycles, 30 seconds pause, 4 °C) in the appropriate volume of CER I, and was vortexed. After adding CER II, samples were vortexed and centrifuged at 16,000 × *g* for 5 minutes at 4 °C, and the supernatant was taken as the cytosolic fraction. The remaining pellets were suspended in ice-cold nuclear extraction reagent, and were vortexed for 15 seconds every 10 minutes, for a total of 40 minutes. Samples were centrifuged at 16,000 × *g* for 10 minutes at 4 °C. The supernatant (nuclear fraction) was transferred to a clean tube. Both fractions were stored at −80°C until use for immunoblotting (see below for protein measurement and sample preparation).

Membrane Extraction

Subcellular fractions were isolated using the Mem-PER Plus Membrane Protein Extraction kit (Life Technologies). Liver samples weighing between 20 and 40 mg were placed in a 5-mL microcentrifuge tube. Four milliliters of wash solution was added, followed by gentle vortexing, then the wash solution was discarded. The tissue was then transferred to a 2-mL tissue homogenizer, to which 1 mL of permeabilization buffer was added, and the tissue was homogenized with 6 to 10 strokes until a uniform suspension was achieved. An additional 1 mL of permeabilization buffer was mixed into the homogenate and then transferred to a new tube and incubated at 4 °C for 10 minutes with continuous gentle mixing. The permeabilized suspension was centrifuged at 16,000 × g for 15 minutes at 4 °C. The resulting supernatant containing the cytoplasmic fraction was carefully collected to a new tube. The remaining pellet was resuspended in 1 mL of solubilization buffer by pipetting thoroughly. Next, the sample was incubated for 30 minutes at 4 °C with constant mixing. After incubation, the samples were centrifuged once more under the same conditions (16,000 × g for 15 minutes at 4 °C), and the supernatant containing solubilized membrane and membrane-associated proteins was collected in a new tube. Prepared fractions were stored at −80 °C or immediately used for subsequent analysis.

Immunoblotting

Liver and cell samples were homogenized in NP-40 buffer (0.1% Nonidet P-40 [NP-40], 0.4 M NaCl, 10 mM Tris-HCl [pH 8.0], and 1 mM EDTA) supplemented with 1 mM dithiothreitol (DTT) and protease and phosphatase inhibitors (Roche). Protein concentrations were measured with the Bradford assay (Bio-Rad), and samples were diluted in 4× sample buffer. The samples were boiled for 5 minutes at 95 °C prior to loading on gel. Proteins were separated with sodium dodecyl sulfate-polyacrylamide gel electrophoresis and transferred to Immobilon-P polyvinylidene difluoride membranes (Sigma Aldrich). Membranes were blocked in 5% BSA in Tris-buffered saline with 0.01% Tween 20 and incubated with the antibodies indicated in Supplementary Table 3. Proteins were visualized and quantified with the ChemiDoc XRS+ System and Image Lab software version 5.2.1 (Bio-Rad).

Primary Hepatocyte Proliferation Assay

The primary hepatocyte proliferation assay followed the methodology described by Greenhalgh et al.[76] Primary hepatocytes were isolated from adult wild-type and *Vps35*^{HepKO} mice and seeded at a density of 10,000 cells per well in 24-well Primaria tissue culture plates (Cat# 353847; Corning) in Dulbecco's Modified Eagle Medium/Nutrient Mixture F-12 with 15 mmol/L HEPES (H3375; Sigma-Aldrich), 10% fetal bovine serum, 1% insulin-transferrin-selenium (41,400-045; Thermo Fisher Scientific), and 1% penicillin-streptomycin. The cells were allowed to attach for 4 hours at 37 °C in a humidified incubator with 5% CO_2, and then washed with PBS. After adhesion, cells were then cultured in low-serum medium for 48 hours (0.5% fetal bovine serum). Following 48 hours of culture in low serum media, hepatocytes were stimulated with hepatocyte growth factor (PHG0254) and EGF (PMG8044), each at a final concentration of 40 ng/mL. After 24 hours of stimulation, the culture medium was refreshed with growth factors and supplemented with 10 μmol/L EdU (C10640; Thermo Fisher Scientific) to label proliferating hepatocytes. After 48 hours of stimulation, cells were washed in PBS containing 1% BSA and fixed in 4% paraformaldehyde in PBS for 15 minutes at room temperature (RT). Fixed cells were then washed again PBS containing 1% BSA and permeabilized with 0.5% Triton X-100 (T8787; Sigma-Aldrich) in PBS for 20 minutes at RT. Proliferating hepatocytes were labelled with the Click-iT Plus EdU Alexa Fluor 647 Imaging Kit (C10640; Thermo Fisher Scientific). Click-iT Plus reaction cocktail was added and incubated for 30 minutes at RT in the dark. The cells were washed 3 additional times in PBS. Coverslips were mounted using VECTASHIELD Antifade Mounting Medium with 4′,6-diamidino-2-phenylindole (VectorLabs, H-1200-10). Imaging was performed using a confocal laser scanning microscope. The percentage of EdU+ cells was calculated based on the total 4′,6-diamidino-2-phenylindole–positive cells.

Two-Thirds Partial Hepatectomy

Two-thirds of the liver were surgically removed under isoflurane anesthesia following the methodology previously described by Mitchell et al.[77] Mice were sacrificed at 24, 48, and 72 hours post two-thirds partial hepatectomy. Body weight was recorded prior to surgery, and liver weight was determined immediately after resection. Body and liver weight were recorded at termination to establish the liver-to-body weight ratio. Livers were collected and processed for further analysis as mentioned above.

Selective Inhibition of Proto-Oncogene Tyrosine Kinase SRC With Saracatinib (AZD0530)

Eleven-week-old mice were injected intraperitoneally with Sacaratinib (AZD0530, Axon MedChem, 3284) at a concentration of 20 mg/kg for 7 days. Mice were subsequently sacrificed, and liver tissue was collected and processed for further histologic and immunoblotting analysis as previously described.

Diethyl Nitrosamine–Induced Hepatocellular Carcinogenesis

Two-week-old wild-type and *Vps35*^{HepKO} mice were injected i.p. with DEN (Sigma, N0756) at a concentration of 25 mg/kg. To evaluate DEN-induced genotoxicity and its metabolic activation into reactive metabolites, mice were sacrificed at 24- and 48-hours post-DEN administration. For long-term assessment of DEN-induced hepatocarcinogenesis, cohorts were monitored and sacrificed at 32 and 40 weeks to determine tumor burden. Macroscopic liver lesions were recorded to quantify lesion incidence and measured with digital calipers to record the individual and total tumor size per liver. Liver samples were then immediately processed for further histologic analysis as previously mentioned. A veterinary pathologist performed histopathologic analysis to assess the incidence of preneoplastic lesions, hepatocellular adenomas, and HCCs in 2 liver lobes. In all mice, identical liver lobes were characterized. Frozen healthy tissue and lesions of DEN-treated control mice were used to determine VPS35 protein levels in healthy liver tissue and DEN-induced liver lesions through immunoblotting.

Statistical Analysis

Unless indicated otherwise, data are presented as mean ± standard error of the mean. Statistical analyses were performed with GraphPad version 9.1 (GraphPad software). Differences between 2 groups were determined with unpaired 2-tailed Student's *t* test. Count data (eg, total lesion/tumor number) were analyzed using a negative binomial generalized linear model in R version 4.5.1. Significance was assessed using Wald test derived from the model. *P* values of $< .05$ were considered statistically significant.

Supplementary Material

Note: To access the supplementary material accompanying this article, visit the full text version at https://doi.org/10.1016/j.jcmgh.2026.101788.

References

1. Vogel A, Meyer T, Sapisochin G, et al. Hepatocellular carcinoma. Lancet 2022;400:1345–1362.
2. Younossi Z, Stepanova M, Ong JP, et al. Nonalcoholic steatohepatitis is the fastest growing cause of hepatocellular carcinoma in liver transplant candidates. Clin Gastroenterol Hepatol 2019;17:748–755.e3.
3. Haas JT, Francque S, Staels B. Pathophysiology and mechanisms of nonalcoholic fatty liver disease. Annu Rev Physiol 2016;78:181–205.
4. Diehl AM, Day C. Cause, pathogenesis, and treatment of nonalcoholic steatohepatitis. N Engl J Med 2017;377:2063–2072.
5. Kanwal F, Kramer JR, Mapakshi S, et al. Risk of hepatocellular cancer in patients with non-alcoholic fatty liver disease. Gastroenterology 2018;155:1828–1837.e2.
6. Orci LA, Sanduzzi-Zamparelli M, Caballol B, et al. Incidence of hepatocellular carcinoma in patients with nonalcoholic fatty liver disease: a systematic review, meta-analysis, and meta-regression. Clin Gastroenterol Hepatol 2022;20:283–292.e10.
7. Mittal S, El-Serag HB, Sada YH, et al. Hepatocellular carcinoma in the absence of cirrhosis in United States veterans is associated with nonalcoholic fatty liver disease. Clin Gastroenterol Hepatol 2016;14:124–131.e1.
8. Villanueva A. Hepatocellular carcinoma. N Engl J Med 2019;380:1450–1462.
9. Llovet JM, Zucman-Rossi J, Pikarsky E, et al. Hepatocellular carcinoma. Nat Rev Dis Primers 2016;2:16018.
10. Siegel RL, Miller KD, Jemal A. Cancer statistics, 2018. CA Cancer J Clin 2018;68:7–30.
11. Mauro E, de Castro T, Zeitlhoefler M, et al. Hepatocellular carcinoma: epidemiology, diagnosis and treatment. JHEP Rep 2025;7:101571.
12. Vogel A, Finn RS, Blanchet Zumofen MH, et al. Atezolizumab in combination with bevacizumab for the management of patients with hepatocellular carcinoma in the first-line setting: systematic literature review and meta-analysis. Liver Cancer 2023;12:510–520.
13. Chakraborty E, Sarkar D. Emerging therapies for hepatocellular carcinoma (HCC). Cancers (Basel) 2022;14:2798.
14. Finn RS, Qin S, Ikeda M, et al. Atezolizumab plus bevacizumab in unresectable hepatocellular carcinoma. N Engl J Med 2020;382:1894–1905.
15. Zhang G, Tang X, Liang L, et al. DNA and RNA sequencing identified a novel oncogene VPS35 in liver hepatocellular carcinoma. Oncogene 2020;39:3229–3244.
16. Yu J, Feng H, Sang Q, et al. VPS35 promotes cell proliferation via EGFR recycling and enhances EGFR inhibitors response in gastric cancer. EBioMedicine 2023;89:104451.
17. Haft CR, de la Luz Sierra M, Bafford R, et al. Human orthologs of yeast vacuolar protein sorting proteins Vps26, 29, and 35: assembly into multimeric complexes. Mol Biol Cell 2000;11:4105–4116.
18. Burd C, Cullen PJ. Retromer: a master conductor of endosome sorting. Cold Spring Harb Perspect Biol 2014;6:1–14.
19. Yang Z, Feng Z, Li Z, Teasdale RD. Multifaceted roles of retromer in EGFR trafficking and signaling activation. Cells 2022;11:3358.
20. Steinberg F, Gallon M, Winfield M, et al. A global analysis of SNX27-retromer assembly and cargo specificity reveals a function in glucose and metal ion transport. Nat Cell Biol 2013;15:461–471.
21. Seaman MNJ. Retromer and its role in regulating signaling at endosomes. In: Lamaze C, Prior I, eds. Endocytosis and signaling. Progress in molecular and subcellular biology 57. London: Springer International Publishing, 2018:137–150.
22. Sigismund S, Avanzato D, Lanzetti L. Emerging functions of the EGFR in cancer. Mol Oncol 2018;12:3–20.
23. Vos DY, Barbosa MG, Heida AH, et al. The endosomal sorting complex Retromer has a central role in systemic

cholesterol homeostasis by controlling endo-lysosomal cholesterol transport. bioRxiv. 2025.
24. Maeda S, Kamata H, Luo JL, et al. IKKβ couples hepatocyte death to cytokine-driven compensatory proliferation that promotes chemical hepatocarcinogenesis. Cell 2005;121:977–990.
25. Wree A, Johnson CD, Font-Burgada J, et al. Hepatocyte-specific Bid depletion reduces tumor development by suppressing inflammation-related compensatory proliferation. Cell Death Differ 2015;22:1985–1994.
26. Weber A, Boger R, Vick I, et al. Hepatocyte-specific deletion of the antiapoptotic protein myeloid cell leukemia-1 triggers proliferation and hepatocarcinogenesis in mice. Hepatology 2010;51:1226–1236.
27. Krizhanovsky V, Yon M, Dickins RA, et al. Senescence of activated stellate cells limits liver fibrosis. Cell 2008; 134:657–667.
28. Guo Y, Chai B, Jia J, et al. KLF7/VPS35 axis contributes to hepatocellular carcinoma progression through CCDC85C-activated β-catenin pathway. Cell Biosci 2021;11:73.
29. Xu G, Chu J, Shi Y, Huang L, Fu J. The regulation of proliferation and apoptosis in hepatocellular carcinoma via insulin-like growth factor 1 receptor. Growth Horm IGF Res 2022;66:101499.
30. Kent LN, Rakijas JB, Pandit SK, et al. E2f8 mediates tumor suppression in postnatal liver development. J Clin Invest 2016;126:2955–2969.
31. Moreno E, Toussaint MJM, Van Essen SC, et al. E2F7 is a potent inhibitor of liver tumor growth in adult mice. Hepatology 2020;73:303–317.
32. Bertoli C, Skotheim JM, De Bruin RAM. Control of cell cycle transcription during G1 and S phases. Nat Rev Mol Cell Biol 2013;14:518–528.
33. Septer S, Edwards G, Gunewardena S, et al. Yes-associated protein is involved in proliferation and differentiation during postnatal liver development. Am J Physiol Gastrointest Liver Physiol 2012;302:493–503.
34. Dimri M, Satyanarayana A. Molecular signaling pathways and therapeutic targets in hepatocellular carcinoma. Cancers (Basel) 2020;12:491.
35. Johnston PA, Grandis JR. STAT3 signaling: anticancer strategies and challenges. Mol Interv 2011;11:18–26.
36. Lemeer S, Jopling C, Naji F, et al. Protein-tyrosine kinase activity profiling in knock down Zebrafish embryos. PLoS One 2007;2:e581.
37. Darnell JE. STATs and gene regulation. Science 1997; 277:1630–1635.
38. Hoey I, Grusby MJ. STATs as mediators of cytokine-induced responses. Adv Immunol 1998;71:145–162.
39. Gkretsi V, Apte U, Mars WM, et al. Liver-specific ablation of integrin-linked kinase in mice results in abnormal histology, enhanced cell proliferation, and hepatomegaly. Hepatology 2008;48:1932–1941.
40. Wang J, Fedoseienko A, Chen B, et al. Endosomal receptor trafficking: retromer and beyond. Traffic 2018; 19:578–590.
41. Seaman MNJ. The retromer complex – endosomal protein recycling and beyond. J Cell Sci 2012;125(Pt 20):4693–4702.
42. Lu YY, Zhao XK, Yu L, et al. Interaction of Src and Alpha-V integrin regulates fibroblast migration and modulates lung fibrosis in a preclinical model of lung fibrosis. Sci Rep 2017;7:46357.
43. Peng Z, Hao M, Tong H, et al. The interactions between integrin $\alpha_5\beta_1$ of liver cancer cells and fibronectin of fibroblasts promote tumor growth and angiogenesis. Int J Biol Sci 2022;18:5019–5037.
44. Nam HJ, Im SA, Oh DY, et al. Antitumor activity of saracatinib (AZD0530), a c-Src/Abl kinase inhibitor, alone or in combination with chemotherapeutic agents in gastric cancer. Mol Cancer Ther 2013;12:16–26.
45. Du G, Wang J, Zhang T, et al. Targeting Src family kinase member Fyn by Saracatinib attenuated liver fibrosis in vitro and in vivo. Cell Death Dis 2020;11:118.
46. Michalopoulos GK. Liver regeneration after partial hepatectomy. Am J Pathol 2010;176:2–13.
47. Natarajan A, Wagner B, Sibilia M. The EGF receptor is required for efficient liver regeneration. Proc Natl Acad Sci U S A 2007;104:17081–17086.
48. Schulien I, Hasselblatt P. Diethylnitrosamine-induced liver tumorigenesis in mice. Methods Cell Biol 2021; 163:137–152.
49. Lin LL, Kun GL, Ming QX, et al. Altered expression of cytochrome P450 and possible correlation with preneo plastic changes in early stage of rat hepatocarcinogenesis. Acta Pharmacol Sin 2005;26:737–744.
50. Brodeur CM, Thibault P, Durand M, et al. Dissecting the expression landscape of cytochromes P450 in hepatocellular carcinoma: towards novel molecular biomarkers. Genes Cancer 2019;10:97–108.
51. Cheng HS, Chua D, Chan ST, et al, MegaMASLD. An interactive platform for exploring stratified transcriptomic signatures in MASLD progression. bioRxiv. 2024.
52. Zhao Y, Zhao S, Liu S, et al. Kupffer cells, the limelight in the liver regeneration. Int Immunopharmacol 2025;146: 113808.
53. Yin C, Evason KJ, Asahina K, Stainier DYR. Hepatic stellate cells in liver development, regeneration, and cancer. J Clin Invest 2013;123:1902–1910.
54. Grijalva J, Vakili K. Neonatal liver physiology. Semin Pediatr Surg 2013;22:185–189.
55. Berasain C, Avila MA. Regulation of hepatocyte identity and quiescence. Cell Mol Life Sci 2015;72:3831–3851.
56. Liang Y, Kaneko K, Xin B, et al. Temporal analyses of postnatal liver development and maturation by single-cell transcriptomics. Dev Cell 2022;57:398–414.e5.
57. Jia D, Zhang JS, Li F, et al. Structural and mechanistic insights into regulation of the retromer coat by TBC1d5. Nat Commun 2016;7:13305.
58. Zhou Q, Qi F, Zhou C, et al. VPS35 promotes gastric cancer progression through integrin/FAK/SRC signalling-mediated IL-6/STAT3 pathway activation in a YAP-dependent manner. Oncogene 2024;43:106–122.
59. Mitra SK, Schlaepfer DD. Integrin-regulated FAK–Src signaling in normal and cancer cells. Curr Opin Cell Biol 2006;18:516–523.
60. Huveneers S, Danen EHJ. Adhesion signaling – crosstalk between integrins, Src and Rho. J Cell Sci 2009; 122:1059–1069.

61. Wee P, Wang Z. Epidermal growth factor receptor cell proliferation signaling pathways. Cancers (Basel) 2017;9:52.
62. Hua H, Kong Q, Yin J, et al. Insulin-like growth factor receptor signaling in tumorigenesis and drug resistance: a challenge for cancer therapy. J Hematol Oncol 2020;13:64.
63. Michalopoulos GK, Bhushan B. Liver regeneration: biological and pathological mechanisms and implications. Nat Rev Gastroenterol Hepatol 2021;18:40–55.
64. Zhang C, Sun C, Zhao Y, et al. Signaling pathways of liver regeneration: biological mechanisms and implications. iScience 2024;27:108683.
65. Michalopoulos GK, Khan Z. Liver regeneration, growth factors, and amphiregulin. Gastroenterology 2005;128:503–506.
66. Korchilava B, Khachidze T, Megrelishvili N, et al. Liver regeneration after partial hepatectomy: triggers and mechanisms. World J Hepatol 2025;17:107378.
67. Qu A, Jiang C, Cai Y, et al. Role of Myc in hepatocellular proliferation and hepatocarcinogenesis. J Hepatol 2014;60:331–338.
68. Connor F, Rayner TF, Aitken SJ, et al. Mutational landscape of a chemically-induced mouse model of liver cancer. J Hepatol 2018;69:840–850.
69. Liu Y, Deng H, Liang L, et al. Depletion of VPS35 attenuates metastasis of hepatocellular carcinoma by restraining the Wnt/PCP signaling pathway. Genes Dis 2021;8:232–240.
70. Lièvre A, Bachet JB, Boige V, et al. *KRAS* mutations as an independent prognostic factor in patients with advanced colorectal cancer treated with cetuximab. J Clin Oncol 2008;26:374–379.
71. Leiser D, Medová M, Mikami K, et al. KRAS and HRAS mutations confer resistance to MET targeting in preclinical models of MET-expressing tumor cells. Mol Oncol 2015;9:1434–1446.
72. Ou X, Gao G, Habaz IA, Wang Y. Mechanisms of resistance to tyrosine kinase inhibitor-targeted therapy and overcoming strategies. MedComm (2020) 2024;5:e694.
73. Subramanian A, Tamayo P, Mootha VK, et al. Gene set enrichment analysis: a knowledge-based approach for interpreting genome-wide expression profiles. Proc Natl Acad Sci U S A 2005;102:15545–15550.
74. Liberzon A, Birger C, Thorvaldsdóttir H, et al. The molecular signatures database hallmark gene set collection. Cell Syst 2015;1:417–425.
75. Kanehisa M, Sato Y, Kawashima M, et al. KEGG as a reference resource for gene and protein annotation. Nucleic Acids Res 2016;44(D1):D457–D462.
76. Greenhalgh SN, Matchett KP, Taylor RS, et al. Loss of integrin $\alpha v\beta 8$ in murine hepatocytes accelerates liver regeneration. Am J Pathol 2019;189:258–271.
77. Mitchell C, Willenbring H. A reproducible and well-tolerated method for 2/3 partial hepatectomy in mice. Nat Protoc 2008;3:1167–1170.

Received September 20, 2025. Accepted April 8, 2026.

Correspondence
Address correspondence to: Bart van de Sluis, PhD, University Medical Centre Groningen, Antonius Deusinglaan 1, Groningen 9713 AV, The Netherlands. e-mail: a.j.a.van.de.sluis@umcg.nl.

CRediT Authorship Contributions

Markus Barbosa (Conceptualization: Supporting; Formal analysis: Supporting; Methodology: Supporting; Visualization: Lead; Writing – original draft: Lead; Writing – review & editing: Supporting)

Dyonne Vos (Conceptualization: Supporting; Data curation: Supporting; Formal analysis: Supporting; Investigation: Supporting; Visualization: Supporting; Writing – original draft: Equal; Writing – review & editing: Supporting)

Cristy R.C. Verzijl (Data curation: Supporting; Formal analysis: Supporting; Writing – review & editing: Supporting)

Andries Heida (Data curation: Supporting; Formal analysis: Supporting; Investigation: Supporting; Visualization: Supporting; Writing – review & editing: Supporting)

Marco Bakker (Data curation: Supporting; Formal analysis: Supporting; Investigation: Supporting; Visualization: Supporting; Writing – review & editing: Supporting)

Niels Kloosterhuis (Investigation: Supporting; Methodology: Supporting; Supervision: Supporting; Writing – review & editing: Supporting)

Mirjam Koster (Formal analysis: Supporting; Methodology: Supporting)

Marieke Smit (Formal analysis: Supporting; Methodology: Supporting)

Alba Fajardo (Methodology: Supporting)

Dicky Struik (Data curation: Supporting; Formal analysis: Supporting; Methodology: Supporting; Writing – review & editing: Supporting)

Peter Olinga (Resources: Supporting; Writing – review & editing: Supporting)

Johan Jonker (Funding acquisition: Supporting; Methodology: Supporting; Writing – review & editing: Supporting)

Rachel Thomas (Data curation: Supporting; Formal analysis: Supporting; Investigation: Supporting; Methodology: Supporting; Software: Supporting; Validation: Supporting; Visualization: Supporting; Writing – review & editing: Supporting)

Albert Pol (Conceptualization: Supporting; Methodology: Supporting; Resources: Supporting; Supervision: Supporting; Writing – review & editing: Supporting)

Vincent E. de Meijer (Resources: Supporting; Writing – review & editing: Supporting)

Anna Moles (Conceptualization: Supporting; Data curation: Supporting; Methodology: Supporting; Writing – review & editing: Supporting)

Umesh Tharehalli (Conceptualization: Supporting; Investigation: Supporting; Methodology: Supporting; Supervision: Supporting; Writing – review & editing: Supporting)

Jan Albert Kuivenhoven (Conceptualization: Supporting; Funding acquisition: Supporting; Investigation: Supporting; Supervision: Supporting; Writing – review & editing: Supporting)

Bart van de Sluis (Conceptualization: Equal; Funding acquisition: Lead; Methodology: Equal; Resources: Lead; Supervision: Lead; Visualization: Supporting; Writing – original draft: Equal; Writing – review & editing: Lead)

Conflicts of interest
The authors disclose no conflicts.

Funding
This study was financially supported by grants from the European Union (MSCA-ITN-2020, 953489; Acronym EndoConnect) coordinated by Bart van de Sluis; The Netherlands Cardiovascular Research Initiative: ‘the Dutch Heart Foundation, Dutch Federation of University Medical Centers, The Netherlands Organization for Health Research and Development and the Royal Netherlands Academy of Sciences’ (CVON2017-2020; Acronym Genius2) to Jan Albert Kuivenhoven and Bart van de Sluis; Jan Albert Kuivenhoven is an established investigator of the Netherlands Heart Foundation (2015T068); ZonMW OC grant (2471032) to Jan Albert Kuivenhoven and Bart van de Sluis; NWO ENW grants (OCENW.M.22.034 and OCENW.M.23.189) to Bart van de Sluis; Beatrix Kinderziekenhuis (BKZ) grants to Bart van de Sluis and Umesh Tharehalli; The Netherlands Organization for Scientific Research (VICI grant 016.176.640) to Johan W. Jonker; the Graduate School for Drug Exploration (GUIDE); and the De Cock-Hadders Foundation grants to Dyonne Y. Vos and Markus G. Barbosa.

Cellular and Molecular Gastroenterology and Hepatology, Volume *20*

Supplemental information

Vacuolar Protein Sorting 35 Controls Hepatocellular Proliferation Through SRC Signaling and Promotes Diethyl Nitrosamine–Induced Tumor Initiation

Markus G. Barbosa, Dyonne Y. Vos, Cristy R.C. Verzijl, Andries H. Heida, Marco Bakker, Niels J. Kloosterhuis, Mirjam H. Koster, Marieke Smit, Alba Fajardo, Dicky Struik, Peter Olinga, Johan W. Jonker, Rachel Thomas, Albert Pol, Vincent E. de Meijer, Anna Moles, Umesh Tharehalli, Jan Albert Kuivenhoven, and Bart van de Sluis

Supplementary Tables

Supplementary Table 1. Differentially expressed genes related to mechanosensitive transcriptional co-factor YES-associated protein (YAP)-target genes and SRC kinase family genes in *Vps35*^{HepKO} livers compared to controls, as determined with RNAseq analysis.

**Gene**	**Gene name**	**Log2(FC)**	**Adj. *P* value**
Areg	Amphiregulin	3.415	0.003
Birc5	Baculoviral IAP repeat-containing 5	1.417	8.017E-04
Itgβ2	Integrin beta 2	1.429	2.633E-04
Sox9	SRY (sex determining region Y)-box 9	0.968	0.013
Alb	Albumin	-0.444	0.016
Src	Sarcoma oncogene	1.687	5.15E-08
Fgr	FGR proto-oncogene	1.661	2.52E-09
Hck	Hemopoeietic cell kinase	1.453	8.87E-06

Supplementary Table 2. Sequences of primers used for qRT-PCR analysis.

**Gene**	**Forward primer (5'-3')**	**Reverse primer (5'-3')**
Areg	TTGCTGCTGGTCTTAGGCTC	TGGTCCCCAGAAAGCGATTC
Birc5	ATCCACTGCCCTACCGAGAA	CTTGGCTCTCTGTCTGTCCAGTT
Cyr61	AGAGGCTTCCTGTCTTTGGC	CCAAGACGTGGTCTGAACGA
Itgβ2	GCTTTGGGTCGTTTGTGGAC	TGCCGACCTCTGTCTGAAAC
Ppia	TTCCTCCTTTCACAGAATTATTCCA	CCGCCAGTGCCATTATGG
Alb	TGCTTTTTCCAGGGGTGTGTT	TTACTTCCTGCACTAATTTGGCA

Supplementary Table 3. Antibodies used for immunoblotting analysis.

Target	Company	Catalog #	Dilution
AKT	Cell Signaling	4691	1:1000
p-AKT (Ser473)	Cell Signaling	9271	1:1000
Integrin alpha 5 (ITGA5)	Cell Signaling	4705	1:1000
β-actin	Sigma	A5441	1:1000
β-catenin	Abcam	ab6302	1:1000
EGFR	Santa Cruz	sc-373746	1:1000
p-EGFR	Cell Signaling	3777	1:1000
ERK	Cell Signaling	4370	1:1000
p-ERK	Cell Signaling	4695	1:1000
GAPDH	Abcam	ab8245	1:1000
HSP90	Cell Signaling	4874	1:1000
IGFR	Cell Signaling	3027	1:1000
p-IGFR	Cell Signaling	28897	1:1000
Lamin A/C	Cell Signaling	2032	1:1000
p-STAT3 (Tyr705)	Cell Signaling	9145	1:1000
STAT3	Cell Signalling	9139	1:1000
p-SRC (Tyr416)	Cell Signaling	2101	1:1000
SRC	Cell Signaling	2108	1:1000
Vinculin	Abcam	ab129002	1:1000
VPS35	Abcam	ab10099	1:1000
goat anti-mouse IgG (H+L)-HRP conjugate	Bio-Rad	170-6516	1:10000
goat anti-rabbit IgG (H+L)-HRP conjugate	Bio-Rad	170-6515	1:10000

Supplementary Table 4. Antibodies used for histological analysis.

Target	Company	Catalog #	Dilution
BrdU	Dako	M0744	1:800
Ki67	Thermo Fisher	RM-9106	1:50
p21	Abcam	ab107099	1:400
H2AX (ser139)	Cell Signalling	9718S	1:250
PCNA	Dako	M0879	1:600
Active Caspase-3	R&D Systems	AF835	1:600
Goat anti-rabbit (biotinylated)	Vector	BA-1000	1:250
Goat anti-mouse (biotinylated)	Vector	BA-9200	1:125
Goat anti-rat (biotinylated)	Vector	BA-9400	1:125

Figure 2D

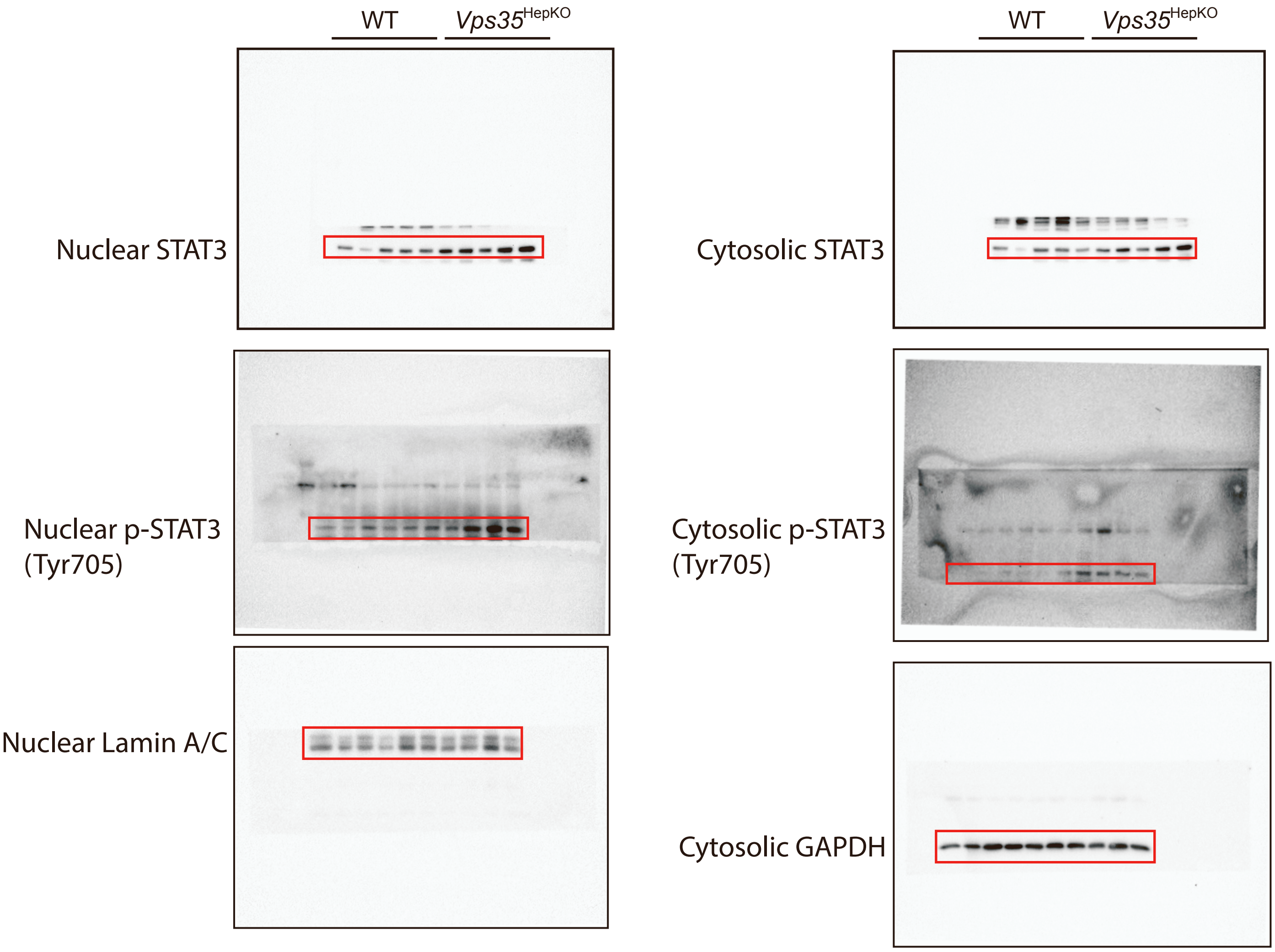

WT
Vps35HepKO
Nuclear STAT3
Nuclear p-STAT3 (Tyr705)
Nuclear Lamin A/C
WT
Vps35HepKO
Cytosolic STAT3
Cytosolic p-STAT3 (Tyr705)
Cytosolic GAPDH

Figure 3C + 3E

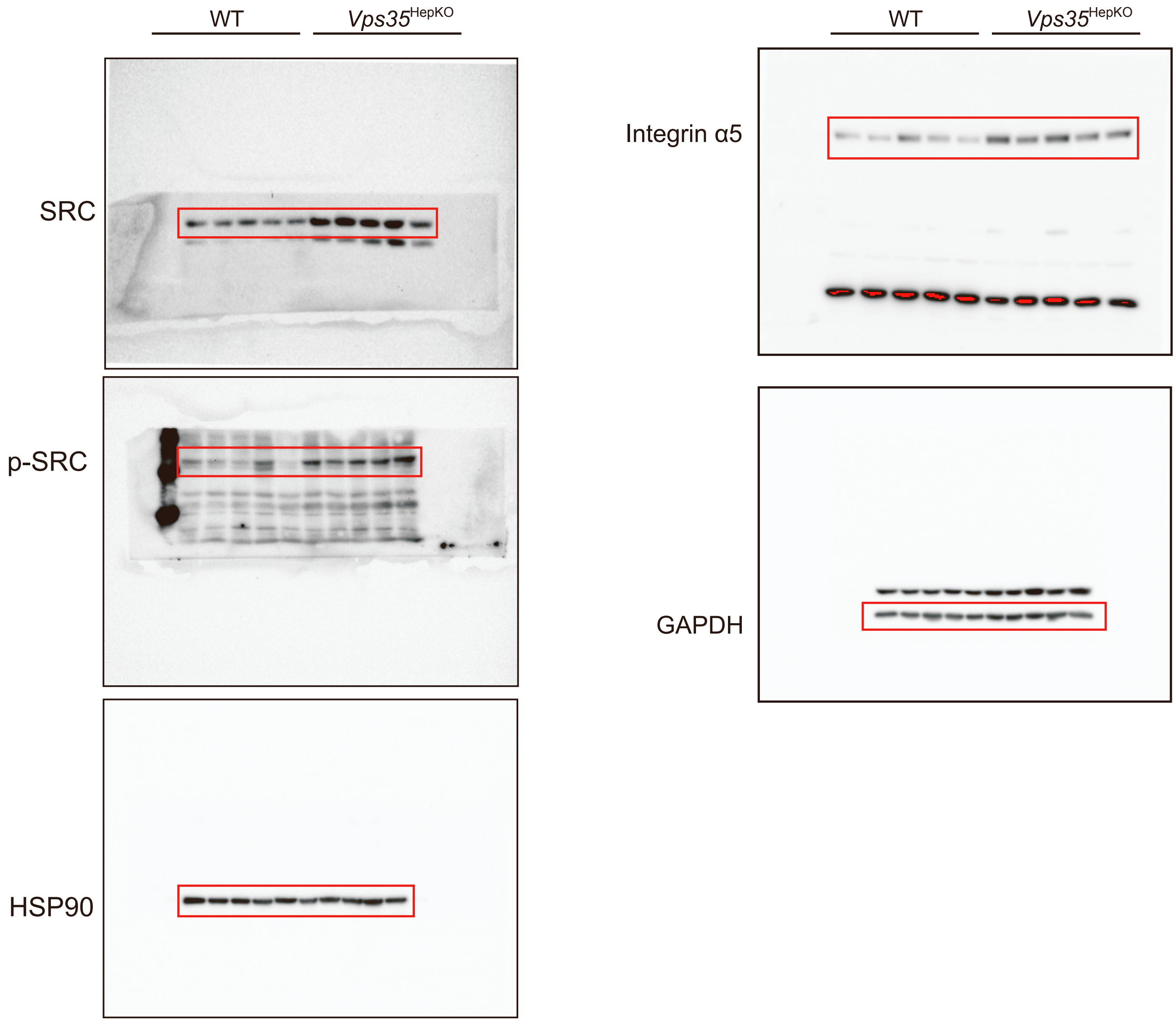
WT
Vps35HepKO
SRC
p-SRC
HSP90
WT
Vps35HepKO
Integrin α5
GAPDH

Figure 4B

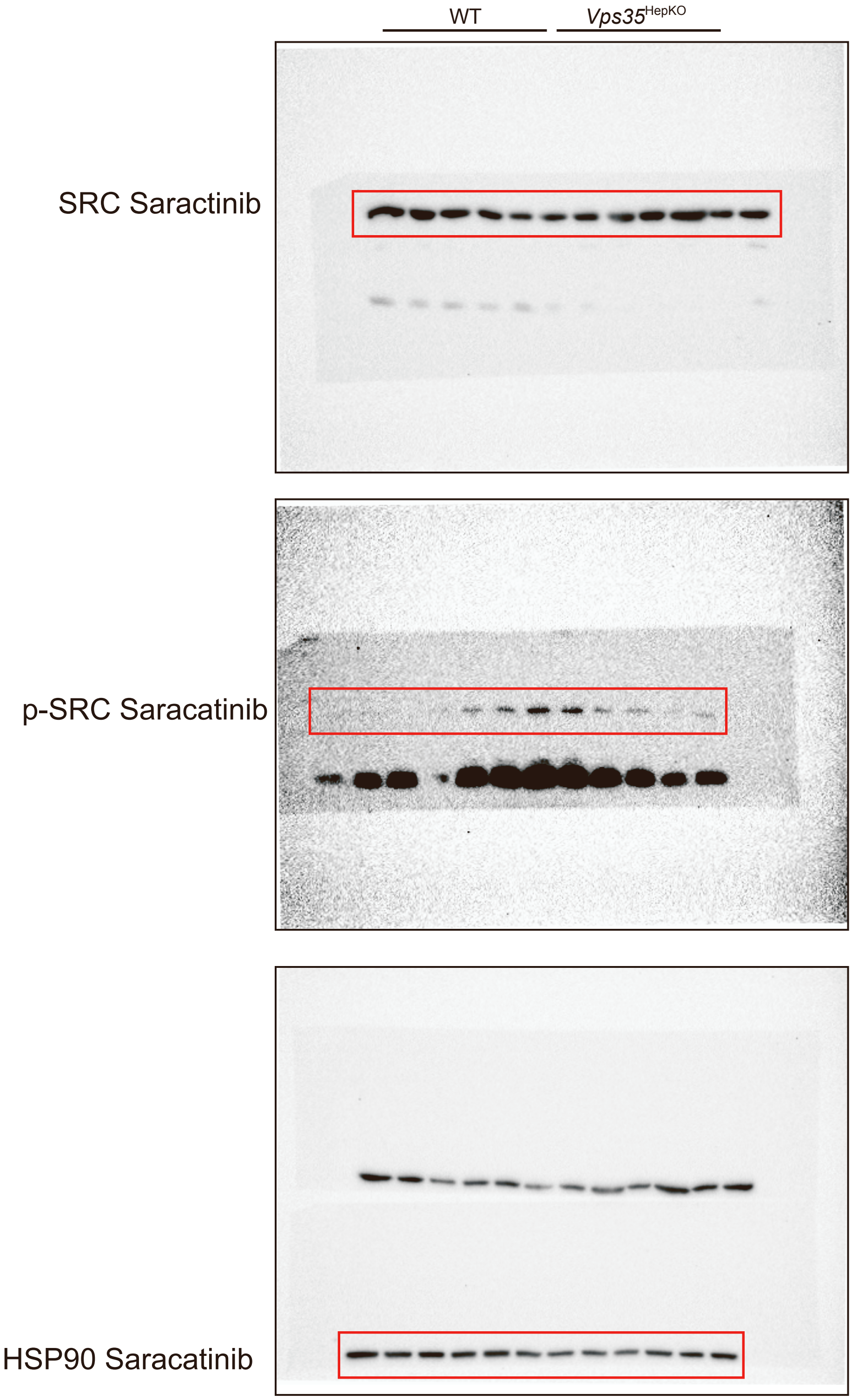
WT
Vps35HepKO
SRC Saractinib
p-SRC Saracatinib
HSP90 Saracatinib

Figure 6A

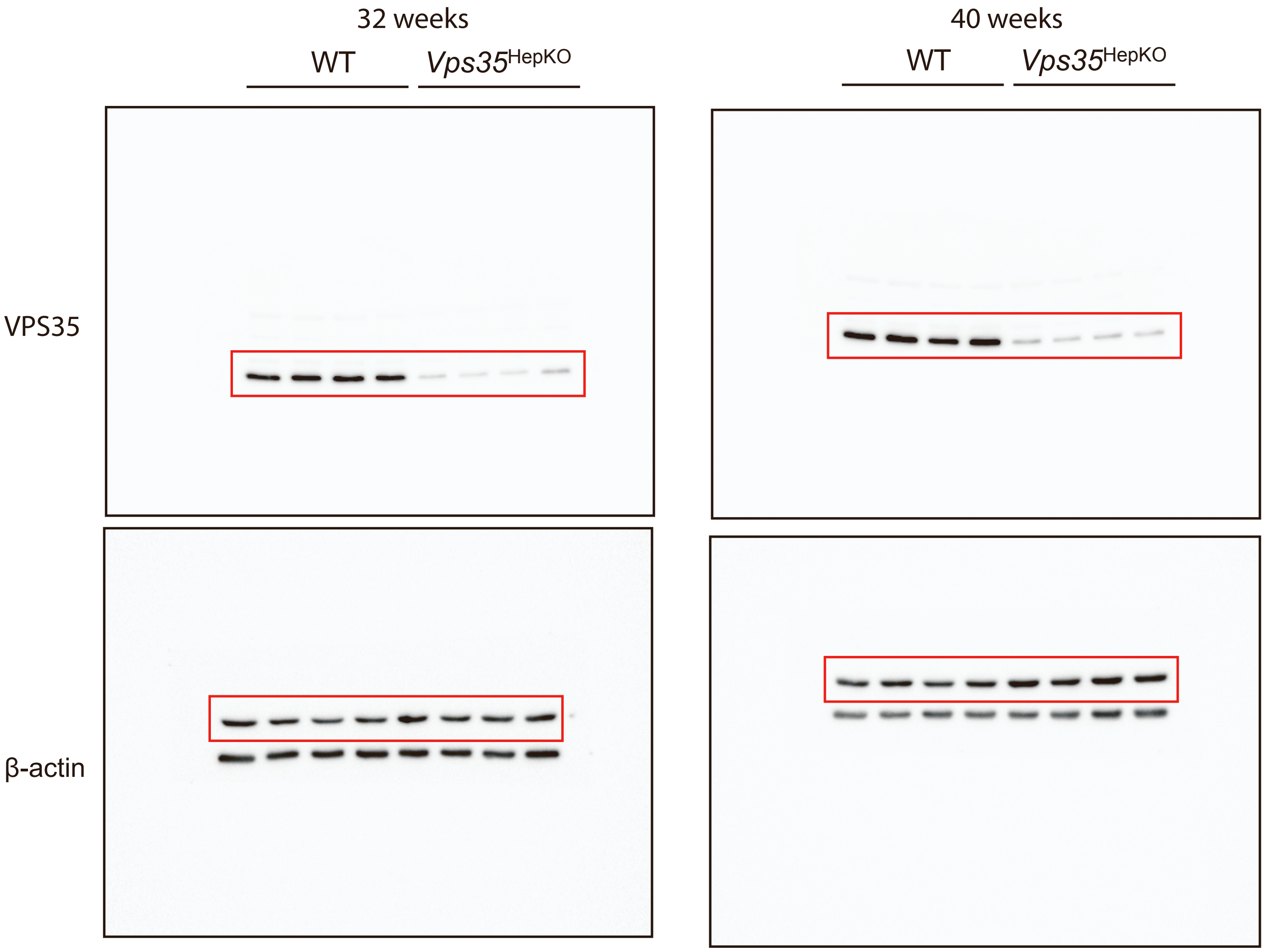

Figure 6H

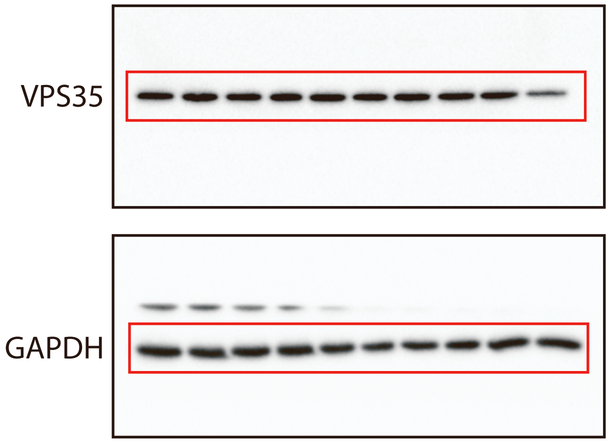

Supplemental Figure 2

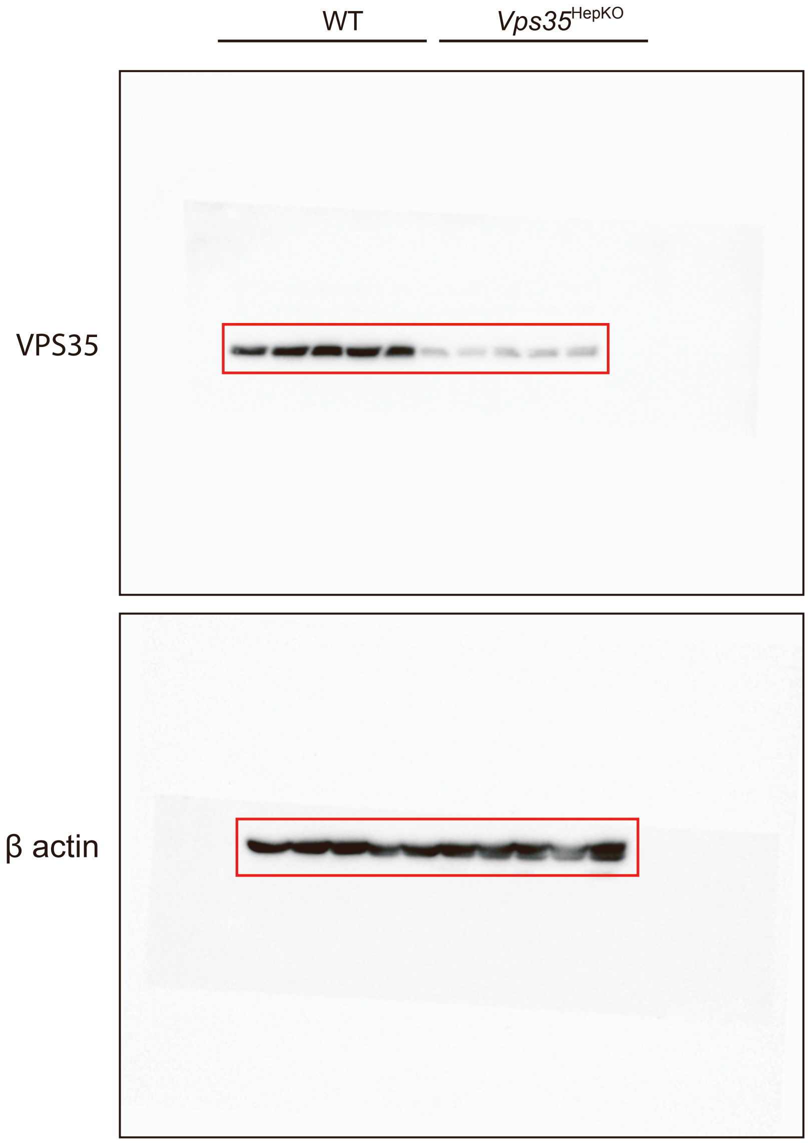

Supplemental Figure 3

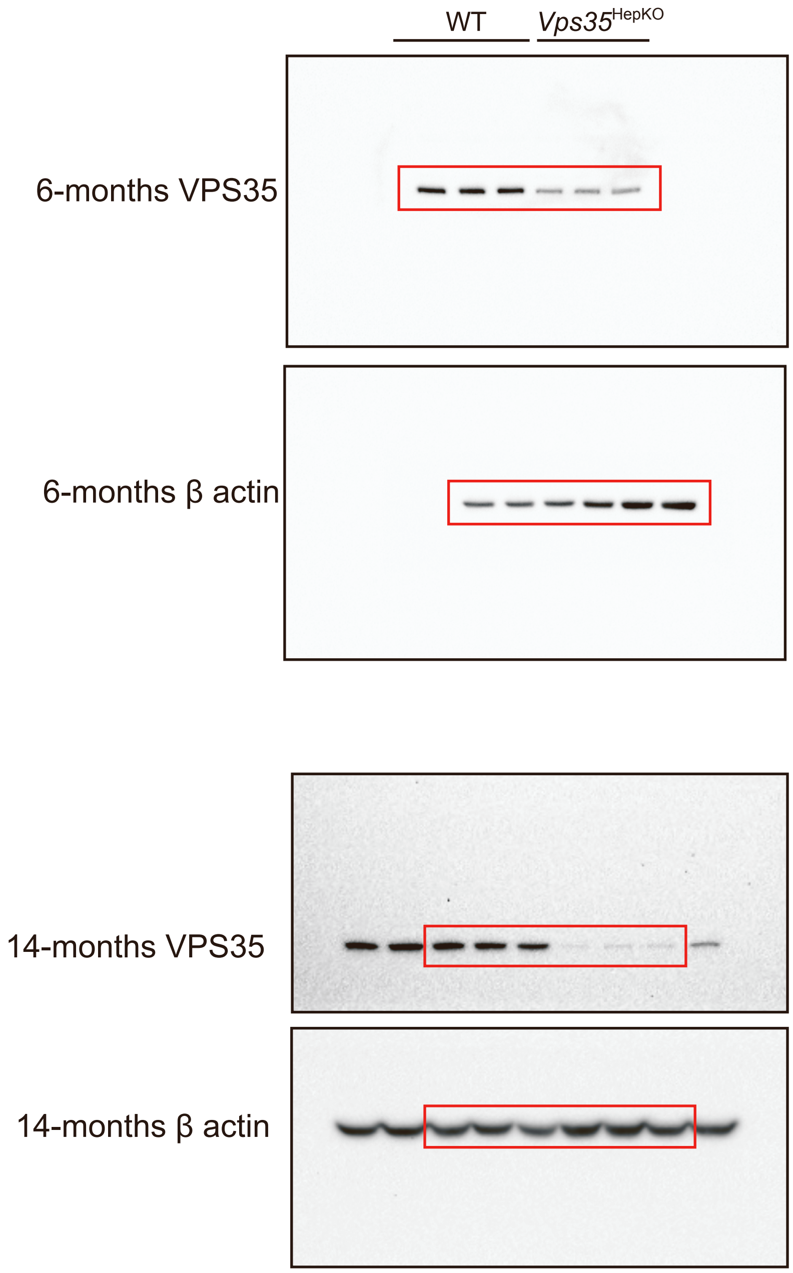

Supplemental Figure 5

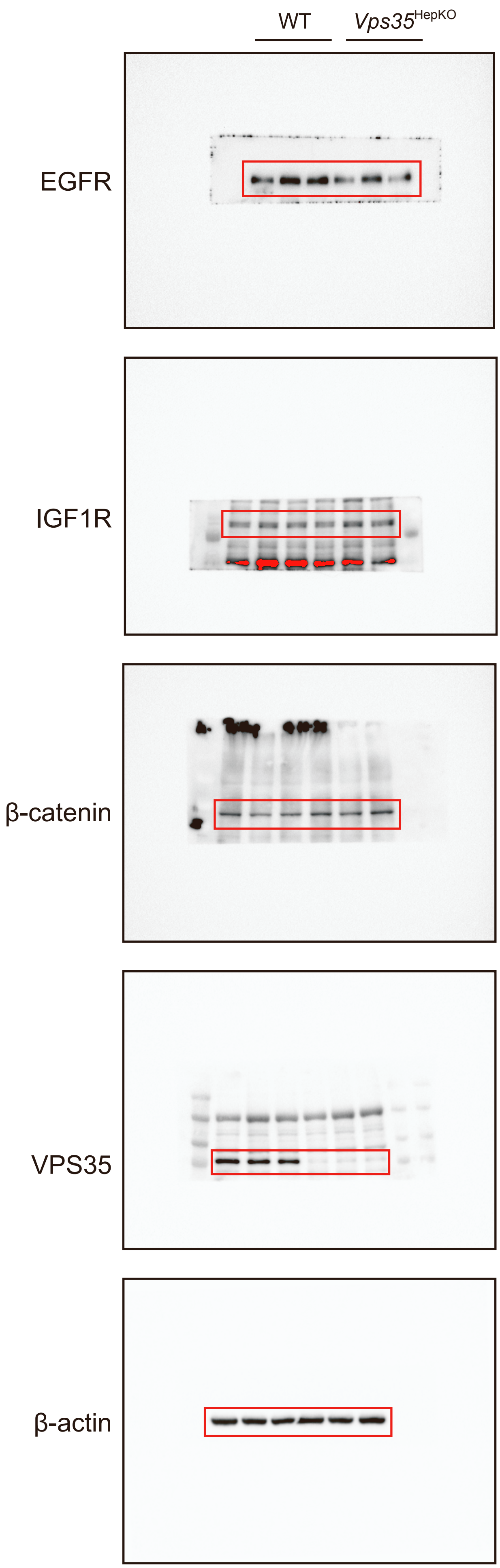

Supplemental Figure 7

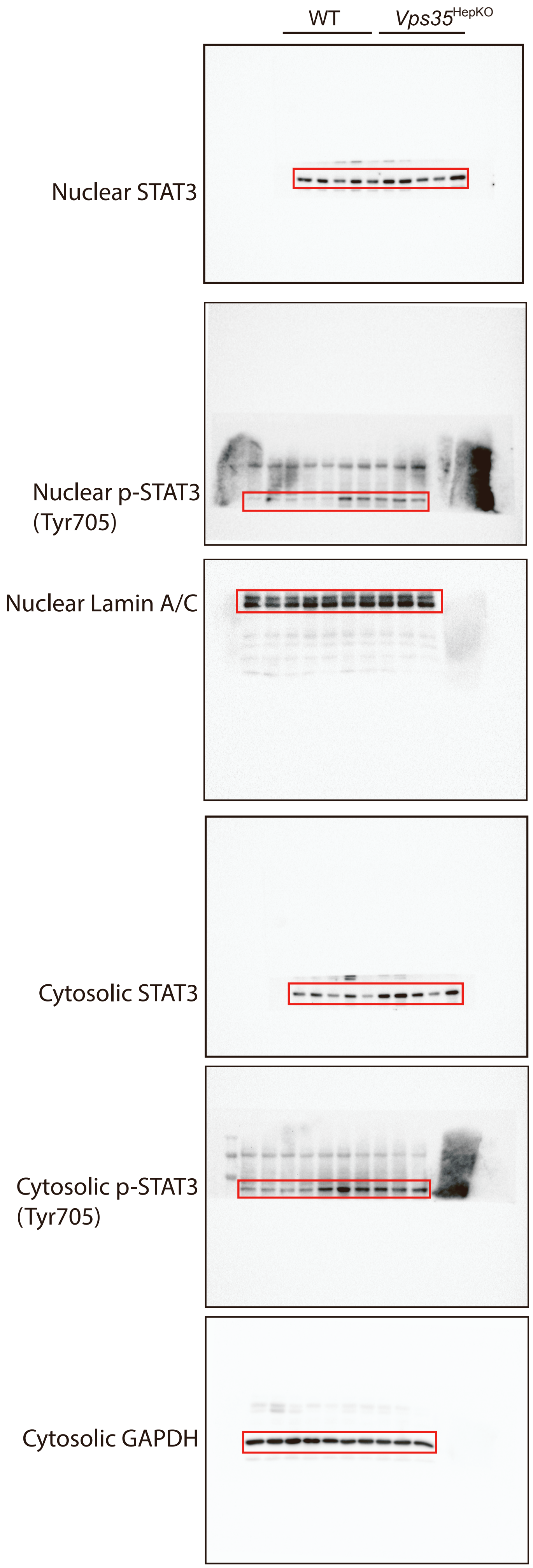

Supplemental Figure 8

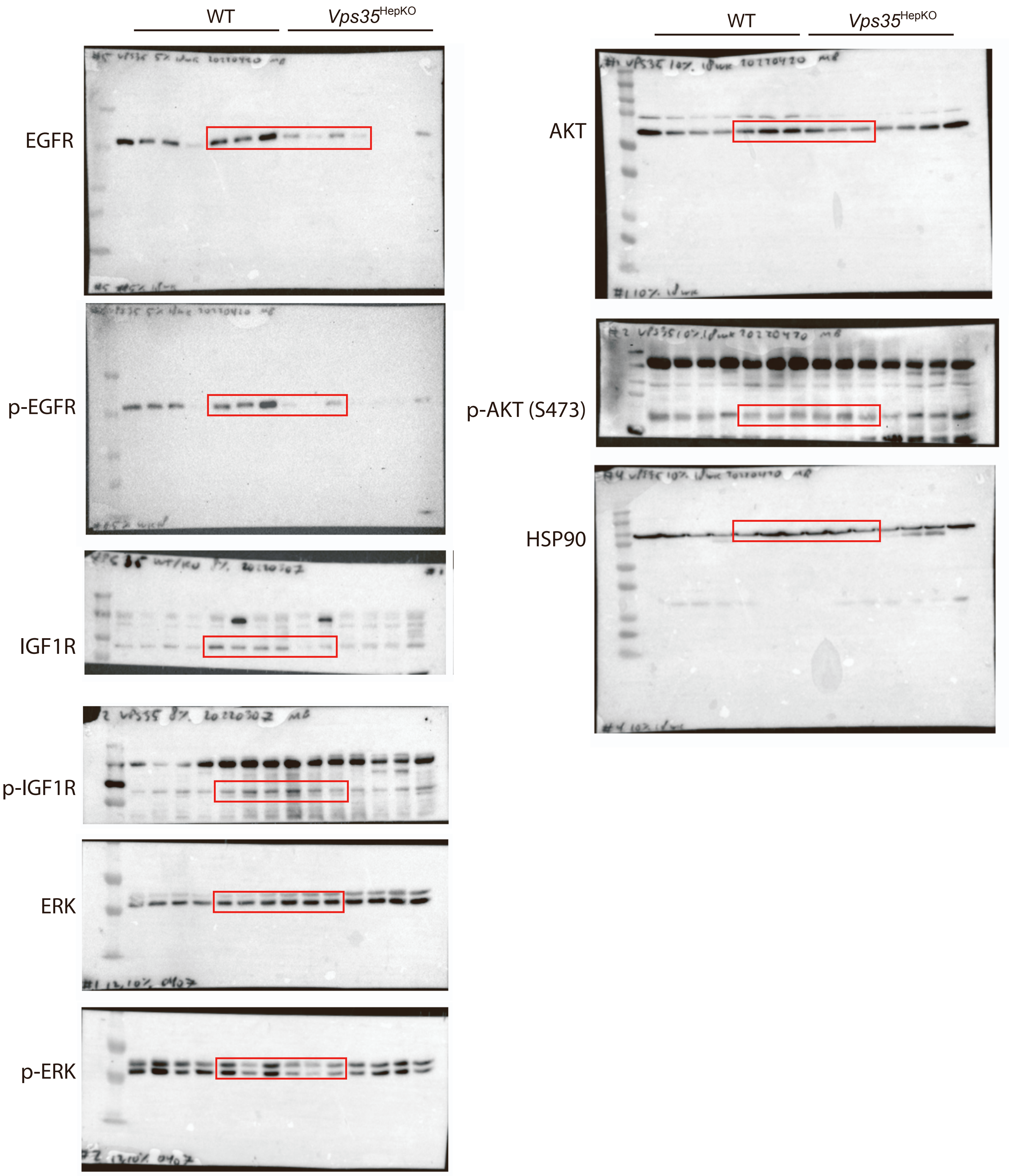

Supplemental Figure 9

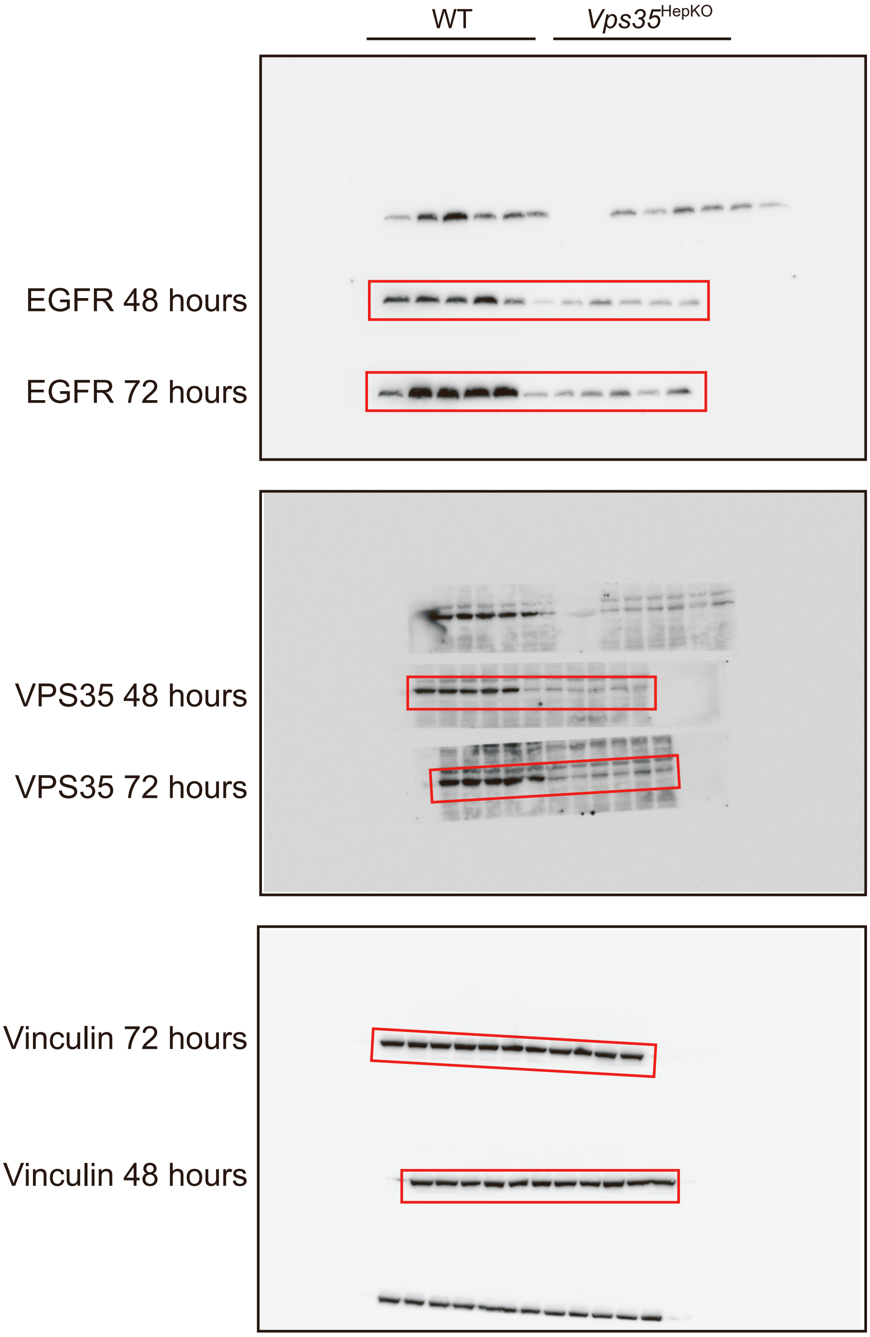

Supplemental Figure 12

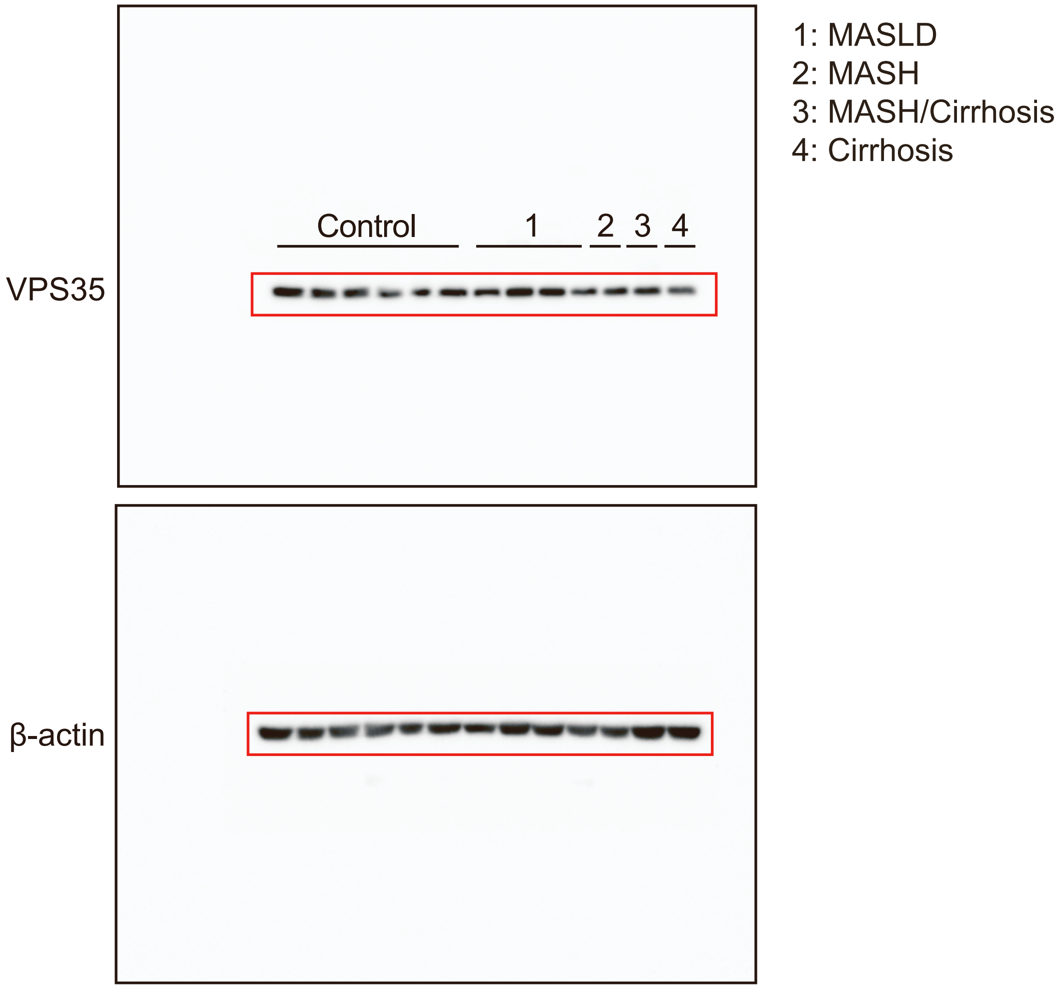

Supplement: Extended PDF [file mmc3.pdf]
